# Supplementary material for: Eliciting initial programme theories for a health research capacity strengthening initiative targeting African universities: A realist synthesis
Source: PLoS One. 2025 Aug 20;20(8):e0330360. doi: 10.1371/journal.pone.0330360 (PMC12367166; doi:10.1371/journal.pone.0330360)
Supplement: S1 File — (DOCX) [file pone.0330360.s001.docx]

**QUALITY AND RELEVANCE APPRAISAL FORM**

| **Record Number:** 01 |
| --- |
| **Data extraction by:** MNM  **Data extraction date:** 6 March 2024 |
| **Title**: Enabling Dynamic Partnerships through Joint Degrees between Low- and High-Income Countries for Capacity Development in Global Health Research: Experience from the Karolinska Institutet/Makerere University Partnership |
| **First author/ year**: Sewankambo et al. (2015) |
| **List companion papers (multiple papers of the same study incl. grey literature)**  None |
| **Relevance and usefulness of this study (see end of the form for definitions)**  High [ ] Moderate [✓] Low [ ] None [ ] |
| **Summary of the Paper**  **(What is this about? What kind of data source? Quant, Qual, MM, Blog, etc.**  The paper does not describe the methodology used, but quantitative data (for individual level capacity outcomes) are presented and highlights of the institutional and network-level research capacities. The journal paper presents the achievement/successes (research capacity outcomes) of a research programme partnership. |
| **What is interesting about this paper?**  The paper describes CMO components that are relevant to research capacity strengthening in the context of an African university. The insights are therefore useful in the theory gleaning process. |
| **In what ways is this article relevant to the candidate programme theories, if at all? (Include specific pages, paragraph, line)**  Contexts   1. The long-term funding arrangement (reliable and sustainable) 2. University strategic role in the national health research system. 3. Inadequate core research infrastructure   Mechanisms   1. The institutional leadership’s willingness to adapt to new ways of doing things (p.5). 2. The inclusion of MoH employees as PhD candidates in the programme has facilitated closer linkages between University x and the MoH – this has created opportunities for research use   Outcomes   1. Spin-off collaborative activities in education and research as the PhDs and MSc trained are teaching in the university and writing grant-winning proposals 2. Long-term funding/collaboration has reduced the risk of brain drain at university to date, all the graduates have stayed in [country] after completing their PhDs 3. Improved research infrastructure [e.g., ICT Master Plan with financial support giving access to scientific journals; establishment of a health and demographic surveillance site (HDSS) and establishment of several research laboratories] 4. University's transition from the traditional doctorate of medicine by monograph to the publication-based PhD with public defence proceedings 5. The long-term funding and collaboration has enabled the university to build a strong, centralized research management system that keeps track of all research funding secured. 6. Research conducted through the collaboration has affected national policies (e.g., neonatal and child health policy revisions in the country x) |
| **What are the strengths and weaknesses of the article? Any red article?**  The paper does not include a description of the study methodology. The paper starts with a description of the programme partnership, then goes on to describe the research capacity outcomes at individual, institutional and network levels. It is, therefore, impossible to assess the quality of the paper and the results since there is no description of study design, methodology or the data sources consulted. Although the paper does not make the C-M-O connections, it provides significant data on context, mechanism and outcomes which can allow/inform the CMO configuration. |
| **Describe any unintended positive or negative impacts and their mechanism link to the outcomes**  The paper reports one of the unintended outcomes: some faculty staff embarking on postdoc research training has strained on teaching and administration in their departments. |

**Definition of the categories**

The following definitions are only examples. These should be modified according to the needs of the study.

**HIGH**: This category is for papers that have high relevance to the realist synthesis. This means that the framing of the research and, the research questions are highly matched to the review questions, the empirical findings are clearly described and there is a rich description of the process and context that can greatly advance the theoretical output of the review. The paper is a ‘key informant’

**MODERATE**: This category is for studies that show a ‘moderately’ relevant framing of the primary research to the review theories. This may mean that the article reports on a different (but related) intervention working toward similar outcomes of interest or describes middle-range theories that may inform the review even if there is no relevant empirical data from the paper to populate the CMO configurations or has a few areas that are of interest even if it is not entirely clear whether they will be used in the synthesis.

**LOW**: This category is for research that has met the selection criteria in terms of relevance to the review questions and the initial programme theories (or MRT) but is relatively thin on the description of context and mechanism. It is not placed in the exclusion category because it contains at least one idea or statement about the context, about the mechanisms or about conceptualizing outcomes that can be used for refining the theory and building a CMO configuration.


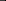


**EXCLUDE**: This category is for a research paper that showed promise on reading the citation, but upon reading the full-text paper does not correspond to the review questions, does not have any content that corresponds to the initial programme theories (or MRT), or does not describe at all the context, or the mechanisms (or process).

| **Record Number:** 02 |
| --- |
| **Data extraction by:** MNM  **Data extraction date**: 7 March 2024 |
| **Title**: Assessing and Strengthening African Universities’ Capacity for Doctoral Programmes |
| **First author/ year**: Bates et al. (2011) |
| **List companion papers (multiple papers of the same study incl. grey literature)**  None |
| **Relevance and usefulness of this study (see end of the form for definitions)**  High [ ] Moderate [✓] Low [ ] None [ ] |
| **Summary of the Paper**  **(What is this about? What kind of data source? Quant, Qual, MM, Blog, etc.**  The paper employed qualitative methods. The authors conducted qualitative interviews with the programme stakeholders. |
| **What is interesting about this paper?**  The paper describes how a capacity strengthneing initiative contributed towards strengtheing the capacities of 19 African researchers and consequently the participating African universities. The paper describes a few CMO components that are relevant to research capacity strengthening in the context of an African university. The insights are therefore useful in the theory gleaning process. |
| **In what ways is this article relevant to the candidate programme theories, if at all? (Include specific pages, paragraph, line)**  Contexts   - Established university policies and regulations governing doctoral programmes - Institutional policies and structures – ethics committee, governance, funding - Research environment – student supervisor ratio, supervisor experience, research facilities and infrastructure, library and academic resources, - Dedicated learning space with internet connectivity   Outcomes   - Institutionalisation of new capacity (individual level capacity used for institutional capacity building objectives) - New funding secured - Research influencing policy and programs   Mechanisms   - Skills and attitudes by the researchers - The ongoing learning and improvement cycle (learning by doing) |
| **What are the strengths and weaknesses of the article? Any red article?**  Although the three case studies had different goals – strengthening research skills (case study 3), strengthening laboratory systems and skills (case study 1), and providing PhD training/skills development (case study 2) – they all focused primarily on capacity strengthening at the institutional level. Individual skills training was complementary, in keeping with the close interdependence between the different levels at which capacity strengthening can occur (i.e., individual, institutional, and national/international levels). Paying attention to the skills of individuals is important even if the main focus of a programme is to strengthen institutional systems and processes (p. 7). These insights are useful in the theory gleaning process. |
| **Describe any unintended positive or negative impacts and their mechanism link to the outcomes**  None |

**Definition of the categories**

The following definitions are only examples. These should be modified according to the needs of the study.

**HIGH**: This category is for papers that have high relevance to the realist synthesis. This means that the framing of the research and, the research questions are highly matched to the review questions, the empirical findings are clearly described and there is a rich description of the process and context that can greatly advance the theoretical output of the review. The paper is a ‘key informant’

**MODERATE**: This category is for studies that show a ‘moderately’ relevant framing of the primary research to the review theories. This may mean that the article reports on a different (but related) intervention working toward similar outcomes of interest or describes middle-range theories that may inform the review even if there is no relevant empirical data from the paper to populate the CMO configurations or has a few areas that are of interest even if it is not entirely clear whether they will be used in the synthesis.

**LOW**: This category is for research that has met the selection criteria in terms of relevance to the review questions and the initial programme theories (or MRT) but is relatively thin on the description of context and mechanism. It is not placed in the exclusion category because it contains at least one idea or statement about the context, about the mechanisms or about conceptualizing outcomes that can be used for refining the theory and building a CMO configuration.


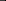


**EXCLUDE**: This category is for a research paper that showed promise on reading the citation, but upon reading the full-text paper does not correspond to the review questions, does not have any content that corresponds to the initial programme theories (or MRT), or does not describe at all the context, or the mechanisms (or process).

| **Record Number:** 03 |
| --- |
| **Data extraction by:** MNM  **Data extraction date:** 8 March 2024 |
| **Title**: Doctoral Education and Institutional Research Capacity Strengthening: An Example at Makerere University in Uganda (2000–2013) |
| **First author/ year**: Akuffo et al. (2014) |
| **List companion papers (multiple papers of the same study incl. grey literature)**  None |
| **Relevance and usefulness of this study (see end of the form for definitions)**  High [ ] Moderate [✓] Low [ ] None [ ] |
| **Summary of the Paper**  **(What is this about? What kind of data source? Quant, Qual, MM, Blog, etc.**  The paper involved a review of evaluation reports and files (not specified whether Quant or Qual) |
| **What is interesting about this paper?**  The paper describes a case study illustrating how a department within an African university has contributed to transforming the post-graduate educational process with formation of a productive research team comprised of faculty and students to advance a research agenda targeting strategic priorities of national significance. The authors make recommendations about the Ph.D. programme, recruitment and supervision, international collaborations, and research infrastructure and environment for building institutional research capacity. The insights are therefore useful in the theory gleaning process. |
| **In what ways is this article relevant to the candidate programme theories, if at all? (Include specific pages, paragraph, line)**  Context   1. Modern technology supporting open sharing of information — for research and for administrative accountability 2. Research infrastructure and research environment 3. Lack of local (government) funding towards research and research training.   Mechanism   1. Openness and rigour of the research leaders influences productivity and fosters long-term relationship with other researchers (this can unlock funding opportunities) 2. Appreciation and respect for local knowledge and priorities by international collaborators determines the effectiveness of the collaboration   Outcomes   1. Increased supervisory capacity (staff who could supervise Ph.D. students increased through training and promotions) 2. Institutional research seminars organised for all research staff and trainees 3. Research career pathways and promotions defined 4. Improved research infrastructure (e.g., laboratories and units developed and equipped) 5. A multidisciplinary research team established 6. Additional funding secured 7. External collaborations developed and strengthened 8. Research graduates taken research leadership position in the university and the Ministry of Health |
| **What are the strengths and weaknesses of the article? Any red article?**  Although the paper describes a few and fragmented C, M and O elements, the CMO components are relevant to research capacity strengthening in the context of an African university and therefore useful to the theory gleaning process. |
| **Describe any unintended positive or negative impacts and their mechanism link to the outcomes**  None |

**Definition of the categories**

The following definitions are only examples. These should be modified according to the needs of the study.

**HIGH**: This category is for papers that have high relevance to the realist synthesis. This means that the framing of the research and, the research questions are highly matched to the review questions, the empirical findings are clearly described and there is a rich description of the process and context that can greatly advance the theoretical output of the review. The paper is a ‘key informant’

**MODERATE**: This category is for studies that show a ‘moderately’ relevant framing of the primary research to the review theories. This may mean that the article reports on a different (but related) intervention working toward similar outcomes of interest or describes middle-range theories that may inform the review even if there is no relevant empirical data from the paper to populate the CMO configurations or has a few areas that are of interest even if it is not entirely clear whether they will be used in the synthesis.

**LOW**: This category is for research that has met the selection criteria in terms of relevance to the review questions and the initial programme theories (or MRT) but is relatively thin on the description of context and mechanism. It is not placed in the exclusion category because it contains at least one idea or statement about the context, about the mechanisms or about conceptualizing outcomes that can be used for refining the theory and building a CMO configuration.


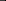


**EXCLUDE**: This category is for a research paper that showed promise on reading the citation, but upon reading the full-text paper does not correspond to the review questions, does not have any content that corresponds to the initial programme theories (or MRT), or does not describe at all the context, or the mechanisms (or process).

| **Record Number:** 04 |
| --- |
| **Data extraction by:** MNM  **Data extraction date:** 9 March 2024 |
| **Title**: Promoting sustainable research partnerships: a mixed-method evaluation of a United Kingdom–Africa capacity strengthening award scheme |
| **First author/ year**: Dean et al. (2015) |
| **List companion papers (multiple papers of the same study incl. grey literature)**  None |
| **Relevance and usefulness of this study (see end of the form for definitions)**  High [ ] Moderate [✓] Low [ ] None [ ] |
| **Summary of the Paper**  **(What is this about? What kind of data source? Quant, Qual, MM, Blog, etc.**  A mixed-method retrospective evaluation approach; a cross-sectional survey administered to all award holders. |
| **What is interesting about this paper?**  The paper describes CMO components that are relevant to research capacity strengthening in the context of an African university. The paper draws out lessons for establishing and maintaining successful research collaborations, based on partnership dynamics, from the perspectives of both HIC and LMIC stakeholders through the evaluation of a research capacity strengthening partnership award scheme. The insights are therefore useful in the theory gleaning process |
| **In what ways is this article relevant to the candidate programme theories, if at all? (Include specific pages, paragraph, line)**  **Context**   1. Financial control or differing expectations of partners. 2. Power dynamics between Global North and South partners as grants are managed/coordinated from the North (the PIs were always based in the UK institutions and Co-PIs were always based in African institutions) 3. Pre-existing relationships between partners, such as a former PhD supervisor student relationship, often resulted in more successful project outcomes 4. Lack or limited autonomy by African institutions in relation to when and how funds were spent. 5. African partner’s financial control determines how equitable the partnership is. 6. Achieving equitable partnerships difficult with new partnerships (no prior collaborations) 7. Elements needed to strengthen ‘research culture’ at institutions (i.e., protected time) lacking 8. Impact on ‘research culture’ was described as being most noticeable when mid-level post-doctoral researchers were involved in partnerships   **Mechanism**   1. Previous working relationships, for example supervisor-student relationships 2. Equity within partnerships linked to partnership formation and experience 3. prestige of the award benefitted their institutions’ reputation (prestige of the award allowed them to engage more senior staff within the institution) 4. effective communication essential for research collaboration 5. Positive impact on research culture through exposure to international collaboration and funding opportunities, as well as opportunity to develop confidence to produce scientific outputs. 6. Mid-level post-doctoral researchers have the time to invest in the research process as well as the energy and motivation to influence change at the institutional level   **Outcomes**   1. Joint publication of research papers 2. Collaborative research grants 3. Strengthening ‘research culture’ 4. Joint PhD supervision 5. Joint conference presentations and paper publications 6. Collaboration benefits such as additional general financial support and sharing of lab space, research space and equipment. |
| **What are the strengths and weaknesses of the article? Any red article?**  The paper provides a detailed description of the study design and methods. A mixed-method retrospective evaluation approach; a cross-sectional survey administered to all award holders. These details can help assess the quality of the study design and the methodological choices made by the researchers. The paper covers both research institutions and universities; it is difficult to isolate evidence that is relevant to universities since the findings were presented as ‘institutional’ capacity strengthening.  Although the paper describes a few and fragmented C, M and O elements (without illustrating the link between the elements), the CMO components are relevant to research capacity strengthening in the context of an African university and therefore useful to the theory gleaning process. |
| **Describe any unintended positive or negative impacts and their mechanism link to the outcomes**  None |

**Definition of the categories**

The following definitions are only examples. These should be modified according to the needs of the study.

**HIGH**: This category is for papers that have high relevance to the realist synthesis. This means that the framing of the research and, the research questions are highly matched to the review questions, the empirical findings are clearly described and there is a rich description of the process and context that can greatly advance the theoretical output of the review. The paper is a ‘key informant’

**MODERATE**: This category is for studies that show a ‘moderately’ relevant framing of the primary research to the review theories. This may mean that the article reports on a different (but related) intervention working toward similar outcomes of interest or describes middle-range theories that may inform the review even if there is no relevant empirical data from the paper to populate the CMO configurations or has a few areas that are of interest even if it is not entirely clear whether they will be used in the synthesis.

**LOW**: This category is for research that has met the selection criteria in terms of relevance to the review questions and the initial programme theories (or MRT) but is relatively thin on the description of context and mechanism. It is not placed in the exclusion category because it contains at least one idea or statement about the context, about the mechanisms or about conceptualizing outcomes that can be used for refining the theory and building a CMO configuration.


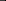


**EXCLUDE**: This category is for a research paper that showed promise on reading the citation, but upon reading the full-text paper does not correspond to the review questions, does not have any content that corresponds to the initial programme theories (or MRT), or does not describe at all the context, or the mechanisms (or process).

| **Record Number:** 05 |
| --- |
| **Data extraction by:** MNM  **Data extraction date:** 11 March 2024 |
| **Title**: Supporting early career health investigators in Kenya: A qualitative study of HIV/AIDS research capacity building |
| **First author/ year**: Daniels et al. (2015) |
| **List companion papers (multiple papers of the same study incl. grey literature)**  None |
| **Relevance and usefulness of this study (see end of the form for definitions)**  High [✓] Moderate [ ] Low [ ] None [ ] |
| **Summary of the Paper**  **(What is this about? What kind of data source? Quant, Qual, MM, Blog, etc.**  The paper employed qualitative methods to examine how collaborative training and research can support early career investigators in an African country. The authors conducted a total of 10 in-depth interviews independent investigators to understand what factors influenced early career investigator development of trainees and how these influenced the transfer plans of this training program to University X. While the small sample of respondents raises concerns, the authors did not discuss any limitations of the study. |
| **What is interesting about this paper?**  The paper describes CMO components that are relevant to research capacity strengthening in the context of an African university. The insights are therefore useful in the theory gleaning process. |
| **In what ways is this article relevant to the candidate programme theories, if at all? (Include specific pages, paragraph, line)**  Context   1. Institutional demands on faculty to teach rather than complete research restricted researchers ability to develop research careers. 2. Lack of local funding to support research. 3. Few investigators interested in research and able to mentor early career investigators. 4. Limited research culture in national institutions 5. Highly trained researchers leaving the country (brain drain)   Mechanism   1. Shared and mutually beneficial resources within international research collaborations are required to support early career investigators and plans to transfer health research training to African institutions.   Outcomes   1. Researchers build institutional capacities following mentorship and provision of transitional funding 2. International training programs developed and institutionalised following sharing of international and local resources |
| **What are the strengths and weaknesses of the article? Any red article?**  The study relies primarily on the perspectives of PIs. The study included purposive sampling methods and a semi-structured protocol to conduct in-depth interviews with US (N=5) and Kenyan (N=5) independent investigators. The voices of other stakeholders are not included, and this could potentially be biased.  C, M and O elements relevant to institutional RCS reported. Although the paper describes a few and fragmented C, M and O elements (without illustrating the link between the elements), the CMO components are relevant to research capacity strengthening in the context of an African university and therefore useful to the theory gleaning process. |
| **Describe any unintended positive or negative impacts and their mechanism link to the outcomes**  None |

**Definition of the categories**

The following definitions are only examples. These should be modified according to the needs of the study.

**HIGH**: This category is for papers that have high relevance to the realist synthesis. This means that the framing of the research and, the research questions are highly matched to the review questions, the empirical findings are clearly described and there is a rich description of the process and context that can greatly advance the theoretical output of the review. The paper is a ‘key informant’

**MODERATE**: This category is for studies that show a ‘moderately’ relevant framing of the primary research to the review theories. This may mean that the article reports on a different (but related) intervention working toward similar outcomes of interest or describes middle-range theories that may inform the review even if there is no relevant empirical data from the paper to populate the CMO configurations or has a few areas that are of interest even if it is not entirely clear whether they will be used in the synthesis.

**LOW**: This category is for research that has met the selection criteria in terms of relevance to the review questions and the initial programme theories (or MRT) but is relatively thin on the description of context and mechanism. It is not placed in the exclusion category because it contains at least one idea or statement about the context, about the mechanisms or about conceptualizing outcomes that can be used for refining the theory and building a CMO configuration.


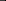


**EXCLUDE**: This category is for a research paper that showed promise on reading the citation, but upon reading the full-text paper does not correspond to the review questions, does not have any content that corresponds to the initial programme theories (or MRT), or does not describe at all the context, or the mechanisms (or process).

| **Record Number:** 06 |
| --- |
| **Data extraction by:** MNM  **Data extraction date:** 12 March 2024 |
| **Title**: Qualitative study to develop processes and tools for the assessment and tracking of African institutions’ capacity for operational health research |
| **First author/ year**: Wallis et al. (2017) |
| **List companion papers (multiple papers of the same study incl. grey literature)**  None |
| **Relevance and usefulness of this study (see end of the form for definitions)**  High [ ] Moderate [✓] Low [ ] None [ ] |
| **Summary of the Paper**  **(What is this about? What kind of data source? Quant, Qual, MM, Blog, etc.**  The paper employed qualitative methods. It tested an evidence-informed process for assessing health research management and support systems (RMSS) in four African universities and for tracking interventions to address capacity gaps. |
| **What is interesting about this paper?**  The paper describes CMO components that are relevant to research capacity strengthening in the context of an African university. The insights are therefore useful in the theory gleaning process |
| **In what ways is this article relevant to the candidate programme theories, if at all? (Include specific pages, paragraph, line)**  **Context**   - Institutional support services and infrastructure - Lack of research support offices and/or insufficient coordination between departments and university levels - Lack of resourcing and long-term financial sustainability of research support offices - Research laboratory facilities are not accredited - Library facilities and resources - Senior researchers spend a substantial proportion of time on administrative and procurement issues that could be more effectively taken on by nonacademic staff - Human resource management for research (lack of clarity on contractual arrangements and no formal postdoctoral career posts for researchers) |
| **What are the strengths and weaknesses of the article? Any red article?**  C, M and O elements relevant to institutional RCS reported. Although the paper describes only context elements (without illustrating the link between the elements), the context components are relevant to research capacity strengthening in the African university settings and therefore useful to the theory gleaning process.  The paper describes the reported research management and support systems (RMSS) gaps and proposed actions/interventions without describing/highlighting the outcomes. Four African universities with 83 staff and students from 11 cadres. Literature-informed ‘benchmark’ was developed and used to itemise all components of a university’s health RMSS. Data on all components were collected during site visits to four African universities using interview guides, document reviews and facilities observation guides. Gaps in RMSS capacity were identified against the benchmark and institutional action plans developed to remedy gaps. Progress against indicators was tracked over 15 months and common challenges and successes identified. |
| **Describe any unintended positive or negative impacts and their mechanism link to the outcomes**  None |

**Definition of the categories**

The following definitions are only examples. These should be modified according to the needs of the study.

**HIGH**: This category is for papers that have high relevance to the realist synthesis. This means that the framing of the research and, the research questions are highly matched to the review questions, the empirical findings are clearly described and there is a rich description of the process and context that can greatly advance the theoretical output of the review. The paper is a ‘key informant’

**MODERATE**: This category is for studies that show a ‘moderately’ relevant framing of the primary research to the review theories. This may mean that the article reports on a different (but related) intervention working toward similar outcomes of interest or describes middle-range theories that may inform the review even if there is no relevant empirical data from the paper to populate the CMO configurations or has a few areas that are of interest even if it is not entirely clear whether they will be used in the synthesis.

**LOW**: This category is for research that has met the selection criteria in terms of relevance to the review questions and the initial programme theories (or MRT) but is relatively thin on the description of context and mechanism. It is not placed in the exclusion category because it contains at least one idea or statement about the context, about the mechanisms or about conceptualizing outcomes that can be used for refining the theory and building a CMO configuration.


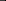


**EXCLUDE**: This category is for a research paper that showed promise on reading the citation, but upon reading the full-text paper does not correspond to the review questions, does not have any content that corresponds to the initial programme theories (or MRT), or does not describe at all the context, or the mechanisms (or process).

| **Record Number:** 07 |
| --- |
| **Data extraction by:** MNM  **Data extraction date:** 13 March 2024 |
| **Title**: Workshop-based learning and networking: a scalable model for research capacity strengthening in low- and middle-income countries |
| **First author/ year**: Perier et al. (2022) |
| **List companion papers (multiple papers of the same study incl. grey literature)**  None |
| **Relevance and usefulness of this study (see end of the form for definitions)**  High [ ] Moderate [✓] Low [ ] None [ ] |
| **Summary of the Paper**  **(What is this about? What kind of data source? Quant, Qual, MM, Blog, etc.**  The paper employed mixed methods (surveys and interviews) to examine how a research programme was contributing to research capacity strengthening of an African university in infectious disease and molecular biology research. |
| **What is interesting about this paper?**  The programme’s approach nucleates training of larger and more diverse groups of students, development of mentoring and bi-directional institutional research partnerships, and support of the local economy. |
| **In what ways is this article relevant to the candidate programme theories, if at all? (Include specific pages, paragraph, line)**  Outcomes   1. Resulted collaborative research grants 2. Institutionalised training at university X – a traininig initially delivered through the XY partnership 3. Laboratory equipment and resources have been sent and remain at university for use by staff and researchers 4. Equal partnership (in design and implementation of the project)   Context   1. Locally driven research and research capacity strengthening agenda 2. Limited research capacity and access to state-of-the-art technologies 3. Limited funding   Mechanism   1. Motivation, willingness and opportunities for researchers to put their acquired skills into practice |
| **What are the strengths and weaknesses of the article? Any red article?**  C, M and O elements relevant to institutional RCS reported. Although the paper describes a few and fragmented C, M and O elements (without illustrating the link between the elements), the CMO components are relevant to research capacity strengthening in the context of an African university and therefore useful to the theory gleaning process. |
| **Describe any unintended positive or negative impacts and their mechanism link to the outcomes**  None |

**Definition of the categories**

The following definitions are only examples. These should be modified according to the needs of the study.

**HIGH**: This category is for papers that have high relevance to the realist synthesis. This means that the framing of the research and, the research questions are highly matched to the review questions, the empirical findings are clearly described and there is a rich description of the process and context that can greatly advance the theoretical output of the review. The paper is a ‘key informant’

**MODERATE**: This category is for studies that show a ‘moderately’ relevant framing of the primary research to the review theories. This may mean that the article reports on a different (but related) intervention working toward similar outcomes of interest or describes middle-range theories that may inform the review even if there is no relevant empirical data from the paper to populate the CMO configurations or has a few areas that are of interest even if it is not entirely clear whether they will be used in the synthesis.

**LOW**: This category is for research that has met the selection criteria in terms of relevance to the review questions and the initial programme theories (or MRT) but is relatively thin on the description of context and mechanism. It is not placed in the exclusion category because it contains at least one idea or statement about the context, about the mechanisms or about conceptualizing outcomes that can be used for refining the theory and building a CMO configuration.


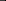


**EXCLUDE**: This category is for a research paper that showed promise on reading the citation, but upon reading the full-text paper does not correspond to the review questions, does not have any content that corresponds to the initial programme theories (or MRT), or does not describe at all the context, or the mechanisms (or process).

| **Record Number:** 08 |
| --- |
| **Data extraction by:** MNM  **Data extraction date:** 14 March 2024 |
| **Title**: Experiences of capacity strengthening in sanitation and hygiene research in Africa and Asia: the SHARE Research Consortium |
| **First author/ year**: Torondel et al. (2019) |
| **List companion papers (multiple papers of the same study incl. grey literature)**  None |
| **Relevance and usefulness of this study (see end of the form for definitions)**  High [ ] Moderate [✓] Low [ ] None [ ] |
| **Summary of the Paper**  **(What is this about? What kind of data source? Quant, Qual, MM, Blog, etc.**  A reflective paper/ commentary piece describing how a research programme has contributed towards increasing the capacity of individuals and institutions from low- and middle-income countries. Qualitative data primarily referenced. |
| **What is interesting about this paper?**  The paper illustrates the link between individual level research capacity and institutional capacity; demonstrating how individual researchers can translate their research capacity to benefit their universities. In Phase II of the initiative, the aim was to support capacity development of a smaller number of African research institutions (including university) to move towards their independent sustainability, with a stronger focus on early and midcareer scientists within these institutions. Phase II constituted a transition from phase I and aimed to increase the programme’s focus on building capacity in the target countries. This has required a transition away from investing in PhDs and MScs towards more established mid-career scientists from within the partner institutions. This move aimed to enhance the retention of skills and knowledge within leading national institutions while also maximising the reach of investment – mid-career scientists themselves offer capacity development and support to PhD and MSc students. |
| **In what ways is this article relevant to the candidate programme theories, if at all? (Include specific pages, paragraph, line)**  **Context**   - Strategic national partners with basic systems and structures in place - Locally driven research agendas ensured buy-in - Dedicated technical support and networking - Driven by local initiatives tailored with support from partners - Sensitive to institutional operations to support both the research and management capacities.   **Mechanism**   - Researchers’ motivation for research - Researchers learn by doing thus acquiring hands-on research experience   **Outcomes**   - Formation of a peer network of researchers was the pivot of success (institutionalising capacity) - All networks aimed to orientate early- and middle-career researchers and equip them with scientific research skills |
| **What are the strengths and weaknesses of the article? Any red article?**  C, M and O elements relevant to institutional RCS reported. The main strategy was to strengthen institutional (African universities) capacities through postgraduate training and establishing peer network of researchers. These researchers would subsequently institutionalise research capacity in their universities. Although the paper describes a few and fragmented C, M and O elements (without illustrating the link between the elements), the CMO components are relevant to research capacity strengthening in the context of an African university and therefore useful to the theory gleaning process. |
| **Describe any unintended positive or negative impacts and their mechanism link to the outcomes**  None |

**Definition of the categories**

The following definitions are only examples. These should be modified according to the needs of the study.

**HIGH**: This category is for papers that have high relevance to the realist synthesis. This means that the framing of the research and, the research questions are highly matched to the review questions, the empirical findings are clearly described and there is a rich description of the process and context that can greatly advance the theoretical output of the review. The paper is a ‘key informant’

**MODERATE**: This category is for studies that show a ‘moderately’ relevant framing of the primary research to the review theories. This may mean that the article reports on a different (but related) intervention working toward similar outcomes of interest or describes middle-range theories that may inform the review even if there is no relevant empirical data from the paper to populate the CMO configurations or has a few areas that are of interest even if it is not entirely clear whether they will be used in the synthesis.

**LOW**: This category is for research that has met the selection criteria in terms of relevance to the review questions and the initial programme theories (or MRT) but is relatively thin on the description of context and mechanism. It is not placed in the exclusion category because it contains at least one idea or statement about the context, about the mechanisms or about conceptualizing outcomes that can be used for refining the theory and building a CMO configuration.


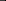


**EXCLUDE**: This category is for a research paper that showed promise on reading the citation, but upon reading the full-text paper does not correspond to the review questions, does not have any content that corresponds to the initial programme theories (or MRT), or does not describe at all the context, or the mechanisms (or process).

| **Record Number:** 09 |
| --- |
| **Data extraction by:** MNM  **Data extraction date:** 15 March 2024 |
| **Title**: From local to global: a qualitative review of the multi-leveled impact of a multi-country health research capacity development partnership on maternal health in Sudan |
| **First author/ year**: Elmusharaf et al. (2016) |
| **List companion papers (multiple papers of the same study incl. grey literature)**  None |
| **Relevance and usefulness of this study (see end of the form for definitions)**  High [✓] Moderate [ ] Low [ ] None [ ] |
| **Summary of the Paper**  **(What is this about? What kind of data source? Quant, Qual, MM, Blog, etc.**  Both quantitative and qualitative data is presented. The authors reflect on the impact of the [x] research partnership on health research capacity development throughout the life of the project |
| **What is interesting about this paper?**  The authors reflect on the project in one of its’ partner countries over its’ five year duration. The reflection is supported by specific project data collected over the period of the project (2008–2014). The data collected included: (i) 6 monthly and annual donor reports; (ii) a mid-term internal and end of project independent evaluation of the entire project, and; (ii) a Ph.D study conducted by a member of the research team. |
| **In what ways is this article relevant to the candidate programme theories, if at all? (Include specific pages, paragraph, line)**  Context   1. The political, historical, and infrastructural context of country X meant that the initial plans for partnering as laid out in the research proposal in country X had to change. 2. Multistakeholder engagement national ministry of health and international NGOs 3. MOUs between organisations served the purpose of getting data on [disease area] for the country X knowledge synthesis report and facilitated training of postgraduate students.   Mechanisms   1. Signing of a Memorandum of Understanding at country level between the Ministry of Health, research and academic institutions in country X 2. The establishment of country level initiatives and a research unit which has been recognized globally through awards and membership in global health forums   Outcomes   1. Technical working group was formed and lead by University X 2. Memorandum of Understanding between University X and the national Ministry of Health enabled access to documents needed for the knowledge synthesis. 3. National knowledge synthesis report on country's health system developed and disseminated 4. Identification of country level health systems research priorities 5. Training and graduation of a team member with a Ph.D. 6. Collaborations established |
| **What are the strengths and weaknesses of the article? Any red article?**  C, M and O elements relevant to institutional RCS reported. Although the paper describes a few and fragmented C, M and O elements (without illustrating the link between the elements), the CMO components are relevant to research capacity strengthening in the context of an African university and therefore useful to the theory gleaning process. |
| **Describe any unintended positive or negative impacts and their mechanism link to the outcomes**  None |

**Definition of the categories**

The following definitions are only examples. These should be modified according to the needs of the study.

**HIGH**: This category is for papers that have high relevance to the realist synthesis. This means that the framing of the research and, the research questions are highly matched to the review questions, the empirical findings are clearly described and there is a rich description of the process and context that can greatly advance the theoretical output of the review. The paper is a ‘key informant’

**MODERATE**: This category is for studies that show a ‘moderately’ relevant framing of the primary research to the review theories. This may mean that the article reports on a different (but related) intervention working toward similar outcomes of interest or describes middle-range theories that may inform the review even if there is no relevant empirical data from the paper to populate the CMO configurations or has a few areas that are of interest even if it is not entirely clear whether they will be used in the synthesis.

**LOW**: This category is for research that has met the selection criteria in terms of relevance to the review questions and the initial programme theories (or MRT) but is relatively thin on the description of context and mechanism. It is not placed in the exclusion category because it contains at least one idea or statement about the context, about the mechanisms or about conceptualizing outcomes that can be used for refining the theory and building a CMO configuration.


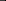


**EXCLUDE**: This category is for a research paper that showed promise on reading the citation, but upon reading the full-text paper does not correspond to the review questions, does not have any content that corresponds to the initial programme theories (or MRT), or does not describe at all the context, or the mechanisms (or process).

| **Record Number:** 10 |
| --- |
| **Data extraction by:** MNM  **Data extraction date:** 16 March 2024 |
| **Title**: Building Mental Health Research Capacity in Kenya: A South-North Collaboration |
| **First author/ year**: Mathai et al. (2019) |
| **List companion papers (multiple papers of the same study incl. grey literature)**  None |
| **Relevance and usefulness of this study (see end of the form for definitions)**  High [ ] Moderate [✓] Low [ ] None [ ] |
| **Summary of the Paper**  **(What is this about? What kind of data source? Quant, Qual, MM, Blog, etc.**  Methodology not described. The article is a process review and documentation of research project outcomes by the project team. |
| **What is interesting about this paper?**  The paper describes relevant C, M and O elements that are useful in the theory gleaning process. |
| **In what ways is this article relevant to the candidate programme theories, if at all? (Include specific pages, paragraph, line)**  **Context**   1. Ownership/support by institutional leadership (p. 180). 2. Limited resources to support research activities, heavy teaching responsibilities, clinical duties, 3. Administrative demands on senior faculty, and stigmatization of mental health conditions, treatment, and research within national society.   **Mechanism**   1. University faculty and postgraduate trainees *committed* to mental health research   **Outcomes**   1. Published papers and presentations at national and international meetings 2. Multidisciplinary research proposal written, submitted, and additional funding secured 3. A mental health research resource centre established 4. Established thematic research groups 5. One university faculty (beneficiary) took on a research leadership role on [x] donor-funded project |
| **What are the strengths and weaknesses of the article? Any red article?**  Methodology not described. Although the paper describes a few and fragmented C, M and O elements (without illustrating the link between the elements), the CMO components are relevant to research capacity strengthening in the context of an African university and therefore useful to the theory gleaning process. |
| **Describe any unintended positive or negative impacts and their mechanism link to the outcomes**  Ripple effect of the initiative: the established research centre/ hub accessible to wider population (beyond the intended university faculties and postgraduate trainees). |

**Definition of the categories**

The following definitions are only examples. These should be modified according to the needs of the study.

**HIGH**: This category is for papers that have high relevance to the realist synthesis. This means that the framing of the research and, the research questions are highly matched to the review questions, the empirical findings are clearly described and there is a rich description of the process and context that can greatly advance the theoretical output of the review. The paper is a ‘key informant’

**MODERATE**: This category is for studies that show a ‘moderately’ relevant framing of the primary research to the review theories. This may mean that the article reports on a different (but related) intervention working toward similar outcomes of interest or describes middle-range theories that may inform the review even if there is no relevant empirical data from the paper to populate the CMO configurations or has a few areas that are of interest even if it is not entirely clear whether they will be used in the synthesis.

**LOW**: This category is for research that has met the selection criteria in terms of relevance to the review questions and the initial programme theories (or MRT) but is relatively thin on the description of context and mechanism. It is not placed in the exclusion category because it contains at least one idea or statement about the context, about the mechanisms or about conceptualizing outcomes that can be used for refining the theory and building a CMO configuration.


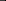


**EXCLUDE**: This category is for a research paper that showed promise on reading the citation, but upon reading the full-text paper does not correspond to the review questions, does not have any content that corresponds to the initial programme theories (or MRT), or does not describe at all the context, or the mechanisms (or process).

| **Record Number:** 11 |
| --- |
| **Data extraction by:** MNM  **Data extraction date:** 18 March 2024 |
| **Title**: A framework for sustainable capacity‑building for collaborative North–South translational health research and training in a resource‑constrained setting |
| **First author/ year**: Maponga et al. (2023) |
| **List companion papers (multiple papers of the same study incl. grey literature)**  None |
| **Relevance and usefulness of this study (see end of the form for definitions)**  High [✓] Moderate [ ] Low [ ] None [ ] |
| **Summary of the Paper**  **(What is this about? What kind of data source? Quant, Qual, MM, Blog, etc.**  No description of methods or data sources. The authors simply describe the initiative and how it was aimed at strengthening the capacity of the university. |
| **What is interesting about this paper?**  The authors describe a capacity-building programme between an African university and US-based university. The programme was operationalised around a mnemonic acronym, “RSTUVW”: Room (space), Skills, Tools (equipment)” underpinned by a set of core values, Understanding, Voice (clout), and Will (p. 4) |
| **In what ways is this article relevant to the candidate programme theories, if at all? (Include specific pages, paragraph, line)**  **Context**   1. Collaboration to support scientific and technological activities in resource-constrained countries 2. Support by the public sector - national research bodies 3. Lack of scientific research infrastructure 4. Inadequacies in skills (at individual levels) 5. Lack of dedicated and sustainable funding for research therefore heavily undercuts both operational feasibility and human motivation   **Mechanism**   1. Understanding (researchers’ understanding of the rationale for the research, awareness of their role in shaping policy and understanding of local priorities amongst the collaborators from the North) – workshops with actors can promote their understanding 2. Voice (individuals with the know-how to navigate the local political and technical context and champions from the North can advocate for the sustenance of the research collaboration) – researchers can seek the support and endorsement of their work to the highest authorities with clout 3. Will (willingness of the technical and policy actors can help to unlock budgetary allocation and other enabling support for the programme and long-term institutional commitments) – stakeholder engagements   **Outcomes**   1. Strengthened university faculty scholarly capacity (trained and mentored fellows have progressed to occupy faculty positions at local universities) 2. Technology and skills transfer to support research and practice 3. Evidence base for practice and policy strengthened 4. Research equipment and infrastructure established and strengthened (funding enabled procurement of tools such as laboratory equipment and reagents) |
| **What are the strengths and weaknesses of the article? Any red article?**  The article describes relevant and useful contextual conditions, mechanism and outcomes related to university’s research capacity strengthening, as core values, that are requisite for generating the desired capacity outcomes. Although the paper describes a few and fragmented C, M and O elements (without illustrating the link between the elements), the CMO components are relevant to research capacity strengthening in the context of an African university and therefore useful to the theory gleaning process. |
| **Describe any unintended positive or negative impacts and their mechanism link to the outcomes**  None |

**Definition of the categories**

The following definitions are only examples. These should be modified according to the needs of the study.

**HIGH**: This category is for papers that have high relevance to the realist synthesis. This means that the framing of the research and, the research questions are highly matched to the review questions, the empirical findings are clearly described and there is a rich description of the process and context that can greatly advance the theoretical output of the review. The paper is a ‘key informant’

**MODERATE**: This category is for studies that show a ‘moderately’ relevant framing of the primary research to the review theories. This may mean that the article reports on a different (but related) intervention working toward similar outcomes of interest or describes middle-range theories that may inform the review even if there is no relevant empirical data from the paper to populate the CMO configurations or has a few areas that are of interest even if it is not entirely clear whether they will be used in the synthesis.

**LOW**: This category is for research that has met the selection criteria in terms of relevance to the review questions and the initial programme theories (or MRT) but is relatively thin on the description of context and mechanism. It is not placed in the exclusion category because it contains at least one idea or statement about the context, about the mechanisms or about conceptualizing outcomes that can be used for refining the theory and building a CMO configuration.


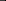


**EXCLUDE**: This category is for a research paper that showed promise on reading the citation, but upon reading the full-text paper does not correspond to the review questions, does not have any content that corresponds to the initial programme theories (or MRT), or does not describe at all the context, or the mechanisms (or process).

| **Record Number:** 12 |
| --- |
| **Data extraction by:** MNM  **Data extraction date:** 19 March 2024 |
| **Title**: Research capacity-building in Africa: networks, institutions and local ownership |
| **First author/ year**: Marjanovic et al. (2013) |
| **List companion papers (multiple papers of the same study incl. grey literature)**  None |
| **Relevance and usefulness of this study (see end of the form for definitions)**  High [ ] Moderate [✓] Low [ ] None [ ] |
| **Summary of the Paper**  **(What is this about? What kind of data source? Quant, Qual, MM, Blog, etc.**  The paper is based on the initial findings from an independent real-time evaluation of the initiative—i.e. an evaluation taking place during the programme’s life. |
| **What is interesting about this paper?**  Through reported evaluation evidence, the paper draws directly on the experiences of those who are implementing research capacity-building efforts: African research leaders, researchers, research managers, administrators, and collaborators in developed countries. Although the paper describes a few and fragmented C and M elements (without illustrating the link between the elements), the C and M components are relevant to research capacity strengthening in the context of an African university and therefore useful to the theory gleaning process. |
| **In what ways is this article relevant to the candidate programme theories, if at all? (Include specific pages, paragraph, line)**  **Context**   - Engaging high-level university leadership to create sustainable research career pathways - Support and buy-in of vice-chancellors and deans of faculties to establish memoranda of understanding and collaboration agreements - Research governance structures and policies in universities   **Mechanism**   - Sustaining commitment, enthusiasm, and delivery by all partners in the network |
| **What are the strengths and weaknesses of the article? Any red article?**  Authors argue that the programme is not mature for outcomes to be observed. The paper, however, provided useful insights that could inform the theory gleaning process i.e., research institutes have more established research management and governance infrastructures than universities; lead institutions tend to have more robust governance and management infrastructures than sub-awardees—both in terms of formal structures, systems, and research policies, and in terms of human resource support and institutionalisation of research management and administration functions (establishment of research support units) to help with recovering costs through future successful grant applications, to be self-financing and sustainable in the long term. |
| **Describe any unintended positive or negative impacts and their mechanism link to the outcomes**  None |

**Definition of the categories**

The following definitions are only examples. These should be modified according to the needs of the study.

**HIGH**: This category is for papers that have high relevance to the realist synthesis. This means that the framing of the research and, the research questions are highly matched to the review questions, the empirical findings are clearly described and there is a rich description of the process and context that can greatly advance the theoretical output of the review. The paper is a ‘key informant’

**MODERATE**: This category is for studies that show a ‘moderately’ relevant framing of the primary research to the review theories. This may mean that the article reports on a different (but related) intervention working toward similar outcomes of interest or describes middle-range theories that may inform the review even if there is no relevant empirical data from the paper to populate the CMO configurations or has a few areas that are of interest even if it is not entirely clear whether they will be used in the synthesis.

**LOW**: This category is for research that has met the selection criteria in terms of relevance to the review questions and the initial programme theories (or MRT) but is relatively thin on the description of context and mechanism. It is not placed in the exclusion category because it contains at least one idea or statement about the context, about the mechanisms or about conceptualizing outcomes that can be used for refining the theory and building a CMO configuration.


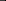


**EXCLUDE**: This category is for a research paper that showed promise on reading the citation, but upon reading the full-text paper does not correspond to the review questions, does not have any content that corresponds to the initial programme theories (or MRT), or does not describe at all the context, or the mechanisms (or process).

| **Record Number:** 13 |
| --- |
| **Data extraction by:** MNM  **Data extraction date:** 20 March 2024 |
| **Title**: HIV Research Training Partnership of the University of Zambia and Vanderbilt University: Features and Early Outcomes |
| **First author/ year**: Mutale et al. (2019) |
| **List companion papers (multiple papers of the same study incl. grey literature)**  None |
| **Relevance and usefulness of this study (see end of the form for definitions)**  High [✓] Moderate [ ] Low [ ] None [ ] |
| **Summary of the Paper**  **(What is this about? What kind of data source? Quant, Qual, MM, Blog, etc.**  Qualitative approach was used to report activities and lessons learned. Reports were limited to descriptions of an activity, output, or outcome but not impact. Some benefi­ciaries of the programme were interviewed to get their perspective and experi­ences on the programme. The authors reviewed the programme data and conducted interviews with program leaders and participants to understand and document the progress and outcomes of the partnership. The paper reports the programme’s early achievements, highlighting drivers and challenges. |
| **What is interesting about this paper?**  Although the paper describes a few and fragmented C, M and O elements (without illustrating the link between the elements), the CMO components are relevant to research capacity strengthening in the context of an African university and therefore useful to the theory gleaning process. |
| **In what ways is this article relevant to the candidate programme theories, if at all? (Include specific pages, paragraph, line)**  **Context**   1. Insti­tutional ownership fostered by aligning goals between the two partner universities 2. University priority driven and locally owned (leading the local health research agenda) 3. Long-term and sustained collaborations between the two partner universities 4. Protected time for faculty members to focus primarily on research   **Mechanism**   1. Research collaborations built on trust and balanced benefits. 2. The university leaders and faculty members were proactive in expressing the need and interest in reviewing the PhD programme and institutionalising mentorship of trainees 3. Workshops, training and mentorships are reportedly ‘eye opening’ for the faculty/PhD trainees as they gain new skills, network and get exposed to new opportunities   **Outcomes**   1. University PhD programme reviewed and revised 2. PhD Programme Director and Steering Committee established to improve the programme’s administrative processes. 3. Mentorship in the PhD programme institutionalised with a framework in place 4. New col­laborative relationships, grants, and publica­tions record following university’s two faculty staff taking research sabbaticals (pro­tected time) to focus on research |
| **What are the strengths and weaknesses of the article? Any red article?**  Although the paper describes a few and fragmented C and M elements (without illustrating the link between the elements), the C and M components are relevant to research capacity strengthening in the context of an African university and therefore useful to the theory gleaning process. |
| **Describe any unintended positive or negative impacts and their mechanism link to the outcomes**  None |

**Definition of the categories**

The following definitions are only examples. These should be modified according to the needs of the study.

**HIGH**: This category is for papers that have high relevance to the realist synthesis. This means that the framing of the research and, the research questions are highly matched to the review questions, the empirical findings are clearly described and there is a rich description of the process and context that can greatly advance the theoretical output of the review. The paper is a ‘key informant’

**MODERATE**: This category is for studies that show a ‘moderately’ relevant framing of the primary research to the review theories. This may mean that the article reports on a different (but related) intervention working toward similar outcomes of interest or describes middle-range theories that may inform the review even if there is no relevant empirical data from the paper to populate the CMO configurations or has a few areas that are of interest even if it is not entirely clear whether they will be used in the synthesis.

**LOW**: This category is for research that has met the selection criteria in terms of relevance to the review questions and the initial programme theories (or MRT) but is relatively thin on the description of context and mechanism. It is not placed in the exclusion category because it contains at least one idea or statement about the context, about the mechanisms or about conceptualizing outcomes that can be used for refining the theory and building a CMO configuration.


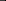


**EXCLUDE**: This category is for a research paper that showed promise on reading the citation, but upon reading the full-text paper does not correspond to the review questions, does not have any content that corresponds to the initial programme theories (or MRT), or does not describe at all the context, or the mechanisms (or process).

| **Record Number:** 14 |
| --- |
| **Data extraction by:** MNM  **Data extraction date:** 21 March 2024 |
| **Title**: Strengthening research management and support services in sub-Saharan African universities and research institutions |
| **First author/ year**: Pulford et al. (2020) |
| **List companion papers (multiple papers of the same study incl. grey literature)**  None |
| **Relevance and usefulness of this study (see end of the form for definitions)**  High [ ] Moderate [✓] Low [ ] None [ ] |
| **Summary of the Paper**  **(What is this about? What kind of data source? Quant, Qual, MM, Blog, etc.**  The paper employed qualitative methods (semi-structured interviews). The paper presents a synthesis of 28 RMS capacity assessments completed in 25 universities/research institutions from across 15 SSA countries between 2014 and 2018. All 28 capacity assessments were completed following a standardised methodology consisting of semi-structured interviews conducted with research and research support staff at the respective institution as well as document reviews and observation of onsite facilities. Data were extracted from the 28 reports detailing the findings of each assessment according to a framework synthesis approach – 13 distinct capacity gap categories emerged from across the 28 RMS capacity assessment reports. |
| **What is interesting about this paper?**  Although the paper describes a few contextual conditions (without illustrating the link between the elements), the contextual factors are relevant to research capacity strengthening in the context of an African university and therefore useful to the theory gleaning process. |
| **In what ways is this article relevant to the candidate programme theories, if at all? (Include specific pages, paragraph, line)**  **Context (relevant to both individual and institutional capacity levels)**   - Complex bureaucracy of the institutional operating environment - Physical research infrastructure and resources - Fiscal constraints (limited research funding) - Insufficient workforce and unmanageable workload - Uncompetitive and/or insufficient salary (remuneration) - Limited professional development activities for research and research support staff - Limited career pathways and progression - Limited research support and management - Limited internal (inter-departmental) communication and collaboration - Limited external communication and networking |
| **What are the strengths and weaknesses of the article? Any red article?**  The paper only focuses on contextual elements. The authors concluded that no single intervention type, or focus, would be sufficient to strengthen capacity across different areas of capacity gaps and therefore a combination of interventions, consisting of differing levels of cost and complexity, variously led (or supported) by both internal and external actors would be required to improve RMS capacity within universities. Interventions that address (even in part) fiscal constraints and complex bureaucracies may be especially impactful given the centrality of these issues across many of the 13 categories reported here. Determining which combination of interventions may be most appropriate for any one institution should be a collaborative process, engaging both research and research support staff (from senior to junior levels) from the focal institution and ideally delivered as part of a longer-term, overarching research capacity strengthening strategic plan (p. 5). |
| **Describe any unintended positive or negative impacts and their mechanism link to the outcomes**  None |

**Definition of the categories**

The following definitions are only examples. These should be modified according to the needs of the study.

**HIGH**: This category is for papers that have high relevance to the realist synthesis. This means that the framing of the research and, the research questions are highly matched to the review questions, the empirical findings are clearly described and there is a rich description of the process and context that can greatly advance the theoretical output of the review. The paper is a ‘key informant’

**MODERATE**: This category is for studies that show a ‘moderately’ relevant framing of the primary research to the review theories. This may mean that the article reports on a different (but related) intervention working toward similar outcomes of interest or describes middle-range theories that may inform the review even if there is no relevant empirical data from the paper to populate the CMO configurations or has a few areas that are of interest even if it is not entirely clear whether they will be used in the synthesis.

**LOW**: This category is for research that has met the selection criteria in terms of relevance to the review questions and the initial programme theories (or MRT) but is relatively thin on the description of context and mechanism. It is not placed in the exclusion category because it contains at least one idea or statement about the context, about the mechanisms or about conceptualizing outcomes that can be used for refining the theory and building a CMO configuration.


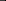


**EXCLUDE**: This category is for a research paper that showed promise on reading the citation, but upon reading the full-text paper does not correspond to the review questions, does not have any content that corresponds to the initial programme theories (or MRT), or does not describe at all the context, or the mechanisms (or process).

| **Record Number:** 15 |
| --- |
| **Data extraction by:** MNM  **Data extraction date:** 22 March 2024 |
| **Title**: “We need more big trees as well as the grass roots”: going beyond research capacity building to develop sustainable careers in mental health research in African countries |
| **First author/ year**: Langhaug et al. (2020) |
| **List companion papers (multiple papers of the same study incl. grey literature)**  None |
| **Relevance and usefulness of this study (see end of the form for definitions)**  High [ ] Moderate [✓] Low [ ] None [ ] |
| **Summary of the Paper**  **(What is this about? What kind of data source? Quant, Qual, MM, Blog, etc.**  Qualitative methods employed by the study. The authors conducted 52 qualitative interviews with early-career researchers, policymakers, academics, and service users from four African countries and with international funders of health research. [Methodology section: 46 interviews were conducted with academic leaders, policymakers, clinicians, research fellows, and service users from across the four countries. |
| **What is interesting about this paper?**  Although the paper describes a few and fragmented C and M elements (without illustrating the link between the elements), the C and M components are relevant to research capacity strengthening in the context of an African university and therefore useful to the theory gleaning process. |
| **In what ways is this article relevant to the candidate programme theories, if at all? (Include specific pages, paragraph, line)**  **Context**   - Lack of clear career pathways/tracks within universities for research (academic career tracks were limited to teaching and mentoring students) - Lack of infrastructure that enables high-quality research e.g., grants management and administration, university leadership and research culture - Lack of protected research time – researchers spend more time in administrative work rather than research - Research careers in government would increase government’s ability to use research but researchers end up doing administrative work - Scarcity of senior researchers to be mentors, and the extraordinary burden of being a senior researcher - Value of networking, which leads to greater exposure to senior researchers who are potential mentors   **Mechanism**   - University must recognise the importance of a balance between teaching and research. - Need for proactivity by in-country governments to fund and support universities - Involvement of policy makers at the outset of the research development - Both the government and the university levels in-country, are *interested* in providing that pathway, and working on developing that pathway for the researchers |
| **What are the strengths and weaknesses of the article? Any red article?**  Inconsistency in the number of interviews conducted. The abstract mentions 52 qualitative interviews with early-career researchers, policymakers, academics, and service users conducted across the four African countries and with international funders of health research. The article methodology section mentions 46 interviews conducted with academic leaders, policymakers, clinicians, research fellows, and service users from across the four countries. The paper primarily focused on how research is funded, research careers, the relationship between academia and government, and challenges that early-career researchers face thus providing insights on contextual realities that universities (and research institutions) face. |
| **Describe any unintended positive or negative impacts and their mechanism link to the outcomes**  None |

**Definition of the categories**

The following definitions are only examples. These should be modified according to the needs of the study.

**HIGH**: This category is for papers that have high relevance to the realist synthesis. This means that the framing of the research and, the research questions are highly matched to the review questions, the empirical findings are clearly described and there is a rich description of the process and context that can greatly advance the theoretical output of the review. The paper is a ‘key informant’

**MODERATE**: This category is for studies that show a ‘moderately’ relevant framing of the primary research to the review theories. This may mean that the article reports on a different (but related) intervention working toward similar outcomes of interest or describes middle-range theories that may inform the review even if there is no relevant empirical data from the paper to populate the CMO configurations or has a few areas that are of interest even if it is not entirely clear whether they will be used in the synthesis.

**LOW**: This category is for research that has met the selection criteria in terms of relevance to the review questions and the initial programme theories (or MRT) but is relatively thin on the description of context and mechanism. It is not placed in the exclusion category because it contains at least one idea or statement about the context, about the mechanisms or about conceptualizing outcomes that can be used for refining the theory and building a CMO configuration.


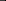


**EXCLUDE**: This category is for a research paper that showed promise on reading the citation, but upon reading the full-text paper does not correspond to the review questions, does not have any content that corresponds to the initial programme theories (or MRT), or does not describe at all the context, or the mechanisms (or process).

| **Record Number:** 16 |
| --- |
| **Data extraction by:** MNM  **Data extraction date:** 25 March 2024 |
| **Title**: Ripple effects of research capacity strengthening: a study of the effects of a project to support test facilities in three African countries towards Good Laboratory Practice certification |
| **First author/ year**: Begg et al. (2021) |
| **List companion papers (multiple papers of the same study incl. grey literature)**  None |
| **Relevance and usefulness of this study (see end of the form for definitions)**  High [ ] Moderate [✓] Low [ ] None [ ] |
| **Summary of the Paper**  **(What is this about? What kind of data source? Quant, Qual, MM, Blog, etc.**  A qualitative study that used a maximum-variation purposive sampling strategy. Data were triangulated between different sources. Semi-structured interviews were conducted with individuals in three facilities and a combination of email and remote video-call interviews were conducted with individuals at two further facilities. |
| **What is interesting about this paper?**  The paper focuses on a research capacity strengthening project supporting seven test facilities in Africa conducting studies on mosquito vector control products towards Good Laboratory Practice (GLP) certification. The study settings include 4 research institutions and 1 university contexts. |
| **In what ways is this article relevant to the candidate programme theories, if at all? (Include specific pages, paragraph, line)**  **Context**   - University-affiliated research facility - Unestablished research environment   **Outcomes**   - Development of GLP quality management system - Improved research infrastructure and resources - Development of more effective organisational structures - Research career pathways enhanced by strengthening the processes, policies, and documentation   **Mechanism**   - Clearer organisational structures facilitated communication between individuals in different departments |
| **What are the strengths and weaknesses of the article? Any red article?**  Whilst the project was focused on the institution level, important effects were identified at the individual level. These included extensive training, strengthening of career prospects, furtherment of careers, structured working practices and enhanced work motivation. Research capacity strengthening interventions that are focussed on institutional level require actions also at individual and national/international levels (p. 10)  Although the paper describes a few and fragmented C, M and O elements (without illustrating the link between the elements), the CMO components are relevant to research capacity strengthening in the context of an African university and therefore useful to the theory gleaning process. |
| **Describe any unintended positive or negative impacts and their mechanism link to the outcomes**  None |

**Definition of the categories**

The following definitions are only examples. These should be modified according to the needs of the study.

**HIGH**: This category is for papers that have high relevance to the realist synthesis. This means that the framing of the research and, the research questions are highly matched to the review questions, the empirical findings are clearly described and there is a rich description of the process and context that can greatly advance the theoretical output of the review. The paper is a ‘key informant’

**MODERATE**: This category is for studies that show a ‘moderately’ relevant framing of the primary research to the review theories. This may mean that the article reports on a different (but related) intervention working toward similar outcomes of interest or describes middle-range theories that may inform the review even if there is no relevant empirical data from the paper to populate the CMO configurations or has a few areas that are of interest even if it is not entirely clear whether they will be used in the synthesis.

**LOW**: This category is for research that has met the selection criteria in terms of relevance to the review questions and the initial programme theories (or MRT) but is relatively thin on the description of context and mechanism. It is not placed in the exclusion category because it contains at least one idea or statement about the context, about the mechanisms or about conceptualizing outcomes that can be used for refining the theory and building a CMO configuration.


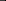


**EXCLUDE**: This category is for a research paper that showed promise on reading the citation, but upon reading the full-text paper does not correspond to the review questions, does not have any content that corresponds to the initial programme theories (or MRT), or does not describe at all the context, or the mechanisms (or process).

| **Record Number:** 17 |
| --- |
| **Data extraction by:** MNM  **Data extraction date:** 26 March 2024 |
| **Title**: Enabling research capacity strengthening within a consortium context: a qualitative study |
| **First author/ year**: Aiyenigba et al. (2022) |
| **List companion papers (multiple papers of the same study incl. grey literature)** |
| **Relevance and usefulness of this study (see end of the form for definitions)**  High [✓] Moderate [ ] Low [ ] None [ ] |
| **Summary of the Paper**  **(What is this about? What kind of data source? Quant, Qual, MM, Blog, etc.**  A qualitative study set within a health research capacity strengthening initiative. Semi-structured interviews were completed with 69 participants from seven institutions across six African countries belonging to three research consortia. Data were analysed thematically via a general inductive approach. Participants consisted of consortia funded masters, doctoral and postdoctoral fellows, academic faculty attached to the consortia, as well as consortium management, administrative and support staff. The academic faculty included academic supervisors, lecturers, departmental professors, and heads of department who had a role within the consortium, although were primarily employees of the respective member institutions. Participants were regionally located in East, West and Central Africa. |
| **What is interesting about this paper?**  The article describes how universities can be strengthened by being part of a research consortia. The primary research questions were: in what ways do consortium members perceive that they and their respective institutions’ research capacity is strengthened from said membership? And, drawing on member experiences, what are the common factors that enable these perceived gains in research capacity to be realised? |
| **In what ways is this article relevant to the candidate programme theories, if at all? (Include specific pages, paragraph, line)**  **Context**   - Access to funding - Leadership structures and practices (more engaged leaders of universities) – facilitated institutional-level RCS - Inclusive and engaging leadership - Access to funding is critical for realisation of the other three benefits.   **Mechanism**:   - University leaders have sustained institutional buy-in and support institutionalisation efforts - Interactions were powerful when consortium leadership were willing and able to engage with junior members on a more personal level   **Outcomes**   - Investment in infrastructure development including upgrading of facilities and procurement of specialist equipment - Access to funding and consortia resources including staff and training - Enhanced networking and research collaborations - Adoption of consortia-initiated ‘good practices’ and policies (eg, adoption of financial reporting templates or supportive childcare policies) - Enhanced reputation, through consortia membership and associated research impact - Better capacitated workforce - Expanded workforce—in administrative, professional, teaching and research roles - Career development opportunities for existing staff (eg, PhD fellows recruited from existing staff). |
| **What are the strengths and weaknesses of the article? Any red article?**  The authors examined how the programme was strengthening research capacities of the participating African institutions (including universities) and concluded that, besides direct infrastructural development, other institutional-level benefits of consortium membership were driven through investment in individuals. Although the paper describes a few and fragmented C, M and O elements (without illustrating the link between the elements), the CMO components are relevant to research capacity strengthening in the context of an African university and therefore useful to the theory gleaning process. |
| **Describe any unintended positive or negative impacts and their mechanism link to the outcomes**  Yes.  **Rival theory**  Available funding could also create inequities within member institutions between those colleagues who belonged to a consortium and those who did not. These inequities manifested in terms of both access to resources, for such things as training participation, research support and career supportive practices such as childcare provision to attend conferences, as well as distortions in academic salary scales |

**Definition of the categories**

The following definitions are only examples. These should be modified according to the needs of the study.

**HIGH**: This category is for papers that have high relevance to the realist synthesis. This means that the framing of the research and, the research questions are highly matched to the review questions, the empirical findings are clearly described and there is a rich description of the process and context that can greatly advance the theoretical output of the review. The paper is a ‘key informant’

**MODERATE**: This category is for studies that show a ‘moderately’ relevant framing of the primary research to the review theories. This may mean that the article reports on a different (but related) intervention working toward similar outcomes of interest or describes middle-range theories that may inform the review even if there is no relevant empirical data from the paper to populate the CMO configurations or has a few areas that are of interest even if it is not entirely clear whether they will be used in the synthesis.

**LOW**: This category is for research that has met the selection criteria in terms of relevance to the review questions and the initial programme theories (or MRT) but is relatively thin on the description of context and mechanism. It is not placed in the exclusion category because it contains at least one idea or statement about the context, about the mechanisms or about conceptualizing outcomes that can be used for refining the theory and building a CMO configuration.


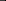


**EXCLUDE**: This category is for a research paper that showed promise on reading the citation, but upon reading the full-text paper does not correspond to the review questions, does not have any content that corresponds to the initial programme theories (or MRT), or does not describe at all the context, or the mechanisms (or process).

| **Record Number:** 18 |
| --- |
| **Data extraction by:** MNM  **Data extraction date:** 27 March 2024 |
| **Title**: Mentorship of young researchers in resource-limited settings: experiences of the mentees from selected health sciences Universities in Tanzania |
| **First author/ year**: Mremi et al. (2023) |
| **List companion papers (multiple papers of the same study incl. grey literature)**  None |
| **Relevance and usefulness of this study (see end of the form for definitions)**  High [ ] Moderate [✓] Low [ ] None [ ] |
| **Summary of the Paper**  **(What is this about? What kind of data source? Quant, Qual, MM, Blog, etc.**  A reflective paper describing the experiences of researcg mentees (junior faculty members who worked in local universities). The mentorship was delivered under a consortium of three health universities in East Africa in collaboration with US universities. |
| **What is interesting about this paper?**  Although the paper describes a few and fragmented C, M and O elements (without illustrating the link between the elements), the CMO components are relevant to research capacity strengthening in the context of an African university and therefore useful to the theory gleaning process. |
| **In what ways is this article relevant to the candidate programme theories, if at all? (Include specific pages, paragraph, line)**  **Context**   - Low resource settings - Few junior academic staff with PhD qualifications   **Mechanism**   - The mentors had the expertise in research methodology and/or data analysis - Mentors’ willingness to mentor junior faculty members - Mentees’ readiness for research career and need for mentorship support   **Outcomes**   - Mentees skills improved in key research areas e.g., grant and manuscript writing - Institutional staff with PhD qualifications increased - Research grants secured – increasing the institutional research profile |
| **What are the strengths and weaknesses of the article? Any red article?**  C, M and O elements reported by the paper. The disposition towards men­torship was established among the mentees, who men­tored junior faculty members as well as undergraduate students. These actions support the sustainability of men­torship practices in the three partnering institutions and other similar institutions where the mentees are likely to serve during their careers (p. 5) |
| **Describe any unintended positive or negative impacts and their mechanism link to the outcomes**  None |

**Definition of the categories**

The following definitions are only examples. These should be modified according to the needs of the study.

**HIGH**: This category is for papers that have high relevance to the realist synthesis. This means that the framing of the research and, the research questions are highly matched to the review questions, the empirical findings are clearly described and there is a rich description of the process and context that can greatly advance the theoretical output of the review. The paper is a ‘key informant’

**MODERATE**: This category is for studies that show a ‘moderately’ relevant framing of the primary research to the review theories. This may mean that the article reports on a different (but related) intervention working toward similar outcomes of interest or describes middle-range theories that may inform the review even if there is no relevant empirical data from the paper to populate the CMO configurations or has a few areas that are of interest even if it is not entirely clear whether they will be used in the synthesis.

**LOW**: This category is for research that has met the selection criteria in terms of relevance to the review questions and the initial programme theories (or MRT) but is relatively thin on the description of context and mechanism. It is not placed in the exclusion category because it contains at least one idea or statement about the context, about the mechanisms or about conceptualizing outcomes that can be used for refining the theory and building a CMO configuration.


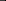


**EXCLUDE**: This category is for a research paper that showed promise on reading the citation, but upon reading the full-text paper does not correspond to the review questions, does not have any content that corresponds to the initial programme theories (or MRT), or does not describe at all the context, or the mechanisms (or process).

| **Record Number:** 19 |
| --- |
| **Data extraction by:** MNM  **Data extraction date:** 28 March 2024 |
| **Title**: Medical Education Partnership Initiative in Zimbabwe: partnerships for transformation |
| **First author/ year**: Hakim et al. (2017) |
| **List companion papers (multiple papers of the same study incl. grey literature)**  None |
| **Relevance and usefulness of this study (see end of the form for definitions)**  High [ ] Moderate [✓] Low [ ] None [ ] |
| **Summary of the Paper**  **(What is this about? What kind of data source? Quant, Qual, MM, Blog, etc.**  The correspondence paper presents the achievements of an African university under a health research capacity strengthening programme. |
| **What is interesting about this paper?**  The correspondence paper presents the institutional level HRCS outcomes of a research capacity strengthening programme and to some extent contextual elements that enabled or hindered the achievement of those outcomes. Although the paper describes a few and fragmented C and O elements (without illustrating the link between the elements), the C and O components are relevant to research capacity strengthening in the context of an African university and therefore useful to the theory gleaning process. |
| **In what ways is this article relevant to the candidate programme theories, if at all? (Include specific pages, paragraph, line)**  **Context**   - Political and economic crisis (1999 and 2010) which resulted to decrease in medical student enrolment, faculty vacancy rate, and increase in failure rate - Government buy-in and support   **Outcomes**   - Faculty development and research capacity strengthening have been institutionalised - New Research Support Centre established - Staff/ faculty retention increased, with the faculty growing by 36% (from 122 to 166 staff) - Synergistic and sustained collaboration/partnerships beyond programme lifecycle - Ripple effect –training junior faculty to become competent and independent high-level researchers. |
| **What are the strengths and weaknesses of the article? Any red article?**  The paper does not make the CMO connection but describes how specific RCS interventions lead to certain capacity outcomes at both individual and institutional levels. This evidence is useful in corroborating insights derived from other literature sources. |
| **Describe any unintended positive or negative impacts and their mechanism link to the outcomes**  None |

**Definition of the categories**

The following definitions are only examples. These should be modified according to the needs of the study.

**HIGH**: This category is for papers that have high relevance to the realist synthesis. This means that the framing of the research and, the research questions are highly matched to the review questions, the empirical findings are clearly described and there is a rich description of the process and context that can greatly advance the theoretical output of the review. The paper is a ‘key informant’

**MODERATE**: This category is for studies that show a ‘moderately’ relevant framing of the primary research to the review theories. This may mean that the article reports on a different (but related) intervention working toward similar outcomes of interest or describes middle-range theories that may inform the review even if there is no relevant empirical data from the paper to populate the CMO configurations or has a few areas that are of interest even if it is not entirely clear whether they will be used in the synthesis.

**LOW**: This category is for research that has met the selection criteria in terms of relevance to the review questions and the initial programme theories (or MRT) but is relatively thin on the description of context and mechanism. It is not placed in the exclusion category because it contains at least one idea or statement about the context, about the mechanisms or about conceptualizing outcomes that can be used for refining the theory and building a CMO configuration.


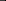


**EXCLUDE**: This category is for a research paper that showed promise on reading the citation, but upon reading the full-text paper does not correspond to the review questions, does not have any content that corresponds to the initial programme theories (or MRT), or does not describe at all the context, or the mechanisms (or process).

| **Record Number:** 20 |
| --- |
| **Data extraction by:** MNM  **Data extraction date:** 29 March 2024 |
| **Title**: Rebuilding research capacity in fragile states: the case of a Somali–Swedish global health initiative |
| **First author/ year**: Dalmar et al. (2017) |
| **List companion papers (multiple papers of the same study incl. grey literature)**  None |
| **Relevance and usefulness of this study (see end of the form for definitions)**  High [ ] Moderate [✓] Low [ ] None [ ] |
| **Summary of the Paper**  **(What is this about? What kind of data source? Quant, Qual, MM, Blog, etc.**  The reflection paper drawing qualitative evidence. |
| **What is interesting about this paper?**  The described programme focuses on research capacity building in the health sector currently underway through the work of an alliance of three partner groups: six new national universities, five Western universities, and diaspora professionals. Although the paper describes a few and fragmented C and O elements (without illustrating the link between the elements), the C and O components are relevant to research capacity strengthening in the context of an African university and therefore useful to the theory gleaning process. |
| **In what ways is this article relevant to the candidate programme theories, if at all? (Include specific pages, paragraph, line)**  **Context**   - Local ownership is key to the sustainability of the programme – ensuring buy-in by other local actors e.g., health ministries. - Political instability (how it affects HRCS initiatives)   **Outcomes**   - Research integrated as a key pillar of the academic programmes - Establishment of committees to deliberate on ethical issues - Number of university teachers with PhD degrees across the region increased - Inter-university collaboration between local and Global North universities strengthened - Research infrastructure strengthened |
| **What are the strengths and weaknesses of the article? Any red article?**  Mechanism not reported. However, the paper highlights political stability as a key contextual factor which can affect the RCS efforts and outcomes. Effective training for research goes hand in hand with doing research itself which depends on the availability of appropriate infrastructure in terms of physical assets such as offices and laboratories, computer facilities, as well as institutionalised administrative and regulatory functions. Involving domestic and external universities as well as diaspora academics, particularly in fragile, post-conflict contexts may prove to be a constructive model for international cooperation as the ivolved individuals already have a cultural familiarity. Long term partnerships/ collaborations needed to foster real systemic change in health research capacity in national universities (p. 7). |
| **Describe any unintended positive or negative impacts and their mechanism link to the outcomes**  None |

**Definition of the categories**

The following definitions are only examples. These should be modified according to the needs of the study.

**HIGH**: This category is for papers that have high relevance to the realist synthesis. This means that the framing of the research and, the research questions are highly matched to the review questions, the empirical findings are clearly described and there is a rich description of the process and context that can greatly advance the theoretical output of the review. The paper is a ‘key informant’

**MODERATE**: This category is for studies that show a ‘moderately’ relevant framing of the primary research to the review theories. This may mean that the article reports on a different (but related) intervention working toward similar outcomes of interest or describes middle-range theories that may inform the review even if there is no relevant empirical data from the paper to populate the CMO configurations or has a few areas that are of interest even if it is not entirely clear whether they will be used in the synthesis.

**LOW**: This category is for research that has met the selection criteria in terms of relevance to the review questions and the initial programme theories (or MRT) but is relatively thin on the description of context and mechanism. It is not placed in the exclusion category because it contains at least one idea or statement about the context, about the mechanisms or about conceptualizing outcomes that can be used for refining the theory and building a CMO configuration.


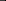


**EXCLUDE**: This category is for a research paper that showed promise on reading the citation, but upon reading the full-text paper does not correspond to the review questions, does not have any content that corresponds to the initial programme theories (or MRT), or does not describe at all the context, or the mechanisms (or process).

| **Record Number:** 21 |
| --- |
| **Data extraction by:** MNM  **Data extraction date:** 1 April 2024 |
| **Title**: “It takes more than a fellowship program”: reflections on capacity strengthening for health systems research in sub-Saharan Africa |
| **First author/ year**: **Izugbara** et al. (2017) |
| **List companion papers (multiple papers of the same study incl. grey literature)**  None |
| **Relevance and usefulness of this study (see end of the form for definitions)**  High [ ] Moderate [✓] Low [ ] None [ ] |
| **Summary of the Paper (What is this about? What kind of data source? Quant, Qual, MM, Blog, etc.**  A reflective paper utilising both quantitative and qualitative evidence. |
| **What is interesting about this paper?**  The paper describes the linkage between individual level capacities and institutional capacities – as individuals/ beneficiaries secured complementary funding. It demonstrates how individual capacities can be translated to institutional capacities (individual capacities can benefit both the individual and the university within which the individual is working). |
| **In what ways is this article relevant to the candidate programme theories, if at all? (Include specific pages, paragraph, line)**  **Context**   - Investments from local sources especially governments and the private sector in Africa. - Networks that can sustain post-docs as scholars and researchers (p.3).   **Outcomes**   - Post-docs emerging as research leaders and change agents in their institutions are supporting the transformations of their home-institutions into research-active hubs (p.3). - Most of these graduates remain active in research (or and teaching) at the university level – useful to the research profile of their universities - Post-docs’ fundraising efforts contributing to strengthening the institutional research capacity   **Mechanism**   - The fellows must be willing, ready, able, and supported to drive substantive and sustainable institutional change/transformation at African universities (p.4). |
| **What are the strengths and weaknesses of the article? Any red article?**  This is a reflective piece describing useful and relevant CMO components. Although the paper describes a few and fragmented C, M and O elements (without illustrating the link between the elements), the CMO components are relevant to research capacity strengthening in the context of an African university and therefore useful to the theory gleaning process. |
| **Describe any unintended positive or negative impacts and their mechanism link to the outcomes**  None |

**Definition of the categories**

The following definitions are only examples. These should be modified according to the needs of the study.

**HIGH**: This category is for papers that have high relevance to the realist synthesis. This means that the framing of the research and, the research questions are highly matched to the review questions, the empirical findings are clearly described and there is a rich description of the process and context that can greatly advance the theoretical output of the review. The paper is a ‘key informant’

**MODERATE**: This category is for studies that show a ‘moderately’ relevant framing of the primary research to the review theories. This may mean that the article reports on a different (but related) intervention working toward similar outcomes of interest or describes middle-range theories that may inform the review even if there is no relevant empirical data from the paper to populate the CMO configurations or has a few areas that are of interest even if it is not entirely clear whether they will be used in the synthesis.

**LOW**: This category is for research that has met the selection criteria in terms of relevance to the review questions and the initial programme theories (or MRT) but is relatively thin on the description of context and mechanism. It is not placed in the exclusion category because it contains at least one idea or statement about the context, about the mechanisms or about conceptualizing outcomes that can be used for refining the theory and building a CMO configuration.


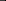


**EXCLUDE**: This category is for a research paper that showed promise on reading the citation, but upon reading the full-text paper does not correspond to the review questions, does not have any content that corresponds to the initial programme theories (or MRT), or does not describe at all the context, or the mechanisms (or process).

| **Record Number:** 22/23/24 |
| --- |
| **Data extraction by:** MNM  **Data extraction date:** 2 – 4 April 2024 |
| **Title**: The Medical Education Partnership Initiative Effect on Increasing Health Professions Education and Research Capacity in Mozambique |
| **First author/ year**: Noormahomed et al. (2018) |
| **List companion papers (multiple papers of the same study incl. grey literature)**  Two other papers describing the same programme were included.   - First paper described the contextual elements, programme resources (including programme architecture) and some of the anticipated/ emerging outcomes. The authors highlighted that at the time of the writing the paper, the programme had not matured enough to yield any substantive outcomes. - Second paper described the outcomes and, to some extent, mechanisms following a reflection on the programme implementation, successes, and challenges. |
| **Relevance and usefulness of this study (see end of the form for definitions)**  High [✓] Moderate [ ] Low [ ] None [ ] |
| **Summary of the Paper**  **(What is this about? What kind of data source? Quant, Qual, MM, Blog, etc.**  Reflective papers (2) and a qualitative study reporting on the same programme. |
| **What is interesting about this paper?**  The papers describe contexts, mechanism and outcomes following a reflection on the programme implementation, successes, and challenges. Although the paper describes a few and fragmented C, M and O elements (without illustrating the link between the elements), the CMO components are relevant to research capacity strengthening in the context of an African university and therefore useful to the theory gleaning process. |
| **In what ways is this article relevant to the candidate programme theories, if at all? (Include specific pages, paragraph, line)**  **Context**   - Strong leadership and local ownership facilitated the introduction of necessary change in local institutions - Limited university researchers committed full-time to research - Lack of well-equipped laboratories and research equipment - Lack of financial resources, grants administration and administrative management policies - Limited access to library resources (i.e., scientific literature) - Human research subject protection and ethical review   **Outcomes**   - Research support centre offered a mechanism to sustainably build on programme achievements - Physical infrastructure developed (interactive communication technologies enabled the scaling up of training and research and Laboratory infrastructure and equipment upgraded and training of the technical staff) - Training programs (e.g., in research methods, grant proposal and manuscript writing, and human subject protection for research) for­mally integrated into postgraduate training - Institutional Review Board (IRB) established to accelerate human subject research review - A multidisciplinary group of faculty developed and retained - Research proposals and funding secured (13 projects received external funding   **Mechanism**   - Ownership of the program was paramount to achiev­ing the results presented above. - Faculty members were motivated and empowered to put their research skills into practice and leverage each other’s capabilities - Research grants to faculty mem­bers provided incentives that resulted in reten­tion of staff - Donor’s flexibility allowed the teams to address local priorities and needs |
| **What are the strengths and weaknesses of the article? Any red article?**  C, M and O elements reported by the papers and useful in the theory gleaning process. Additional insights useful in the theorising process: Individual level capacity is translated into institutional capacities in two ways: a) institutional/ university staff and researchers trained in various skillsets (e.g., grant writing, publication, public and community engagement, etc) and those without PhDs supported to acquire the qualification and, b) formerly trained masters, PhD and postdoctoral trainees take up research and research management roles in the university (mostly reported as positive unintended outcomes). Learning by doing (trainees supported to strengthen their research capacities while embedded within a research programme) and trainees acquire essential research skills and have first-hand experience of how to carry out research and generate requisite research outputs. This is different from initiatives which primarily focus on capacity strengthening – for instance, scholarships only – for masters and PhD training. In the former, the student have the benefit of putting their newly acquired skills into practice and consequently strengthening those specific skillsets. |
| **Describe any unintended positive or negative impacts and their mechanism link to the outcomes**  None |

**Definition of the categories**

The following definitions are only examples. These should be modified according to the needs of the study.

**HIGH**: This category is for papers that have high relevance to the realist synthesis. This means that the framing of the research and, the research questions are highly matched to the review questions, the empirical findings are clearly described and there is a rich description of the process and context that can greatly advance the theoretical output of the review. The paper is a ‘key informant’

**MODERATE**: This category is for studies that show a ‘moderately’ relevant framing of the primary research to the review theories. This may mean that the article reports on a different (but related) intervention working toward similar outcomes of interest or describes middle-range theories that may inform the review even if there is no relevant empirical data from the paper to populate the CMO configurations or has a few areas that are of interest even if it is not entirely clear whether they will be used in the synthesis.

**LOW**: This category is for research that has met the selection criteria in terms of relevance to the review questions and the initial programme theories (or MRT) but is relatively thin on the description of context and mechanism. It is not placed in the exclusion category because it contains at least one idea or statement about the context, about the mechanisms or about conceptualizing outcomes that can be used for refining the theory and building a CMO configuration.


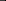


**EXCLUDE**: This category is for a research paper that showed promise on reading the citation, but upon reading the full-text paper does not correspond to the review questions, does not have any content that corresponds to the initial programme theories (or MRT), or does not describe at all the context, or the mechanisms (or process).

| **Record Number:** 25 |
| --- |
| **Data extraction by:** MNM  **Data extraction date:** 5 April 2024 |
| **Title**: African-led health research and capacity building- is it working? |
| **First author/ year**: Kasprowicz et al. 2020. |
| **List companion papers (multiple papers of the same study incl. grey literature)**  None |
| **Relevance and usefulness of this study (see end of the form for definitions)**  High [ ] Moderate [✓] Low [ ] None [ ] |
| **Summary of the Paper**  **(What is this about? What kind of data source? Quant, Qual, MM, Blog, etc.**  This is a reflective piece which does not describe methods. |
| **What is interesting about this paper?**  Delineation of contexts and outcomes in the context of university research capacity strengthening in Africa. Although the paper describes a few and fragmented C, M and O elements (without illustrating the link between the elements), the CMO components are relevant to research capacity strengthening in the context of an African university and therefore useful to the theory gleaning process. |
| **In what ways is this article relevant to the candidate programme theories, if at all? (Include specific pages, paragraph, line)**  **Context**   - Navigating university-level bureaucracy - Long-term investment from international donors and increasing funding commitments from African governments and philanthropies - Not enough research-protected time for faculty level researchers; too many trainees per supervisor; lack of funding schemes catering to the increased numbers of trainees as they progress in their careers; internet speed issues; lack of local degree training programmes at universities; academia viewed as an unrealistic career path; insufficient funding for research projects; and pipeline issues for high-quality trainee recruitment e.g. poor training at school and undergraduate level (p. 8).   **Outcomes**   - Enhanced local ownership of activities and new opportunities for steady and sustained skills building of staff and trainees - Improved research outputs including grants awarded to African researchers - Increased opportunities for senior African scientists to act as role models for junior scientists (e.g. to advise on how to successfully navigate university administrative systems) - Increased South-South collaborations - External collaboration, knowledge exchange and financial support |
| **What are the strengths and weaknesses of the article? Any red article?**  Mechanism not reported. The authors conclude that African-led and -funded research has the potential to overcome the vicious cycle of brain-drain and may ultimately lead to improvement of health and science-led economic transformation of Africa into a prosperous continent (p. 1) which is a critical insight in the theorising process. |
| **Describe any unintended positive or negative impacts and their mechanism link to the outcomes**  None |

**Definition of the categories**

The following definitions are only examples. These should be modified according to the needs of the study.

**HIGH**: This category is for papers that have high relevance to the realist synthesis. This means that the framing of the research and, the research questions are highly matched to the review questions, the empirical findings are clearly described and there is a rich description of the process and context that can greatly advance the theoretical output of the review. The paper is a ‘key informant’

**MODERATE**: This category is for studies that show a ‘moderately’ relevant framing of the primary research to the review theories. This may mean that the article reports on a different (but related) intervention working toward similar outcomes of interest or describes middle-range theories that may inform the review even if there is no relevant empirical data from the paper to populate the CMO configurations or has a few areas that are of interest even if it is not entirely clear whether they will be used in the synthesis.

**LOW**: This category is for research that has met the selection criteria in terms of relevance to the review questions and the initial programme theories (or MRT) but is relatively thin on the description of context and mechanism. It is not placed in the exclusion category because it contains at least one idea or statement about the context, about the mechanisms or about conceptualizing outcomes that can be used for refining the theory and building a CMO configuration.


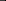


**EXCLUDE**: This category is for a research paper that showed promise on reading the citation, but upon reading the full-text paper does not correspond to the review questions, does not have any content that corresponds to the initial programme theories (or MRT), or does not describe at all the context, or the mechanisms (or process).

| **Record Number:** 26 |
| --- |
| **Data extraction by:** MNM  **Data extraction date:** 8 April 2024 |
| **Title**: Developing independent investigators for clinical research relevant for Africa |
| **First author/ year**: Manabe et al. (2011) |
| **List companion papers (multiple papers of the same study incl. grey literature)**  None |
| **Relevance and usefulness of this study (see end of the form for definitions)**  High [ ] Moderate [✓] Low [ ] None [ ] |
| **Summary of the Paper**  **(What is this about? What kind of data source? Quant, Qual, MM, Blog, etc.**  A commentary paper. The authors reflect on qualitative data. |
| **What is interesting about this paper?**  The authors examine the training at an African university as a model for in-country training on systems capacity building. Training the next generation of researchers within centres of excellence with strong institutional infrastructure will empower trainees to ask locally relevant research questions that will benefit Africans. The paper reports useful contexts, mechanism and outcomes related to research capacity strengthening in the context of African universities. |
| **In what ways is this article relevant to the candidate programme theories, if at all? (Include specific pages, paragraph, line)**  **Context**   1. A non-governmental organization, Centre of Excellence, owned by the University established 2. Mission of improving health through research, training, and clinical care 3. Multiple funding schemes and partners 4. Program has provided stipends for protected time for research, research funds as well as funding for administrative structures for accountability and mentoring. 5. Brain drain of scientists 6. Alignment with local and national health research policies and strategies   **Mechanism**   1. Trust between development partners 2. Local ownership. (Figure 2)   **Outcomes**   1. rigorous scientific and operational review of all proposals at the Institute with a panel of investigators from within and outside the IDI 2. strong grants management and financial accountability 3. annual committee appraisals with clear performance metrics 4. clinical research training unit that offers regular trainings in good clinical practice and regulatory compliance, a new DataFax electronic data management system for local and remote use, and regulatory coordination and internal monitoring 5. strong, internationally accredited, core laboratory in support of clinical research with freezer repository storage. |
| **What are the strengths and weaknesses of the article? Any red article?**  The paper does not make a clear linkage between context, mechanism, and outcomes. Although the paper describes a few and fragmented C, M and O elements (without illustrating the link between the elements), the CMO components are relevant to research capacity strengthening in the context of an African university and therefore useful to the theory gleaning process. |
| **Describe any unintended positive or negative impacts and their mechanism link to the outcomes**  None |

**Definition of the categories**

The following definitions are only examples. These should be modified according to the needs of the study.

**HIGH**: This category is for papers that have high relevance to the realist synthesis. This means that the framing of the research and, the research questions are highly matched to the review questions, the empirical findings are clearly described and there is a rich description of the process and context that can greatly advance the theoretical output of the review. The paper is a ‘key informant’

**MODERATE**: This category is for studies that show a ‘moderately’ relevant framing of the primary research to the review theories. This may mean that the article reports on a different (but related) intervention working toward similar outcomes of interest or describes middle-range theories that may inform the review even if there is no relevant empirical data from the paper to populate the CMO configurations or has a few areas that are of interest even if it is not entirely clear whether they will be used in the synthesis.

**LOW**: This category is for research that has met the selection criteria in terms of relevance to the review questions and the initial programme theories (or MRT) but is relatively thin on the description of context and mechanism. It is not placed in the exclusion category because it contains at least one idea or statement about the context, about the mechanisms or about conceptualizing outcomes that can be used for refining the theory and building a CMO configuration.


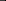


**EXCLUDE**: This category is for a research paper that showed promise on reading the citation, but upon reading the full-text paper does not correspond to the review questions, does not have any content that corresponds to the initial programme theories (or MRT), or does not describe at all the context, or the mechanisms (or process).

| **Record Number:** 27 |
| --- |
| **Data extraction by:** MNM  **Data extraction date:** 9 April 2024 |
| **Title**: Strengthening Research Governance for Sustainable Research: Experiences from Three Zimbabwean Universities |
| **First author/ year**: Mashaah et al. (2014) |
| **List companion papers (multiple papers of the same study incl. grey literature)**  None |
| **Relevance and usefulness of this study (see end of the form for definitions)**  High [✓] Moderate [ ] Low [ ] None [ ] |
| **Summary of the Paper**  **(What is this about? What kind of data source? Quant, Qual, MM, Blog, etc.**  No description of the methodology or study design. |
| **What is interesting about this paper?**  The authors describe the approach, strategies, processes, outputs, and outcomes of strengthening research governance across three universities in an African country and discusses the challenges of doing so. These topical issues provide insights related to context, mechanisma nd outcomes needed for the theory gleaning process. |
| **In what ways is this article relevant to the candidate programme theories, if at all? (Include specific pages, paragraph, line)**  **Context**   1. Three universities with unique research environments 2. Participation of the university management and the academic and research leadership [context] 3. Inadequate resources to sustain the structures and systems for governance and management 4. Instruction-oriented universities thus minimal financial and policy support for research 5. Lack of research governance and management policy   **Mechanism**   1. Obtaining *buy-in* from university management and faculty whose main emphasis was on teaching and who generally feared increased bureaucracy. 2. Management was also *wary* of the financial implications of the research governance initiative because the requirement for establishing research management structures, including funding, was embedded in the policy 3. Participatory approach to the review of current research governance status, establishment of a Technical Working group (TWG) to draft generic policy, and institutional-level consultations to adapt the policy. 4. TWG members held consultative workshops to adopt the policies [mechanism?]   **Outcomes**   1. Research governance framework developed and adopted (including an over-arching research policy and grants management system and procedures) in each university 2. Research management offices at the universities centralised and resourced (including furniture, computers, and supplies) |
| **What are the strengths and weaknesses of the article? Any red article?**  The authors do not describe the source of data or how the data is curated. However, useful and relevant insights are presented.  Although the paper describes a few and fragmented C, M and O elements (without illustrating the link between the elements), the CMO components are relevant to research capacity strengthening in the context of an African university and therefore useful to the theory gleaning process. |
| **Describe any unintended positive or negative impacts and their mechanism link to the outcomes**  None |

**Definition of the categories**

The following definitions are only examples. These should be modified according to the needs of the study.

**HIGH**: This category is for papers that have high relevance to the realist synthesis. This means that the framing of the research and, the research questions are highly matched to the review questions, the empirical findings are clearly described and there is a rich description of the process and context that can greatly advance the theoretical output of the review. The paper is a ‘key informant’

**MODERATE**: This category is for studies that show a ‘moderately’ relevant framing of the primary research to the review theories. This may mean that the article reports on a different (but related) intervention working toward similar outcomes of interest or describes middle-range theories that may inform the review even if there is no relevant empirical data from the paper to populate the CMO configurations or has a few areas that are of interest even if it is not entirely clear whether they will be used in the synthesis.

**LOW**: This category is for research that has met the selection criteria in terms of relevance to the review questions and the initial programme theories (or MRT) but is relatively thin on the description of context and mechanism. It is not placed in the exclusion category because it contains at least one idea or statement about the context, about the mechanisms or about conceptualizing outcomes that can be used for refining the theory and building a CMO configuration.


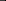


**EXCLUDE**: This category is for a research paper that showed promise on reading the citation, but upon reading the full-text paper does not correspond to the review questions, does not have any content that corresponds to the initial programme theories (or MRT), or does not describe at all the context, or the mechanisms (or process).

| **Record Number:** 28 |
| --- |
| **Data extraction by:** MNM  **Data extraction date:** 10 April 2024 |
| **Title**: Mbarara University Research Training Initiative: Experiences and Accomplishments of the MEPI Junior D43 TW010128 Award in Uganda |
| **First author/ year**: Wakida et al. (2021) |
| **List companion papers (multiple papers of the same study incl. grey literature)**  None |
| **Relevance and usefulness of this study (see end of the form for definitions)**  High [ ] Moderate [✓] Low [ ] None [ ] |
| **Summary of the Paper**  **(What is this about? What kind of data source? Quant, Qual, MM, Blog, etc.**  The paper uses primary and secondary data sources. The primary component utilises qualitative methods (indepth interviews). |
| **What is interesting about this paper?**  The paper describes context and outcomes of a research capacity strengthening iintiative in an African university. It specifically discusses the effects of a mentorship scheme targeting junior faculty in an African university. |
| **In what ways is this article relevant to the candidate programme theories, if at all? (Include specific pages, paragraph, line)**  **Outcomes**   - Extramural grants awarded (14 extramural) to the former trainees (2) or indirectly through the investigators, key personnel, or faculty (12). - 48 Publications directly attributed to the grant and 6 spin-off publications raised the institutional profile   **Context**   - Mentor–mentee relationship, teamwork, and leadership provided by the program directors. - Adequate implementation policies and procedures |
| **What are the strengths and weaknesses of the article? Any red article?**  There were data triangulation between sources including through document review and in-depth interviews with junior faculty trainees who benefited from the programme thus strengthening the evidence. Although the paper describes a few and fragmented C, M and O elements (without illustrating the link between the elements), the CMO components are relevant to research capacity strengthening in the context of an African university and therefore useful to the theory gleaning process. |
| **Describe any unintended positive or negative impacts and their mechanism link to the outcomes**  None |

**Definition of the categories**

The following definitions are only examples. These should be modified according to the needs of the study.

**HIGH**: This category is for papers that have high relevance to the realist synthesis. This means that the framing of the research and, the research questions are highly matched to the review questions, the empirical findings are clearly described and there is a rich description of the process and context that can greatly advance the theoretical output of the review. The paper is a ‘key informant’

**MODERATE**: This category is for studies that show a ‘moderately’ relevant framing of the primary research to the review theories. This may mean that the article reports on a different (but related) intervention working toward similar outcomes of interest or describes middle-range theories that may inform the review even if there is no relevant empirical data from the paper to populate the CMO configurations or has a few areas that are of interest even if it is not entirely clear whether they will be used in the synthesis.

**LOW**: This category is for research that has met the selection criteria in terms of relevance to the review questions and the initial programme theories (or MRT) but is relatively thin on the description of context and mechanism. It is not placed in the exclusion category because it contains at least one idea or statement about the context, about the mechanisms or about conceptualizing outcomes that can be used for refining the theory and building a CMO configuration.


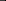


**EXCLUDE**: This category is for a research paper that showed promise on reading the citation, but upon reading the full-text paper does not correspond to the review questions, does not have any content that corresponds to the initial programme theories (or MRT), or does not describe at all the context, or the mechanisms (or process).

| **Record Number:** 29 |
| --- |
| **Data extraction by:** MNM  **Data extraction date:** 11 April 2024 |
| **Title**: Development of a South-South Clinical Research Capacity Strengthening: Institutional Partnership Platform between Cameroon and South Africa |
| **First author/ year**: **Ikomey** et al. (2023) |
| **List companion papers (multiple papers of the same study incl. grey literature)**  None |
| **Relevance and usefulness of this study (see end of the form for definitions)**  High [✓] Moderate [ ] Low [ ] None [ ] |
| **Summary of the Paper**  **(What is this about? What kind of data source? Quant, Qual, MM, Blog, etc.**  The paper employs qualitative methods. The observational study was conducted from 2016- 2022 of the collaboration between two African universities. |
| **What is interesting about this paper?**  An observational analysis was conducted from 2014 to 2022 between two African universities. The authors report on qualitative research within the leading collaborative scientists involved in major institutional research, their views on South-South collaboration, and the factors that consolidate their decision-making about joining and participating actively in research networks. |
| **In what ways is this article relevant to the candidate programme theories, if at all? (Include specific pages, paragraph, line)**  **Context**   - Fairness includes recognition of expertise and scientific roles of less visible partners - Adequate funding - Exchange of research material and sample between collaborators - Mobility of the researchers - Maintaining qualified research staff through tenure tracks help retain the talent - Competence in and commitment to good scientific practice   **Mechanisms**   - Researchers will have the commitment and enthusiasm needed to maintain a stable collaborative partnership - Researchers will have resources at their disposal necessary for developing/submitting grant proposals and research manuscript   **Outcomes**   - Increased research outputs including Masters, PhD trainees and research publications jointly produced by collaborators - Collaborative grants submissions - Infrastructural support - Extended collaboration with other Institutions out of the two countries |
| **What are the strengths and weaknesses of the article? Any red article?**  Most articles describe collaboration between institutions from the Global North collaborating with universities in Africa to strengthen the local research capacity, but this article is about South-South collaboration aimed at strengthening capacity in the least capacitated university.  Although the paper describes a few and fragmented C, M and O elements (without illustrating the link between the elements), the CMO components are relevant to research capacity strengthening in the context of an African university and therefore useful to the theory gleaning process. |
| **Describe any unintended positive or negative impacts and their mechanism link to the outcomes**  None |

**Definition of the categories**

The following definitions are only examples. These should be modified according to the needs of the study.

**HIGH**: This category is for papers that have high relevance to the realist synthesis. This means that the framing of the research and, the research questions are highly matched to the review questions, the empirical findings are clearly described and there is a rich description of the process and context that can greatly advance the theoretical output of the review. The paper is a ‘key informant’

**MODERATE**: This category is for studies that show a ‘moderately’ relevant framing of the primary research to the review theories. This may mean that the article reports on a different (but related) intervention working toward similar outcomes of interest or describes middle-range theories that may inform the review even if there is no relevant empirical data from the paper to populate the CMO configurations or has a few areas that are of interest even if it is not entirely clear whether they will be used in the synthesis.

**LOW**: This category is for research that has met the selection criteria in terms of relevance to the review questions and the initial programme theories (or MRT) but is relatively thin on the description of context and mechanism. It is not placed in the exclusion category because it contains at least one idea or statement about the context, about the mechanisms or about conceptualizing outcomes that can be used for refining the theory and building a CMO configuration.


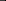


**EXCLUDE**: This category is for a research paper that showed promise on reading the citation, but upon reading the full-text paper does not correspond to the review questions, does not have any content that corresponds to the initial programme theories (or MRT), or does not describe at all the context, or the mechanisms (or process).

| **Record Number: 30** |
| --- |
| **Data extraction by: MNM**  **Data extraction date: 12 April 2024** |
| **Title**: Building a Research Culture from Scratch at a University of Technology |
| **First author/ year**: **Johnson & Louw** (2014) |
| **List companion papers (multiple papers of the same study incl. grey literature)**  None |
| **Relevance and usefulness of this study (see end of the form for definitions)**  High [✓] Moderate [ ] Low [ ] None [ ] |
| **Summary of the Paper**  **(What is this about? What kind of data source? Quant, Qual, MM, Blog, etc.**  This is a qualitative and descriptive paper that describes how research culture was built at an African university. The paper describes the contextual elements that influence and affect research culture, the enablers, challenges, etc. and how they shape culture building. |
| **What is interesting about this paper?**  The paper does not address any specific research discipline. It broadly describes building research culture in a university setting and qualitatively delineates different contextual and capacity outcomes, and to some extent mechanisms, that are relevant to the candidate programme theories specifically at institutional level. Although the paper describes a few and fragmented C, M and O elements (without illustrating the link between the elements), the CMO components are relevant to research capacity strengthening in the context of an African university and therefore useful to the theory gleaning process. |
| **In what ways is this article relevant to the candidate programme theories, if at all? (Include specific pages, paragraph, line)**  **Context**   - The central role of leadership – institutional leaders, deans, heads of departments and senior academics need to be talking, engaging, supporting, and encouraging researchers - Institutional policies and systems to support and incentivise the development of research (policies that ascribe value to research - More research time - often reflected in institutions’ and individuals’ grappling with the balance between teaching and research (p. 161).   **Mechanism**   - The agency of individuals (university researchers, staff and leaders) and their collective efforts - Researchers believe in their personal research abilities (confidence building) and start publishing their research work (improved soft skills) - Participants begin to have an understanding of research and to develop a wider scope of possibilities for research (p. 157).   **Outcomes**   - Researchers conduct high quality research, generate increased research outputs which strengthens the university research culture and profile - A critical mass of researchers with hands-on technical and soft skills is trained and which directly contributes to the university research enterprise |
| **What are the strengths and weaknesses of the article? Any red article?**  C, M and O elements relevant to institutional RCS reported. Additional insights relevant to the theorising process: Institutional policies and systems that ascribe value to research will: incentive research outputs including supervision incentives, strengthening research award systems, enabling national and international travel, aggressively recruiting postdoctoral fellows to further stimulate a scholarship discourse alongside encouraging attracting visiting professors, scholars and lecturers to the institution. |
| **Describe any unintended positive or negative impacts and their mechanism link to the outcomes**  None |

**Definition of the categories**

The following definitions are only examples. These should be modified according to the needs of the study.

**HIGH**: This category is for papers that have high relevance to the realist synthesis. This means that the framing of the research and, the research questions are highly matched to the review questions, the empirical findings are clearly described and there is a rich description of the process and context that can greatly advance the theoretical output of the review. The paper is a ‘key informant’

**MODERATE**: This category is for studies that show a ‘moderately’ relevant framing of the primary research to the review theories. This may mean that the article reports on a different (but related) intervention working toward similar outcomes of interest or describes middle-range theories that may inform the review even if there is no relevant empirical data from the paper to populate the CMO configurations or has a few areas that are of interest even if it is not entirely clear whether they will be used in the synthesis.

**LOW**: This category is for research that has met the selection criteria in terms of relevance to the review questions and the initial programme theories (or MRT) but is relatively thin on the description of context and mechanism. It is not placed in the exclusion category because it contains at least one idea or statement about the context, about the mechanisms or about conceptualizing outcomes that can be used for refining the theory and building a CMO configuration.


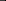


**EXCLUDE**: This category is for a research paper that showed promise on reading the citation, but upon reading the full-text paper does not correspond to the review questions, does not have any content that corresponds to the initial programme theories (or MRT), or does not describe at all the context, or the mechanisms (or process).

| **Record Number:** 31 |
| --- |
| **Data extraction by:** MNM  **Data extraction date:** 13 April 2024 |
| **Title**: Challenges and successes of research capacity building at a rural South African university |
| **First author/ year**: Singh (2015) |
| **List companion papers (multiple papers of the same study incl. grey literature)**  None |
| **Relevance and usefulness of this study (see end of the form for definitions)**  High [ ] Moderate [✓] Low [ ] None [ ] |
| **Summary of the Paper**  **(What is this about? What kind of data source? Quant, Qual, MM, Blog, etc.**  The paper utilised mixed methods. The secondary data was obtained from research statistics compiled by the university, approved policy and incentive documents of the university, and evaluative and reflective feedback from participants in different support programmes. The data was analysed both quantitatively and qualitatively. |
| **What is interesting about this paper?**  The paper describes the approach used by an African university in building research capacity such as increasing the number of accredited publications by staff; training supervisors; increasing the number of staff who have doctorates; providing support for female researchers; increasing awareness around funding opportunities; providing research support for postgraduate students and incentives for staff who publish and supervise postgraduate students. Although the paper describes a few and fragmented C, M and O elements (without illustrating the link between the elements), the CMO components are relevant to research capacity strengthening in the context of an African university and therefore useful to the theory gleaning process. |
| **In what ways is this article relevant to the candidate programme theories, if at all? (Include specific pages, paragraph, line)**  **Context**   - Historically disadvantaged university (inadequately funded) - Incentives and awards for staff engaged in research   **Outcomes**   - Research funding secured - High quality research produced in the minimum prescribed time   **Mechanism**   - Researchers have the skills (technical and soft skills) needed to thrive in research - Researchers are based in positive research environment |
| **What are the strengths and weaknesses of the article? Any red article?**  The paper describes a multi-pronged approach/strategies adopted by a South African university to build its research capacity. The paper, however, does not delinate contextual elements (although it mentions that rural universities are ‘historically disadvantaged’. |
| **Describe any unintended positive or negative impacts and their mechanism link to the outcomes**  None |

**Definition of the categories**

The following definitions are only examples. These should be modified according to the needs of the study.

**HIGH**: This category is for papers that have high relevance to the realist synthesis. This means that the framing of the research and, the research questions are highly matched to the review questions, the empirical findings are clearly described and there is a rich description of the process and context that can greatly advance the theoretical output of the review. The paper is a ‘key informant’

**MODERATE**: This category is for studies that show a ‘moderately’ relevant framing of the primary research to the review theories. This may mean that the article reports on a different (but related) intervention working toward similar outcomes of interest or describes middle-range theories that may inform the review even if there is no relevant empirical data from the paper to populate the CMO configurations or has a few areas that are of interest even if it is not entirely clear whether they will be used in the synthesis.

**LOW**: This category is for research that has met the selection criteria in terms of relevance to the review questions and the initial programme theories (or MRT) but is relatively thin on the description of context and mechanism. It is not placed in the exclusion category because it contains at least one idea or statement about the context, about the mechanisms or about conceptualizing outcomes that can be used for refining the theory and building a CMO configuration.


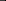


**EXCLUDE**: This category is for a research paper that showed promise on reading the citation, but upon reading the full-text paper does not correspond to the review questions, does not have any content that corresponds to the initial programme theories (or MRT), or does not describe at all the context, or the mechanisms (or process).

| **Record Number:** 32 |
| --- |
| **Data extraction by:** MNM  **Data extraction date:** 15 April 2024 |
| **Title**: Fostering global primary care research: a capacity-building approach |
| **First author/ year**: Ponka et al. (2020). |
| **List companion papers (multiple papers of the same study incl. grey literature)**  None |
| **Relevance and usefulness of this study (see end of the form for definitions)**  High [ ] Moderate [✓] Low [ ] None [ ] |
| **Summary of the Paper**  **(What is this about? What kind of data source? Quant, Qual, MM, Blog, etc.**  A reflection paper that examines five case studies of research capacity strengthening including in an African university context. |
| **What is interesting about this paper?**  The paper analyses five case studies on research capacity strengthening. The paper arose from a workshop held in 2019 addressing research capacity building in LMICs. Five case studies (three from Africa, one from South-East Asia and one from South America) illustrate tensions and solutions to strengthening research capacity around the world. The case studies exemplify that research capacity can be strengthened at the micro (practice), meso (institutional) and macro (national policy and international collaboration) levels. It mainly describes the contextual conditions required for effective research capacity outcomes to be realised. |
| **In what ways is this article relevant to the candidate programme theories, if at all? (Include specific pages, paragraph, line)**  **Context**   - Fully established research infrastructure may be realised when local practice-based research networks are connected to universities and research institutes. - Interaction between universities and research institutes – essential in securing joint ownership of research, and critical for impactful and sustainable research - Tendency for universities to want trainees to work independently on research - Transdisciplinary rather than mono-disciplinary approaches - Protected research time [time dedicated to research] (p. 4) |
| **What are the strengths and weaknesses of the article? Any red article?**  The paper describes macro-level issues vary contexts in that the agency to affect change at this level often resides in more resourced contexts. Although the paper describes a few contextual (C) elements (without illustrating the link between the elements), the context elements are relevant to research capacity strengthening in the context of an African university and therefore useful to the theory gleaning process. |
| **Describe any unintended positive or negative impacts and their mechanism link to the outcomes**  None |

**Definition of the categories**

The following definitions are only examples. These should be modified according to the needs of the study.

**HIGH**: This category is for papers that have high relevance to the realist synthesis. This means that the framing of the research and, the research questions are highly matched to the review questions, the empirical findings are clearly described and there is a rich description of the process and context that can greatly advance the theoretical output of the review. The paper is a ‘key informant’

**MODERATE**: This category is for studies that show a ‘moderately’ relevant framing of the primary research to the review theories. This may mean that the article reports on a different (but related) intervention working toward similar outcomes of interest or describes middle-range theories that may inform the review even if there is no relevant empirical data from the paper to populate the CMO configurations or has a few areas that are of interest even if it is not entirely clear whether they will be used in the synthesis.

**LOW**: This category is for research that has met the selection criteria in terms of relevance to the review questions and the initial programme theories (or MRT) but is relatively thin on the description of context and mechanism. It is not placed in the exclusion category because it contains at least one idea or statement about the context, about the mechanisms or about conceptualizing outcomes that can be used for refining the theory and building a CMO configuration.


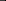


**EXCLUDE**: This category is for a research paper that showed promise on reading the citation, but upon reading the full-text paper does not correspond to the review questions, does not have any content that corresponds to the initial programme theories (or MRT), or does not describe at all the context, or the mechanisms (or process).
