# Supplementary material for: Eliciting initial programme theories for a health research capacity strengthening initiative targeting African universities: A realist synthesis
Source: PLoS One. 2025 Aug 20;20(8):e0330360. doi: 10.1371/journal.pone.0330360 (PMC12367166; doi:10.1371/journal.pone.0330360)
Supplement: Attachment — Submitted filename: PONE-D-24-49110.pdf [file pone.0330360.s005.pdf]

PLOS ONE

Theorising health research capacity strengthening of African universities: A realist synthesis

--Manuscript Draft--

**Manuscript Number:**	PONE-D-24-49110
**Article Type:**	Research Article
**Full Title:**	Theorising health research capacity strengthening of African universities: A realist synthesis
**Short Title:**	Theorising health research capacity strengthening of African universities: A realist synthesis
**Corresponding Author:**	Meshack Nzesei Mutua<br>Liverpool School of Tropical Medicine<br>Liverpool, UNITED KINGDOM OF GREAT BRITAIN AND NORTHERN IRELAND
**Keywords:**	realist approach; initial programme theory; CMO configuration; health research capacity strengthening
**Abstract:**	There is a growing call for theory-driven evaluation approaches to health research capacity strengthening (HRCS) interventions. Specifically, the realist evaluation has gained wide attention given its response to the question – how does an intervention work, why, for whom and under what conditions? In realist evaluation, initial programme theories (IPTs) are first elicited before they are tested and refined. This article describes the IPTs of a HRCS programme aimed at strengthening research capacity of African universities. Using the 'Developing Excellence in Leadership, Training and Science (DELTAS Africa) programme as a real-world case, the IPTs were drafted through a review of the programme documents and other published literature. Seven programme documents and thirty-two published papers covering twenty-six research capacity strengthening initiatives in African universities were reviewed. Different Context, Mechanism and Outcomes (CMO) were extracted and CMO configurations formulated. Thereafter, the CMO configurations were refined through four interviews with the DELTAS programme designing team. Three transferrable IPTs were elicited. Evidence suggests that, for HRCS interventions to be more effective in strengthening research capacity in African universities, systemic challenges (e.g., lack of funding for health research, ineffective research policy environment and lack of institutional support for research) need to be addressed and the university staff/ researchers empowered, incentivised and motivated. The article underscores the importance of institutional buy-in, effective implementation of research policies (e.g., protected time, research career pathways, gender equality, research ethics and integrity, anti-bullying and anti-harassment, etc.), long term research funding and equitable research partnerships in fostering a strong research environment and culture. Notably, the article makes a methodological contribution by demonstrating how IPTs can be developed using disparate evidence sources. The IPTs will be tested and refined through a primary realist evaluation which will further refine the CMOs presented in this article and provide insights into the current HRCS evaluation framework.
**Order of Authors:**	Meshack Nzesei Mutua
	Andrew Harding, PhD
	Justin Pulford, PhD
**Additional Information:**	
**Question**	**Response**
**Financial Disclosure**<br><br>Enter a financial disclosure statement that describes the sources of funding for the work included in this submission. Review	Yes

Powered by Editorial Manager® and ProduXion Manager® from Aries Systems Corporation

the submission guidelines for detailed requirements. View published research articles from *PLOS ONE* for specific examples.<br><br>This statement is required for submission and **will appear in the published article** if the submission is accepted. Please make sure it is accurate.<br><br>**Funded studies**<br>Enter a statement with the following details:<br>• Initials of the authors who received each award<br>• Grant numbers awarded to each author<br>• The full name of each funder<br>• URL of each funder website<br>• Did the sponsors or funders play any role in the study design, data collection and analysis, decision to publish, or preparation of the manuscript?<br><br>Did you receive funding for this work?	
Please add funding details.<br>as follow-up to "**Financial Disclosure**<br><br>Enter a financial disclosure statement that describes the sources of funding for the work included in this submission. Review the submission guidelines for detailed requirements. View published research articles from *PLOS ONE* for specific examples.<br><br>This statement is required for submission and **will appear in the published article** if the submission is accepted. Please make sure it is accurate.	Medical Research Council (MRC) Doctoral Training Award

**Funded studies**<br>Enter a statement with the following details:<br>• Initials of the authors who received each award<br>• Grant numbers awarded to each author<br>• The full name of each funder<br>• URL of each funder website<br>• Did the sponsors or funders play any role in the study design, data collection and analysis, decision to publish, or preparation of the manuscript?<br><br>Did you receive funding for this work?"	
Please select the country of your main research funder (please select carefully as in some cases this is used in fee calculation).<br>as follow-up to "**Financial Disclosure**<br><br>Enter a financial disclosure statement that describes the sources of funding for the work included in this submission. Review the submission guidelines for detailed requirements. View published research articles from *PLOS ONE* for specific examples.<br><br>This statement is required for submission and **will appear in the published article** if the submission is accepted. Please make sure it is accurate.<br><br>**Funded studies**<br>Enter a statement with the following details:<br>• Initials of the authors who received each award<br>• Grant numbers awarded to each author<br>• The full name of each funder<br>• URL of each funder website<br>• Did the sponsors or funders play any role in the study design, data collection and analysis, decision to publish, or preparation of the manuscript?<br><br>Did you receive funding for this work?"	UNITED KINGDOM - GB
**Competing Interests**	The authors have declared that no competing interests exist.

Use the instructions below to enter a competing interest statement for this submission. On behalf of all authors, disclose any competing interests that could be perceived to bias this work—acknowledging all financial support and any other relevant financial or non-financial competing interests.<br><br>This statement is required for submission and **will appear in the published article** if the submission is accepted. Please make sure it is accurate and that any funding sources listed in your Funding Information later in the submission form are also declared in your Financial Disclosure statement.<br><br>View published research articles from *PLOS ONE* for specific examples.<br><br>**NO authors have competing interests**<br><br>Enter: *The authors have declared that no competing interests exist.*<br><br>**Authors with competing interests**<br><br>Enter competing interest details beginning with this statement:<br><br>*I have read the journal's policy and the authors of this manuscript have the following competing interests: [insert competing interests here]*<br><br>* typeset	
**Ethics Statement**<br><br>Enter an ethics statement for this submission. This statement is required if the study involved:<br><br>• Human participants<br>• Human specimens or tissue<br>• Vertebrate animals or cephalopods<br>• Vertebrate embryos or tissues	Ethics approval was granted by both the Liverpool School of Tropical Medicine, UK, and Strathmore University, Kenya, Research Ethics Committees.

Powered by Editorial Manager® and ProduXion Manager® from Aries Systems Corporation

• Field research<br><br>Write "N/A" if the submission does not require an ethics statement.<br><br>General guidance is provided below. Consult the submission guidelines for detailed instructions. **Make sure that all information entered here is included in the Methods section of the manuscript.**	

**Format for specific study types**<br><br>**Human Subject Research (involving human participants and/or tissue)**<br>• Give the name of the institutional review board or ethics committee that approved the study<br>• Include the approval number and/or a statement indicating approval of this research<br>• Indicate the form of consent obtained (written/oral) or the reason that consent was not obtained (e.g. the data were analyzed anonymously)<br><br>**Animal Research (involving vertebrate animals, embryos or tissues)**<br>• Provide the name of the Institutional Animal Care and Use Committee (IACUC) or other relevant ethics board that reviewed the study protocol, and indicate whether they approved this research or granted a formal waiver of ethical approval<br>• Include an approval number if one was obtained<br>• If the study involved *non-human primates*, add *additional details* about animal welfare and steps taken to ameliorate suffering<br>• If anesthesia, euthanasia, or any kind of animal sacrifice is part of the study, include briefly which substances and/or methods were applied<br><br>**Field Research**<br><br>Include the following details if this study involves the collection of plant, animal, or other materials from a natural setting:<br>• Field permit number<br>• Name of the institution or relevant body that granted permission	
**Data Availability**<br><br>Authors are required to make all data underlying the findings described fully available, without restriction, and from the time of publication. PLOS allows rare exceptions to address legal and ethical concerns. See the PLOS Data Policy and FAQ for detailed information.	No - some restrictions will apply

A Data Availability Statement describing where the data can be found is required at submission. Your answers to this question constitute the Data Availability Statement and **will be published in the article**, if accepted.<br><br>**Important:** Stating 'data available on request from the author' is not sufficient. If your data are only available upon request, select 'No' for the first question and explain your exceptional situation in the text box.<br><br>Do the authors confirm that all data underlying the findings described in their manuscript are fully available without restriction?	
**Describe where the data may be found in full sentences. If you are copying our sample text, replace any instances of XXX with the appropriate details.**<br><br>• If the data are **held or will be held in a public repository**, include URLs, accession numbers or DOIs. If this information will only be available after acceptance, indicate this by ticking the box below. For example: *All XXX files are available from the XXX database (accession number(s) XXX, XXX.)*.<br>• If the data are all contained **within the manuscript and/or Supporting Information files**, enter the following: *All relevant data are within the manuscript and its Supporting Information files.*<br>• If neither of these applies but you are able to provide **details of access elsewhere**, with or without limitations, please do so. For example:<br><br>*Data cannot be shared publicly because of [XXX]. Data are available from the XXX Institutional Data Access / Ethics Committee (contact via XXX) for researchers who meet the criteria for access to confidential data.*<br><br>*The data underlying the results presented in the study are available from (include the name of the third party*	All relevant data are within the manuscript and its Supporting Information files.

and contact information or URL).<br>• This text is appropriate if the data are owned by a third party and authors do not have permission to share the data.<br><br>* typeset	
Additional data availability information:	

Powered by Editorial Manager® and ProduXion Manager® from Aries Systems Corporation

Manuscript
Click here to access/download;Manuscript;Manuscript.docx

Theorising health research capacity strengthening of African universities: A realist synthesis

Meshack Nzesei Mutua[1*], Andrew Harding[2], Justin Pulford[1]

[1]Centre for Capacity Research, Liverpool School of Tropical Medicine, UK
[2]Division of Health Research, Lancaster University, UK

***Corresponding author**
Email: Meshack.Mutua@lstmed.ac.uk (MNM)

Abstract

There is a growing call for theory-driven evaluation approaches to health research capacity strengthening (HRCS) interventions. Specifically, the realist evaluation has gained wide attention given its response to the question – how does an intervention work, why, for whom and under what conditions? In realist evaluation, initial programme theories (IPTs) are first elicited before they are tested and refined. This article describes the IPTs of a HRCS programme aimed at strengthening research capacity of African universities. Using the 'Developing Excellence in Leadership, Training and Science (DELTAS Africa) programme as a real-world case, the IPTs were drafted through a review of the programme documents and other published literature. Seven programme documents and thirty-two published papers covering twenty-six research capacity strengthening initiatives in African universities were reviewed. Different Context, Mechanism and Outcomes (CMO) were extracted and CMO configurations formulated. Thereafter, the CMO configurations were refined through four interviews with the DELTAS programme designing team. Three transferrable IPTs were elicited. Evidence suggests that, for HRCS interventions to be more effective in strengthening research capacity in African universities, systemic challenges (e.g., lack of funding for health research, ineffective research policy environment and lack of institutional support for research) need to be addressed and the university staff/ researchers empowered, incentivised and motivated. The article underscores the importance of institutional buy-in, effective implementation of research policies (e.g., protected time, research career pathways, gender equality, research ethics and integrity, anti-bullying and anti-harassment, etc.), long term research funding and equitable research partnerships in fostering a strong research environment and culture. Notably, the article makes a methodological contribution by demonstrating how IPTs can be developed using disparate evidence sources. The IPTs will be tested and refined through a primary realist evaluation which will further refine the CMOs presented in this article and provide insights into the current HRCS evaluation framework.

Introduction

Health research capacity strengthening (HRCS) has been defined by Lansang and Dennis (1) as the “ongoing process of empowering individuals, institutions, organisations and nations to: define and prioritise problems systematically; develop and scientifically evaluate appropriate solutions and share and apply the knowledge generated” (p. 764). HRCS is a global strategy aimed at improving the ability of researchers, research institutions and research users in low-and middle-income countries (LMICs) to carry out and promote use of research evidence that is geared towards addressing health challenges and needs of the population (2).

HRCS efforts have been categorised into three levels; namely, individual, institutional and environmental/ societal levels. Evidence shows that majority of HRCS interventions happen at the individual level (3-5) including activities such as scholarships, specialised training (e.g., on research design, grant writing and publication), mentorship and industrial placements. These individual level HRCS interventions are deemed not only as less complex (6) but also generate less impact compared to institutional and societal interventions (7). Institutional-level HRCS interventions seek to strengthen the capacity of research departments or units in universities or research institutions to effectively play their role within the national health research system (8, 9). Examples include upgrading research infrastructure (e.g., laboratory equipment, computers and data management infrastructure), strengthening administrative capacity (e.g., in finance, grants and research management functions), establishing career pathways for researchers (7). Societal-level HRCS interventions seek to improve the research system by ensuring relevant research policies are adopted that will incentivise individuals and organisations to conduct high quality research, stimulate positive political will and promote effective regulatory environment for research (5). Examples include efforts to improve the ability of research entities to manage transparent, efficient, and competitive processes for allocating national research funds and

demonstrate research productivity (such as funds, publications, patents), expansion of networks/ collaborations by national/international research bodies, and policy engagements that influence research uptake (5). The effects of institutional HRCS interventions interlink both individual and institutional level capacities with ripple effects that extend beyond the research system (5, 10) thus making such interventions complex and multifaceted in both design and implementation (11-15).

The biggest challenge, therefore, is to sustainably enhance the research capacity at institutional and societal levels which potentially generate greater impact at systems level (16, 17). This is exacerbated by the fact that there is underdeveloped evidence base on how to best design, implement and evaluate such interventions. The nature of the evaluation approaches coupled with relatively few evaluations in HRCS could partly be blamed for the existing evidence gap. Most evaluations of the HRCS interventions have used traditional approaches which do not provide evidence on how HRCS interventions work, across different contexts, to generate research capacity outcomes (18, 19). Yet, as complex, dynamic and multifaceted initiatives, HRCS interventions demand for more complexity-aware evaluation approaches (6). An appropriate evaluation for complex interventions such as HRCS should not only focus on what works but also provide answers to why, how, for whom, and under what conditions; this is grounded in a realist approach to evaluation.

Realist evaluation first emerged from Pawson and Tilley (20) seminal work and is grounded in scientific realism. The approach is suitable for uncovering rich and transferable insights into the effects of complex interventions given its focus on generative causal mechanisms which are context dependent (20). The essence of realist evaluation, according to Pawson and Tilley (21), is not to provide a verdict as to whether a programme has succeeded or failed, rather to examine how a programme interacts with specific local contexts to trigger mechanisms necessary for generating intended or unintended outcomes. Essentially, realist evaluation uses the Context (C), Mechanisms (M) and Outcome (O) configuration as a tool to explore generative causation (22). In practice, realist evaluation starts with a programme

theory and ends with a refined programme theory (Pawson & Tilley, 1997). Initial programme theories (IPTs) should be elicited first before they are tested and refined.

In this article, we describe the IPTs of a HRCS programme aimed at strengthening health research capacity of African universities. The aim is to elicit transferrable IPTs that can be applied in African university settings to inform the design, implementation, and evaluation of HRCS interventions thus ensuring that they can drive sustainable impact. A detailed protocol for this synthesis has been published elsewhere (23). The IPTs were developed through a phased realist synthesis process involving a) a realist review of a HRCS programme documents and published literature on research capacity strengthening of African universities, and b) interviews with the programme's designing team/partners. The 'Developing Excellence in Leadership, Training and Science (DELTAS Africa)[i] programme, one of the Africa's HRCS flagship initiative, was used as a real-world HRCS case.

In terms of structure, the paper first describes the realist review process, the review findings and how the IPTs were drafted by framing a causal link between context, mechanism and outcomes. Findings from the interviews (with programme designers) are then presented and a description of how the IPTs were further refined provided. The CMOs are then discussed – bringing in additional perspectives from published literature to shed more light on institutional HRCS practice – and a conclusion presented.

Stage 1: Realist Review

A realist approach seeks to establish what works, how, why, for whom and under what circumstances (20, 24-26). Realist review, also referred to as realist synthesis, is a form of systematic literature review that uses secondary data to understand complex interventions (25). Realist reviews have been used to both develop IPTs (27), and test and refine them (28-30). In this study, the review aimed at eliciting the IPTs by identifying contexts, mechanisms and outcomes that have been described by a) the DELTAS Africa programme

documents and, b) other published literature that has examined research capacity strengthening (RCS) initiatives implemented in African universities. The review sought to address three questions:

1. What are the key mechanisms that drive the outcomes of health research capacity strengthening for universities in Africa?
2. What are the contextual factors that enable the triggering of mechanisms necessary for generating health research capacity outcomes for African universities?
3. What is the causal connection between the key mechanisms, contexts, and the health research capacity outcomes?

Search Strategy and Selection of Papers

Firstly, relevant DELTAS Africa programme documents were requested from the DELTAS programme staff and seven (7) documents including grantee funding proposals, programme Theory of Change (ToC) and M&E Framework documents were shared. A few potentially relevant documents e.g., grantees progress reports and consolidated programme reports were not shared as the programme staff cited lack of copies of the reports. The programme documents were reviewed to gather insights about how the programme was designed and identify the underlying assumptions. The documents mainly described the programme architecture and partially the desired research capacity outcomes and some contextual elements. The ToC document presented an overarching high-level model of DELTAS programmatic domains namely scientific quality, research leadership, scientific citizenship and research management, culture and infrastructure and their related activities, outputs, and outcomes. Notably, none of the documents articulated or described mechanisms, though this was unsurprising because the programme was not designed with a realist lens. However, through the review process, a better understanding of the DELTAS Africa activities, who was targeted by them and to generate what effect or outcomes was gained. This information was extracted as themes and analysed and synthesised together with the information extracted from the published literature (see below).

Secondly, the published literature were identified through searches on electronic databases. Specifically, this involved a search on Google Scholar, Global Health, PubMed, and Web of Science using the search terms (((“research capacity strengthen*” AND “university*” AND “Africa”) AND (“research capacity build*” AND “university” AND “Africa”) AND (“research capacity develop*” AND “university” AND “Africa”))). The ‘Health Research Policy and Systems’ journal which publishes scholarly work focused on health research capacity, systems, and policies was also searched. The journal was searched using two key words i.e., “university” and “Africa” and then the results were screened together with the rest of the records identified from databases. The search included literature published in English and from the earliest records available in the databases up to 10 February 2024. EndNote 20 was used to organise and screen the results/references.

Papers were considered eligible for inclusion if they reported 1) research capacity strengthening programmes/ initiatives, 2) the initiatives were implemented any time prior to 2024, 3) in an African setting, 4) university as (one of) the study setting. Different publication types such as empirical studies and commentaries were included.

Quality Appraisal

Although article quality was not an inclusion criterion, the appraisal form by Molitor, Busse (31) was used to assess the relevance and quality of the selected papers and how they contribute to the theory building process. None of the papers made the connection between the context, mechanism, and outcomes, although each of the papers described at least one of the CMO elements. The majority of the empirical papers included in the review provided a detailed description of the research objectives, methods, and participants to the extent that the study could be replicated. Notwithstanding, the insights and themes extracted from the commentary papers were triangulated with those extracted from the empirical papers thus strengthening the review output.

Data Extraction and Analysis

An Excel template was used to extract data on the characteristics of the included papers and the RCS programmes reported including the first-author's affiliation, the subject focus, the RCS levels (individual, institutional or societal) focused by the paper, the funding source and the research methods employed by the studies. A Word version of the form developed by Molitor, Busse (31) was used to extract data on context, mechanism, and outcomes of RCS initiatives, allowed the articulation of the connection between the outcomes and process [C+M=O] and articulation of rival theory. Each paper was read twice to fully familiarise with the themes.

Thematic analysis was applied which allowed for systematic identification of recurrent or salient themes across the included papers, consistent with the review by Mukumbang, Van Belle (32). The themes were then categorised as mechanism, outcomes, and context. Since the extraction of the themes was not guided by already defined programme theories, inductive reasoning was applied and this helped to ensure that alternative ways of making sense of the data were not overlooked (33, 34). Once the extraction of the themes was completed, the data were organised in a three-column table with Column 1 capturing 'Context', Column 2 'Mechanism' and Column 3 'Outcomes'. This reorganisation, combined with retroductive reasoning, facilitated the analysis and synthesis of the data. A synthesis of the extracted data was carried out identifying commonalities, differences and associations across themes and papers, and this informed the framing of the CMO configurations. Discussions between the co-authors, whose expertise interface the realist approach and HRCS, helped with sense-checking throughout the process.

Review Results

Description of Included Papers

The electronic search resulted in 2408 papers, of which 1167 were duplicates (Figure 1 below). Titles and abstracts of 1241 records were screened against the eligibility criteria and 1022 results were eliminated. The full text for 219 results were retrieved and read, out of which 32 papers (19 empirical and 13 commentaries) were included for review.

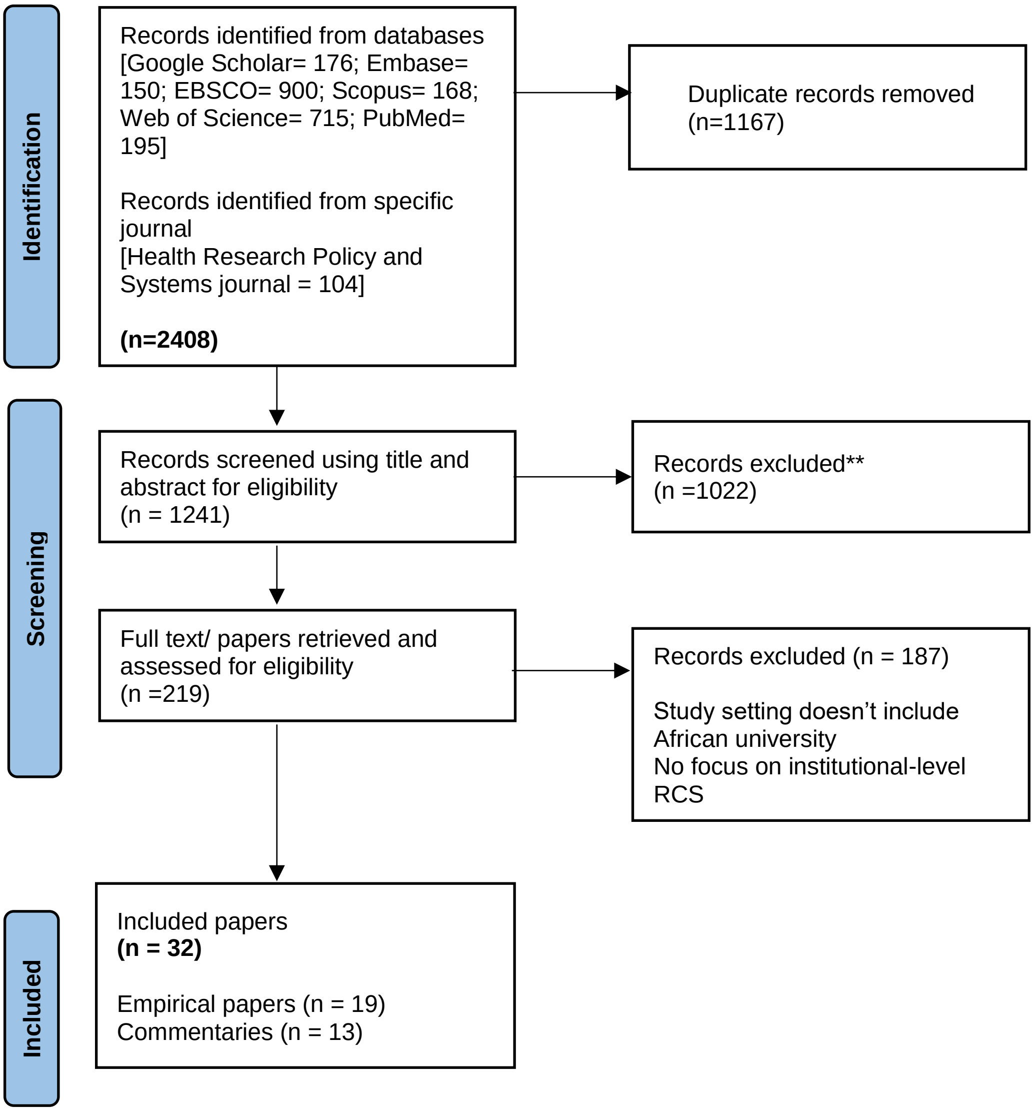

Figure 1: Papers selection and screening process – adapted from Page, McKenzie (35).

Summary characteristics of the included papers

The papers were published between 2010 and 2023, with the papers describing RCS programmes or initiatives that were implemented between the year 1998 and 2022. The 19 empirical papers included 13 papers that employed qualitative methods (in-depth interviews), 3 papers that employed mixed methods (surveys and in-depth interviews), 2 papers that involved review or analysis of secondary evaluation data and 1 paper that employed quantitative method (survey) only. The 13 commentaries included authors' reflections on the experience of implementing a RCS initiative, the successes, challenges, enabling and hindering factors. Twenty-one (21) papers were first authored by researchers affiliated to an African research institution/university while 11 papers were first authored by individuals affiliated to non-African institutions. Twenty-eight (28) papers were published in journals based in the Global North and four (4) were published in African-based journals.

Table 1: Characteristics of the included papers

**Category**	**Characteristic**	**Description**	**Frequency and Percentage**
Publication characteristics	Type of publication	Empirical	19 (59.4%)
		Commentary	13 (40.6%)
	First author affiliation	African	21 (66.6%)
		Non-African	11 (34.4%)
	Journal	African based journal	4 (12.5%)
		Non- African based journal	28 (87.5%)
Programme characteristics	Funding source	Global North funding partner	26 (81.3%)
		Not reported or anonymised	6 (18.7%)
	Subject focus	Health and disease-specific research capacity	26 (87.3%)
		General research capacity	6 (18.7%)
	Primary RCS levels	Institutional only	2 (6.3%)
		Individual and Institutional	21 (66.6%)
		Individual, Institutional and Societal	9 (28.1%)

Twenty-nine (29) papers described 26 different RCS initiatives implemented across African universities, in some cases covering both research institutions and universities. Three (3) papers did not describe any particular RCS programme or initiative, rather, they described

the practice of research systems strengthening in universities. Twenty-six (26) papers described programmes that were funded by Global North partners, five (5) papers did not report the source of funding and in one (1) paper the funding source was anonymised. Twenty-nine (29) papers described 'partnerships' as the RCS approach, out of which twenty-five (25) papers covered RCS as the primary objective of the partnerships. Different initiatives were led by different partners with majority (16) programmes involving South-North partnership, 8 initiatives involving North-South partnership and 5 initiatives involving South-South partnership. Two papers focused on RCS activities at institutional level only, 21 papers focused on RCS at both individual and institutional levels and 9 papers focused on RCS at three (individual, institutional and societal) levels. Twenty-six (26) papers focused on RCS in health-discipline or disease-specific research areas (e.g., HIV/AIDS, malaria, or mental health) and six (6) papers focused on RCS in general. It was impossible to determine the number of universities covered across the 32 papers and where they were based (regionally) because 12 papers had anonymised the names of the universities and the African region they are located.

Findings

The sub-sections below present the findings of the themes extracted from both the published literature and the DELTAS Africa programme documents. Where necessary, excerpts of the programme documents were extracted and included in the review findings.

MECHANISM

Although the focus remains 'research capacity strengthening at institutional level', the reported mechanisms were all triggered at individual level. This is consistent with Pawson and Tilley (20) argument that programmes do not work by themselves, rather, it is the decisions, reasoning, actions and reactions of the individuals directly or indirectly affected by a programmes that make it work or not. Multiple papers described the mechanisms of *trust* and *respect*, *commitment* and *enthusiasm*, *motivation* and *incentives*, *empowerment*, and

ownership and *buy-in* by institutional leaders that were triggered in specific contexts to generate RCS outcomes.

Trust and respect

Three papers (9.4%) reported trust and respect between researchers or research partners as an important mechanism that enable collaborations and partnerships in RCS to thrive (36-38) whether those collaborations or partnerships are between researchers from the Global North and African universities or between African universities. Any partnerships/ collaborations that are geared towards strengthening research capacity must appreciate and respect the local knowledge and priorities and this determines the effectiveness of those collaborations (37). Additionally, collaborators and partners should clearly lay out their expectations in their partnership agreement, appreciate each other's strengths and resources that they bring to the partnership (38) as this is key in building trust.

Commitment and enthusiasm

Three papers (9.4%) highlighted commitment and enthusiasm as a central mechanism for university faculty and postgraduate trainees taking on research careers and leveraging research collaborations (39-41). Researchers and research leaders must sustain the commitment and enthusiasm if a collaboration or partnership is to remain stable and deliver positive RCS outcomes (40, 41).

Motivation and incentives

Seven papers (21.9%) reported that the RCS programmes would be effective if the faculty are highly motivated, willing, and incentivised to drive substantive and sustainable institutional change at African universities (42-48). This would work where, for instance, universities provide research grants to faculty members as incentives to retain them (43) and where partnerships allow sharing of mutually beneficial research resources (e.g., sharing of lab space, research space and equipment) which is crucial for learning and research

production (46). However, individuals have to be motivated and willing to transfer the research training and skills to their institutions (47) which is key in institutional capacity strengthening.

In the DELTAS Africa programme, the selection of researchers for fellowship opportunities (across the consortia) considers their motivation levels and career ambitions, and then the programme provides them with the resources and incentives to strengthen their research capacity and research leadership. One of the documents captured the following:

> "Senior research fellows/ scientists who are motivated and have demonstrated high research productivity and leadership potential will be supported to consolidate their independent research and build a group at African partner universities" [DELTAS Programme Document 4].

Empowerment

Five papers (15.6%) highlighted that the empowerment of researchers – which enhanced their sense of agency – was key to translating their individual technical and soft skills and competencies to institutional capacity (44, 49-52). The respective RCS programmes aimed to impart – through training – committed and motivated researchers with wide ranging technical and soft skills, but the individual must believe in their personal capabilities and exercise their agency if high quality research is to be produced and institutional research goals met (52). By exercising their sense of agency, individually or collectively, the university staff and researchers will positively use their competencies and skills both to their advantage but also the institution's (50).

Ownership and buy-in by institutional leaders

Twelve papers (37.5%) have reported ownership and buy-in by institutional leaders as an important mechanism that influences the effectiveness of institutional RCS (36, 38-40, 42, 44, 46, 50, 52-55). This mechanism may be triggered in the context where equitable and

strategic partnerships are developed, and where institutional leaders feel that the RCS initiatives are addressing local priorities and needs and, importantly, have the autonomy or an influence on how the programme resources are utilised (36, 38, 42, 44).

As the institutional research capacity improves and the RCS initiatives improve institutional practices and culture, this mechanism may become part of the context where ownership and buy-in from university management and faculty staff would be a prerequisite for RCS initiatives to thrive (36, 43, 53, 56, 57). In such a case, for instance, RCS efforts that involve introduction and implementation of new policies, systems or initiatives will require institutional buy-in and support from the leaders if such efforts are to be institutionalised and sustained (53). Johnson and Louw (50) posit that, institutional leaders such as deans, heads of departments and senior academics need to be engaging, supporting, and encouraging faculty to pursue high quality and impactful research if the research culture is to grow (p. 159). Leadership practices were central to the provision of strategic direction which facilitated institutional-level RCS (53). Strong leadership allowed ownership that facilitated the introduction of necessary change in local institutions (54).

The DELTAS programme documents highlighted the critical role that institutional leadership play in RCS efforts and key strategic decisions such as allocation of resources. Ensuring that these leaders are on board is crucial if RCS initiatives are to be effective. One of the programme documents captured the following:

> "Vice Chancellors (VCs) are in a better position to support the institutionalisation plans that [researchers/staff] may be pursuing within their institutions. Examples of this include institutional investment in research infrastructure, changes to PhD training programmes at institution level, changes to promotion criteria, attention to academic integrity and taking on harm reduction strategies. … Regular communication/ engagement of the institutional leadership (e.g., through the biennial meetings with the VCs to present evidence on best practice learned and challenges

> encountered) will help garner support from the institutional leadership and achieve a buy-in” [DELTAS Programme Document 2].

OUTCOMES

The outcomes identified include increased research funding secured, improvement of research infrastructure and equipment, research management and governance systems strengthened, research partnerships strengthened, increased retention of research staff, research career and portfolio growth and improved health outcomes and policymaking. Notably, strained teaching and administrative resources was identified as an unintended negative outcome. Each of these are described below.

Increased research funding

Eleven (34.4%) papers reported securing additional funding as a result of improved research capacity at both individual and institutional levels. As the grant writing skills and research leadership of the university faculty/ researchers improve, they engage in the development of multidisciplinary research grant writing which may result in additional funding being secured (37, 39, 41-43, 45, 46, 49, 51, 53, 55). Notably, as the researchers continue to expand their networks, identify new collaborators, and develop collaborative grant applications then collaborative research grants end up being secured (41, 42, 46) which contribute to the institutional overheads.

Improvement of research infrastructure and equipment

Eight papers (25%) reported improvement of physical research infrastructure and equipment as an RCS outcome. Improvement of research infrastructure included procurement and accreditation of laboratory equipment (10, 36, 37, 42, 53), general infrastructure such as research office spaces upgraded (57, 58) and installation of interactive communication technologies that enabled the scaling up of training and research in the university (54). The

long-term impact of the infrastructure grant is that the improved research infrastructure and equipment remain at the universities for use by faculty long after the grant has ended (42).

The DELTAS Africa programme provides for infrastructure grants which contribute to the upgrading of the research infrastructure and procurement of research equipment. One of the programme documents captured the following:

> At the institutional level, partner universities may apply for "infrastructure grants that have in the past been used to upgrade research training infrastructure like postgraduate seminar rooms and libraries. The support has also been used to upgrade internet connectivity and ICT support equipment (projectors, computers, printer/scanner), and renovating training facilities, and purchase research equipment e.g., desktops/laptops in selected universities and anti-plagiarism software and statistical analysis packages" [DELTAS Programme Document 2].

Research management and governance systems strengthened

Thirteen papers (40.6%) reported that the RCS programmes had resulted in strengthened research management and governance systems of the participating institutions. This included development and adoption of research and grants management policies and procedures (36, 56), establishment of new research support units (38, 39, 54, 59) and improvement of research ethics processes (16, 37, 41, 46, 50, 51, 54, 57). Collaboration/partnerships that involve establishment of research support centres/units deliver a long term impact (50) as those units can support the wider research community beyond the originally intended university departments. With strong research management and governance systems in place, other ripple effects can be realised such as production of high-quality and impactful research and research outputs which can ultimately improve the institutional research profile (50).

> “Programme provides funds to establish 2-3 research hubs. These funds may be utilized to equip a research management office (computers, printers, router), improve seminar or meeting rooms to support research and research training activities, or provide seed funds for a research hub to buy small and medium-sized equipment. … In terms of software, the programme provided the initial institutional subscription to the anti-plagiarism software Turnitin with the partner institutions making a commitment to continue the subscription. The use of this software has now been incorporated in postgraduate training in all our partner institutions and so no further investments are necessary” [DELTAS Programme Document 2]

> Research Management/Support Offices/Units within the participating universities will drive the adoption of existing good practices, and support implementation at institutional level in the longer term. [DELTAS Programme Document 3]

Research institutes can have more established research management and governance infrastructures than universities in Africa (40) and collaboration between the two can help the university adapt and institutionalise research management functions (establishment of research support units) which can help with recovering costs from future successful grants thus making the university research enterprise sustainable in the long term.

Research partnerships strengthened

Five papers (12.5%) reported that institutional collaborations had been expanded and strengthened as a result of RCS efforts (41, 42, 53, 57, 59). These were synergistic research collaboration and partnerships mainly between African and Global North universities and research institutions. Some of the collaboration/ partnerships became long-standing or institutionalised at the university and expanded to benefit other departments or faculty that were not originally targeted by the initiative (42).

Strained teaching and administrative resources

One paper (3.1%) reported that some departments within the university had their teaching and administration resources overstretched because junior faculty had embarked on postdoctoral research training as part of institutional RCS (52). As an unintended negative outcome, this meant that other faculty/staff had to take on additional responsibilities to cover for their colleagues who had transitioned to postdoctoral training. Though not an outcome of RCS, this theme was identified as critical since it is a consequence of junior university staff transitioning or growing in their research careers which strains the available resources. Notably, this informs the framing of a rival theory.

Increased retention of research staff

One of the papers highlighted that in context where research career pathways are established, there is notable retention of faculty staff (59). Faculty staff who are promoted into research leadership positions (in context where research career pathways are established) inherently becoming agents of change in their universities and support the transformations of their universities into research centres of excellence (48). Established research career pathways in universities has also seen junior faculty staff undertaking PhDs, and postdoc fellows taking up research roles or promoted to research leadership roles in the universities. The ripple effect of the established career pathways was the individual researchers contributing to publication, mentoring junior faculty, increased supervisory capacity, increased university faculty staff with PhD qualifications, and securing of research grants (which contributes to institutional overheads) and this illustrates the interlinkage between individual level capacity and institutional capacity (10, 16, 37, 43, 45, 48, 50, 53, 57, 59).

In one of the DELTAS documents, it was reported that the RCS programme had introduced a new position into the university structure (postdoctoral research role) that were initially

supported by the programme. The postdoctoral fellows were subsequently absorbed into the formal university structure. Below is the excerpt.

> "The Consortium A team reported that discussions with University senior management about the need to regularise the postdoctoral position within the institutional faculty structure had positive result. Consequently, all the postdoctoral fellows from Phase I of the programme were absorbed into the University faculty system after completion of their fellowship, which is an indicator of progress towards institutionalization of the postdoctoral fellow position" [DELTAS Programme Document 1]

Research career and research portfolio growth

Five papers (15.6%) reported that research fellows who had been trained and mentored through RCS programme had progressed to occupy faculty positions and research leadership positions at the universities (37, 39, 47, 49, 58). This outcome relates to previously described outcomes such as staff retention and securing of additional funding which are consequence of individual level capacity being translated to institutional capacity. Notably, buy-in by institutional leaders and having established research policies, systems and culture in place was highlighted as key contextual element that allows for individual capacity to be institutionalised (42).

Improved health outcomes and policymaking

Eleven papers (34.4%) have acknowledged 'improved health outcomes of the population' (16, 38, 39, 44, 52, 60, 61) and 'impacts on policy and practice' (16, 38, 49, 58, 62) as the ultimate and long term outcomes of RCS. Although the authors recognise the limitation of their studies in that they did not document these long-term outcomes given that the studies were mostly conducted during the implementation of short-term RCS initiatives (42, 46, 53, 63); they highlighted that the goal of RCS is to generate impactful research that has utility in policy making and contributes to the improvement of people's health and wellbeing. To

achieve these ultimate outcomes, Torondel, Balls (44) posit that researchers and research entities should actively engage relevant actors throughout the research cycle, and ensure research is translated and used by stakeholders.

CONTEXT

The contextual elements identified include institutional research policies, systems, and culture; provisions for protected time for research; established research career pathways; established research infrastructure and equipment; reliable long-term funding; political and economic environment and equitable and strategic research partnerships. Each of these are described below.

Institutional research policies, systems, and culture

Seventeen papers (53.1%) reported the centrality of institutional research policies, systems, and culture, and how they enabled or hindered the effectiveness of RCS efforts and related outcomes (10, 16, 41, 44, 47, 49-51, 54-56, 61, 63, 64). Some of these research policies and systems may include provisions for protected time, establishment of research career pathways and research management and governance structures. Instruction-oriented universities are characterised by minimal financial and policy support for research and underdeveloped research governance and management policies (16, 49, 56) and navigating the complex university-level bureaucracy can be exhausting for researchers (16, 64). Since most African universities do not have established research policies and systems like other research institutions (16), interaction between universities and research institutes become an essential avenue for collaborative, impactful and sustainable research (61). Established institutional research policies, systems and culture can catalyse, support, and incentivise the development of research (50) thus further strengthening the institutional research culture (47).

Provisions for protected time

Twelve papers (37.5%) reported availability, or lack thereof, of protected time for research as a critical contextual element that determines the effectiveness of RCS activities. University faculty have to balance between their research and teaching workloads and mostly their research careers and interests are stifled by intensive teaching workloads (16, 36, 39, 46, 47, 50, 52, 54, 61-64). It is not a surprise that when junior faculty staff embark on doctoral or postdoctoral research training, it can result in unintended negative effect on the remaining faculty staff within their departments as they have to bear the additional teaching and administrative responsibilities relinquished by their colleagues (52).

For RCS initiatives to be effective, faculty staff must find a balance between research, teaching, clinical and administrative duties (39) as this will allow them to spend more time in research and RCS activities. In context where universities do not have provisions for protected time, RCS programmes are likely to be effective if they provide financial resources needed to buy out their teaching time, consequently enabling them to spend more time on research activities (36). In fact, provision for protected research time by universities has been viewed as an indicator of how established the research culture is at universities (46). One of the DELTAS Programme documents has highlighted that the participating universities are primarily teaching-centred which means that faculty staff will spend more time teaching than research and this explains why the research systems in those universities are less established. Below is a quote from one of the documents:

> "…universities where the institutional establishments are mainly teaching-centred and do not provide clear career paths for research-intensive personnel such as postdoctoral fellows. This serves as a disincentive for young scientists to take up such positions as an alternative to becoming lecturers and being burdened by high teaching loads which stifle their research careers" [DELTAS Document A]

Established research career pathways

Six papers (18.8%) have highlighted how 'established research career pathways' in a university setting influence how well RCS programmes deliver institutional research capacity outcomes (41, 45, 52, 62-64). Established research career pathways is characterised by, for instance, provision of tenure tracks to qualified research staff (alongside the academic career tracks on teaching and mentoring students) that helps retain the talent (41, 63, 64) and provision of professional development for research and research support staff (64). Without the established research career pathways, some of the effects would include having few junior academic staff with PhD qualifications (45, 52) and scarcity of senior faculty/researchers to mentor junior faculty staff, and the high burden of being a senior researcher having to bear the institutional research workloads (62). Established research career pathways ensure that there are adequate personnel contributing to a university's research pipeline. In some cases, this has been the primary strategy for strengthening research capacities in universities as the junior staff/ faculty are encouraged and motivated to pursue PhDs (60) and individuals are empowered to institutionalise research capacity in their universities (44). In fact, Bates, Boyd (65) argue that paying attention to the skills of individuals is important even if the main focus of a programme is to strengthen institutional systems and processes (p. 7), while Aiyenigba, Abomo (53) – who examined how the DELTAS Africa Phase One was strengthening research capacities of the participating African institutions (including universities) – concluded that, besides direct infrastructural development, other institutional-level RCS benefits were driven through investment in individuals.

Established research infrastructure and equipment

Eleven papers (34.4%) reported availability of physical research infrastructure and equipment as central to institutional RCS (16, 37, 42, 49, 52, 54, 58, 61-64). Sewankambo, Tumwine (52) argue that the research spaces can improve social interaction among research staff which can catalyse collaborative research activities. Dedicated learning spaces with strong internet connectivity are essential in promoting the research enterprise

(49). Importantly, since research institutions have more established research infrastructure and systems (16), universities that work closely with such research institutions will likely leverage their infrastructure (61). The authors – across the 11 papers – consistently argue that established research infrastructure and equipment allows high-quality research to be produced, and this has a ripple effect on the institutional research culture and the established infrastructure is foundational for acquisition of more funding resources. Modern communication technologies, for instance, that have been integrated in institutional infrastructure have supported open and fast sharing of information among researchers thus facilitating collaborative research work (37).

Reliable long-term funding available

Twelve papers (37.5%) reported that the RCS programmes were delivered in resource-constrained environments with limited or no funding from the local governments and primarily relying on external funding (16, 37, 39, 41, 42, 45, 47, 48, 53, 58, 63, 64). Limited resources not only affect almost all the RCS processes and the achievement of desired capacity outcomes but also implementation of the research activities (39). This lack of dedicated, long-term and sustainable local funding for research and RCS heavily undercuts both operational feasibility and human motivation (58). It is unsurprising that authors have called for increased long-term financial investment towards RCS from governments, private sector, and philanthropies if significant impact on RCS should be realised (16, 48, 58). To address the challenge of limited resources, research collaborations and partnerships were key to supporting research and RCS initiatives in resource-constrained settings (58). This allows sharing of research resources that are available in partner institutions (41).

Political and economic environment

Four papers (12.5%) highlighted how the wider political and economic environment can both influence RCS and also promote or inhibit research capacity (38, 51, 57, 59). The researchers highlight how unstable political, economic, and historical context affected

research partnerships, government buy-in and support for research and RCS, recruitment and retention of university faculty/ researchers and implementation of collaborative research. For instance, for research partnerships/ collaborations to foster real systemic change in RCS, the environment should be secure and stable enough to allow researchers' mobility and free interaction (57). These macro-level issues vary across contexts in that the agency to affect change often resides in more resourced and stable environments (61).

Equitable strategic research partnerships

Four papers (12.5%) reported how equitable and strategic research partnerships were central to the RCS efforts (41-43, 46). Partnerships are characterised by power dynamics and control given the differing expectations and the desire for financial control among partners (46). Power dynamics between Global North and African partners are more evident especially when the grants are managed by the Global North partners as this determines the level of autonomy or influence by African institutions in relation to when and how funds are spent (46) which triggers ownership and buy-in, or lack thereof, by the institutional leaders. To attain an equilibrium in research partnerships, there must be fairness in terms of recognition of expertise and research roles of less visible partners (41) and the grants should be flexible to allow investment in local priorities and needs (43).

Context Mechanism and Outcome: the causal link

Based on the contexts, mechanism and outcomes that emerged from the review process, the following possible generative causal explanations could be derived. Given the complexity of RCS initiatives which are implemented in complex environments, targeting multiple actors at different levels of individual and institutional capacities, and characterised by varied activities; it is likely that multiple contextual elements will provide a conducive environment where multiple mechanisms will simultaneously be triggered to generate multiple research capacity outcomes as described below.

a. In a context where the university faculty staff have no protected time for research and have limited or no access to well established physical research infrastructure and equipment and no established research career pathways, it is likely that research capacity strengthening efforts aimed at improving the infrastructure and equipment and research environment including research career pathways and protected time will likely motivate, incentivise, and empower the faculty staff and enact ownership and buy-in among university leaders. This will likely result in the adoption of policies on protected time and research career pathways and improved physical research infrastructure and equipment. In this case, where basic research capacity does not exist, the goal of RCS initiatives will be to build relevant infrastructure and develop relevant research policies.
b. In a context where the university faculty staff have protected time for research and have access to well established physical research infrastructure and equipment and based in university with established research career pathways, it is likely that research capacity strengthening efforts aimed at improving the infrastructure and equipment and research environment including research career pathways and protected time will likely motivate, incentivise, and empower the faculty staff. This will likely result in research career and portfolio growth. However, as the university's research portfolio expands, it is possible that the available teaching and administrative resources may be overstretched. As such, RCS efforts would be yielding an unintended negative outcome.
c. In a context where the faculty staff have protected time for research, access to well established physical research infrastructure and equipment and based in a university with established research policies, systems, and culture, research capacity strengthening efforts will likely motivate, incentivise, and empower faculty staff and spark commitment and enthusiasm among them to conduct high quality and impactful research. This will likely see the faculty staff record career growth in their research and research portfolio including securing additional research funding and

impactful research that improves people's health outcomes and influences policymaking being generated. In this case, a certain level of institutional research capacity is required to further strengthen that capacity which adds a layer in the complexity frame. Based on my experience supporting RCS programmes in Africa, most funding partners supporting RCS initiatives invariably require institutions to meet or demonstrate a minimum capacity level, for instance, in research governance and grants management before they can be funded. In such cases, therefore, RCS initiatives will be aimed at further strengthening the existing research capacity.

d. In context where a university has established equitable partnerships, has access to sustainable long-term funding and is based in a stable political and economic environment, research capacity strengthening efforts may trigger ownership and buy-in by institutional leaders and also trust and respect among researchers because the contextual conditions allow the researchers to address their local research capacity priorities and needs. This will likely result in strengthened research partnerships and research management and governance systems, and generation of impactful research that improves people's health outcomes and influences policymaking. For these long – term RCS outcomes to be realised, sustained funding, equitable and strategic partnerships and stable research environments are key.

Table 2: The four initial programme theories derived from literature review

Context	Mechanism	Outcome
If faculty staff have no protected time for research and are based in a university where there is lack or limited access to well established physical research infrastructure and equipment and no established research career pathways	Research grants and partnerships that support for upgrade of infrastructure and equipment, and adoption of institutional research policies such as research career pathways and protected time (resource) will likely motivate, incentivise, and empower faculty staff (reasoning) and enact ownership and buy-in among university leaders (reasoning)	Resulting in the adoption of policies on protected time and research career pathways; improved physical research infrastructure and equipment

If faculty staff have protected time for research and are based in a university where there is access to well established physical research infrastructure and equipment and established research career pathways	Research partnerships between highly capacitated and low capacitated universities/ research institutions that allow sharing of resources such as financial, technical expertise and infrastructure (resource) will likely motivate, incentivise, and empower them (reasoning)	Faculty staff record growth in their research career and portfolio, but this may strain their departmental teaching and administrative resources
If faculty staff have protected time for research, access to well established physical research infrastructure and equipment and based in an institution with established institutional research policies, systems, and culture	Research partnerships between highly capacitated and low capacitated universities/ research institutions that allow sharing of resources such as financial, technical expertise and infrastructure and provide mentorship for emerging researchers (resource) will likely motivate, incentivise, and empower, and enact commitment and enthusiasm among junior and senior faculty staff (reasoning)	Resulting in increased retention of faculty staff; increased research portfolio including research funding, and generation of impactful research that promotes positive health outcomes and policymaking
If a university has established equitable strategic research partnerships, has access to sustainable long-term funding and is based in a stable political and economic environment	Research partnerships between highly capacitated and low capacitated universities/ research institutions that allow sharing of resources such as financial, technical expertise and infrastructure (resource) will likely trigger ownership and buy-in among university leaders, and trust and respect among faculty staff (reasoning)	Research partnerships and research management and governance systems strengthened and generation of impactful research that promotes positive health outcomes and policymaking

Stage 2: Interviews with Programme Partners

Four (4) interviews with the DELTAS Africa Phase I programme designing team representatives were conducted to further refine the IPTs. The interviews were conducted between June and July 2024. The aim was to elicit their understanding of how the DELTAS programme (one of flagship multi-million dollar and long-term RCS initiative) was envisioned to work to generate the desired research capacity outcomes and how context moderates or mediates the outcomes. The participants also possessed extensive experience

implementing similar research capacity strengthening initiatives in Africa and therefore, the insights they provided were not limited to the DELTAS programme only. The realist interviewing technique was applied thus focusing the discussions on the IPTs.

After the interviews were conducted, the interview recordings were transcribed verbatim. The transcripts were then keenly read (first-level analysis) to gain a holistic understanding of the data. Afterwards, the transcripts were re-read (second-level analysis) and fragments of data were accordingly coded as context (C), strategy (S), mechanism (M) or outcomes (O). Retroductive theorising was applied to data analysis, employing both deductive and inductive perspectives. Deductive reasoning was applied to identify context, strategy, mechanism and outcomes (CMOs) that were aligned with the IPTs, and inductive reasoning was applied to identify potentially new CMO elements (not captured by the IPTs).

Following the analysis, all the contexts, strategy, mechanism and outcomes codes that were deductively (roman) and inductively (*italicised*) identified from the interview data were mapped – see *Table 4* below. The number of sources (transcripts) from which a C or M or O element was identified has been included in brackets.

Table 4: Contexts, strategy, mechanism and outcomes codes from interview data

Context	Strategy	Mechanism	Outcomes
Lack of research policies and systems e.g., protected time, research career pathways, *gender equity, research integrity and ethics anti-bullying and anti-harassment* (4)<br><br>Inadequate physical research	Research grants, partnerships and collaborations that support for upgrade of infrastructure and equipment (4)<br><br>*Advocacy for the adoption of institutional research policies e.g., and protected time (2)*	*Challenge/ inspire partner teams to adapt their own research policies and systems (3)*<br><br>Support for change/ improvement by university leaders including vice chancellors (VCs) and DVCs of research (2)	Policies on protected time, research career pathways, *gender equity, research integrity and ethics, anti-bullying and anti-harassment* adopted (3)<br><br>Improved physical research

infrastructure and equipment (4)<br><br>*Ownership and buy-in among university leaders including vice chancellors, DVC of research* (2)<br><br>Adequate physical research infrastructure and equipment (4)<br><br>Established research career pathways (2)<br><br>Established research policies and systems e.g., protected time, research career pathways, *gender equity, research integrity and ethics anti-bullying and anti-harassment* (4)<br><br>Equitable and strategic research partnerships (3)<br><br>Access to sustainable long-term funding (4)<br><br>Stable political and economic environment (2)	*[Specialised] training for faculty staff and researchers in grants writing, leadership, research partnerships* (3)<br><br>Research partnerships between highly capacitated and low capacitated universities that allow sharing of research resources (also referred as twinning) (4)	Motivate, incentivise, and empower faculty staff and researchers (4)<br><br>Trust and respect among faculty staff and researchers (3)<br><br>Commitment and enthusiasm among junior and senior faculty staff (2)	infrastructure and equipment (4)<br><br>Research career and portfolio growth recorded (3)<br><br>Departmental teaching and administrative resources stretched [challenged by participants] (2)<br><br>Increased retention of faculty staff (4)<br><br>Impactful research that promotes positive health outcomes and policymaking generated (4)<br><br>Research partnerships, research management and governance systems strengthened (2)<br><br>*Improved research culture* (2)

As shown in Table 4 above, most of the CMO elements were aligned to and supportive of the IPTs. In subsequent sub-sections, the new CMO elements inductively identified are described in detail, thus demonstrating how the participants voices were central to the

process. Thereafter, a description of how the new CMO elements informed the modification of IPTs is provided.

Context

i. Lack of critical research policies (e.g., gender equality, research ethics and integrity and anti-harassment and anti-bullying)

In contexts where critical research policies are lacking, participants highlighted that the DELTAS programme was keen to advocate for adoption of those policies (besides protected time and career pathways) including those related to gender equity, research ethics and integrity and anti-harassment and anti-bullying. These policy provisions are deemed as a basic foundation for effective research and which every university must have if a positive research culture is to be entrenched. Some of the participants said the following.

> Gender equity, research ethics and integrity, commitment to anti-harassment and anti-bullying culture is a few of the research culture issues that universities must deal with. DELTAS has been committed, from the go, to ensuring that relevant policies on research culture are adopted and implemented in partner institutions where such don’t exist. …DELTAS believes that each university should have these basic policies in place. [Participant 2]

> …it was not limited to protected time and research career promotion and pathway policies; it was broader than that. …we included advocacy efforts targeting partner institutions and particularly the leaders to ensure policies around gender equality, research misconduct and integrity, addressing bullying and harassment practices are adopted and implemented. …we all know are critical to promoting a positive research culture. [Participant 1]

ii. Ownership and buy-in by university leaders

Some participants challenged categorisation of '*ownership and buy-in by university leadership*' as a mechanism. According to two participants, '*ownership and buy-in by university leadership*' is a means to an end but not an end in itself. In other words, ownership and buy-in by university leadership is required if the RCS efforts should yield any positive change. Under the DELTAS programme, initial engagement and discussions were held with the institutional leaders to ensure that they were supportive of the programme and any changes that would be generated by the programme. Simply, ownership and buy-in by university leadership is something that needed to be achieved before the DELTAS programme was implemented and therefore deemed a contextual element. A participant said the following.

> …you'll need buy in and ownership by the leadership for the RCS activity to effectively be delivered and yield positive change. It is not an end in itself, but a means through which the desired capacity is achieved. If there's buy-in and ownership by leaders, then you can be assured that you will receive the institutional support you need to effectively implement the DELTAS research capacity building activities and any policy and systemic changes that might result from that activity. [Participant 4]

Participants also challenged the idea that equitable research partnerships would trigger *ownership and buy-in among university leaders* in contexts where there is long-term and sustainable funding, a stable political and economic environment. Participants argued that equitable partnership are important in addressing the actual capacity needs of partners and importantly leveraging each other's strengths. A participant highlighted that given the power dynamic that is characteristic of partnerships, 'ownership and buy-in by institutional leaders' is a prerequisite if power imbalances are to be challenged and call for equitable partnerships. This is captured in the excerpt below.

> …you can't talk about equity in partnership when you don't even have basic research capacity or resource base. The concept of equitable partnership has a power dynamic and mostly the established universities or institutions that have stable funding sources have the guts to question how equitable or not the partnerships are. At this point, we cannot be talking about enacting buy-in and ownership by university leaders, we are already past that. We are talking about engaging as an equal partner, being respected and trusted by partners, addressing research priorities and leveraging each other's strengths and usually the institutional leaders are at the frontline demanding for equity. [Participant 4]

iii. Institutional compliance to research policies (e.g., gender equality, research ethics and integrity and anti-harassment and anti-bullying)

In contexts where basic research capacity exists and where critical research policies have been adopted, implementation and adherence to such policies would generate progressive change in research capacity. Participants argued that in institutions where policies on gender equality, research ethics and integrity and anti-harassment and anti-bullying are actively implemented and complied, a conducive environment (context) would be created where researchers' career growth and increased retention of faculty staff can be guaranteed. One of the participants highlighted that where institutions do not comply to such research policies, faculty staff and researchers will likely be frustrated by the research practices and culture and subsequently leave the institution or research career in entirety. A participant intimated that:

> …I think the moment we start talking about increased retention of faculty staff and increased research portfolio, it means that the institution is actively implementing, complying and adhering to those research policies we talked about, that is, the gender equality, the whistleblower, research ethics and integrity, the anti-harassment and anti-bullying policies. That will create a conducive environment where faculty

> staff and researchers are enthusiastic about their research work. …the absence of that environment could mean researchers are frustrated and end up exiting the institution or the research career because no one can stand a toxic working environment. [Participant 1]

Programme Strategy/ Resource

a. Research partnerships and collaborations aimed at sharing research resource and capacity

Participants highlighted that '*research partnership between high and low capacitated institutions'* dubbed as the 'hub and spoke' model was applied in DELTAS Phase Two with the aim of promoting capacity sharing, knowledge exchange and sharing of best practices among partner institutions. The goal would be to, in the long run, improve the research capacity of the low capacitated institution. According to participants, the hub and spoke model was a programme strategy seeking to balance equity and excellence in health research. Although DELTAS Phase One was characterised by research partnerships and collaborations, these were not intentionally operationalised using the hub and spoke model. This is captured in the following excerpts.

> There's been a trend where most of the research grant applications submitted by researchers from South African universities end up securing the funding, while most of the central Africa and to some extent the West African researchers do not. When you look at that trend closely, you realise that institutions with strong capacity are able to demonstrate good research and financial management capacity, are able to put in strong proposals and obviously have strong research teams and all these is what funders are looking for when evaluating the grants applications. If the research and research funding community does not actively take appropriate actions, this trend can easily reinforce inequities. That's what DELTAS Phase Two is trying to

address through its hub and spoke model. …if we are to improve the research capacities of the less established African universities, then we need to ensure that those with improved capacity and established research systems are able to partner or collaborate with them, allowing capacity sharing to take place. Over time, we can hopefully start seeing some of the low-resourced and less established universities beginning to independently secure funding on their own and carrying out cutting edge research. [Participant 3]

…in Phase One, research partnerships and collaborations were of course seen as a way of sharing research resources and capacity among partner institutions, the only difference is that the partnerships were not intentionally operationalised using the hub and spoke model as in the Phase two of the DELTAS programme. Given the success stories reported by individuals and institutions in phase one, the learning that emerged from Phase One informed the adoption of the hub and spoke model in Phase Two. [Participant 1]

b. Advocacy for adoption of research policies

Two of the participants also highlighted that in addition to the research grants, partnerships and collaborations supporting research resource and capacity sharing among partner institutions, advocacy activities targeting Vice Chancellors, Deputy VCs and heads of research departments were carried out to ensure that changes/ improvements to the research policies could be affected. Specifically, the advocacy efforts were aimed at encouraging the university leaders to support the adaptation of their research policies to ensure they are fit for purpose and responsive to their institutional context. This is achievable in contexts where, as participants highlighted, there was buy-in and ownership by the VCs, DVCs in charge of research and departmental heads. A participant said the following.

> …under the DELTAS programme, there were advocacy and engagement activities that targeted the university VCs and DVCs in charge of research, and the aim was to promote the adoption or adaptation of relevant research policies. Obviously, this would be easier and achievable where there was buy-in and ownership by the institutional leaders as it meant that they are not only supportive of the programme but also open and ready to embrace any changes that might be brought about by the programme. [Participant 4]

c. Specialised training e.g., skills in grants writing, leadership and publication

Besides the research grants, partnerships and collaboration used as the vehicle for institutional RCS, participants highlighted that the DELTAS programme also delivered specialised training to university faculty staff and researchers. This included in areas such as grants writing, research leadership, network and collaboration development and publication. By targeting university faculty staff and researchers with specialised training and mentorship, the assumption was that the individuals would in turn use their acquired skills to develop and submit competitive research grants (which would benefit the institution through overheads fees), disseminate research outputs through publications (thus improving the institutional research profile) and expand their networks and collaborations which would – by extension – benefit their institution. A participant explained that:

> …under the CARTA programme, for instance, the specific target on junior faculty staff and researchers with specialised training and mentorship is to equip them with the skills they need to be able to write and submit competitive research grant applications, effectively lead their research initiatives, expand their research networks and widely disseminate their research outputs. I know you might ask, how is this related to university capacity. The idea is simple, institutions do benefit from overheads since most research grants contribute like 10 or 15 per cent to overheads. Regarding dissemination of research, institutions do benefit whenever their staff

publish their work because you obviously elevate the research profile of that institution. …that's the model that CARTA applies. [Participant 1]

Mechanism

i. Challenge and inspire partner- institutions' teams to adopt/adapt research policies

The participants highlighted that the interactions among partners and collaborators would allow, among other things, some of the partners to be *challenged and inspired* to adopt or adapt relevant research policies. A participant reported that some of the institutions had shared their research policies with their partner institutions and supported them to adapt the policies to their institutional context. Another participant argued that the partner-institutions' teams could also be challenged and inspired because of the active learning, knowledge exchanging and capacity sharing that happened among the partners. This is captured in the following excerpts.

> …even without using the hub and spoke model, we saw institutional teams being challenged and inspired by their partners to adopt or adapt their research policies. For instance, the [university] was challenged and inspired by University of Nairobi to adopt their research integrity policy template and even supported them to customise it. [Participant 1]

> I think the active learning, knowledge exchange and capacity sharing that happens in the context of research partnerships and collaboration obviously allows institutions to be inspired to improve their research policies. [Participant 4]

ii. Peace of mind

Participants argued that in stable political and economic environment, researchers are able to enjoy peace of mind knowing that their research activities will not be disrupted and

importantly, have reliable funding streams from the local and national governments. Overreliance on external philanthropic funding makes the research environment unpredictable and research institutions are not able to think long term in terms of research activities and this can adversely affect their ability to generate impactful research. This is captured in the excerpts below.

> …in politically unstable countries, like DRC, you can clearly see that they cannot make strides in any sector leave alone health research. I think having a stable economic and political environment is very important for your peace of mind as a researcher. It will mean, then, that you can focus on your research activities undisrupted which is something we often take for granted. To build research capacity which is a long-term exercise needs a politically and economically stable macro environment. …unfortunately, African universities and research institutions overly rely on external funding simply because the local and national governments do not have the resources, and this renders the research environment very unpredictable and unstable. [Participant 2]

> …obviously there are practical needs like renumeration, funds for procuring research equipment and all the costs related to research that requires adequate funding. So, if the research environment has stable funding and there is political conflict, you can go about your day without worrying. As a researcher, you don't want to constantly be worrying about whether you'll get your salary paid or if you will not be able to go to the lab because there's political unrest. …I know of two promising scientists who left Burkina Faso for France because of political instability, and I can say that similar cases are replicated across African countries. [Participant 4]

Outcomes

a. Adoption of relevant gender equity, research ethics and integrity and anti-harassment and anti-bullying policies

In contexts where critical research policies and procedures are non-existent, the aim of the RCS activities would be to ensure that such policies are developed and adopted. According to one of the participants, beyond adoption of the research policies, implementation of the policies and adaptation to the institutional context will see more tangible effects generated including effective redress of harassment and bullying practices and improved gender equity in research. The excerpt below captures this.

> …it makes sense to have those gender equity and anti-bullying policies adopted where they are not established. …but the adoption of those policies is just the first outcome, then there’s the lengthy process of implementing the policy and adapting it to the institutional context if we are to see improved gender representation or effective redressing of harassment or bullying vices. [Participant 3]

b. Strained departmental teaching and administrative resources

The assumption that *faculty staff record research career and portfolio growth* would *strain departmental teaching and administrative resources* was challenged by participants. The participants argued that both research and teaching should inform each other and as the research portfolio expands, the faculty staff are able to actively engage their students and expose them to new knowledge generated through research. Participants highlighted that all the DELTAS-supported researchers and faculty staff are usually capacity built in grants writing and research leadership. Importantly, research personnel costs are factored in the grant proposals thus ensuring that once the funding is secured, adequate personnel is in place to implement the research project. This is captured in the excerpts below.

> …that’s a sound assumption, but I beg to differ with the last part where you said that growth in research career and portfolio would inadvertently strain the teaching or administrative resources. The reason why protected time policies exist is to ensure

that there is adequate allocation of time for research. In reality, when the research portfolio is growing, we have observed improvement in the teaching function because the faculty staff are able to bring their research experience to bear in the classroom experience which is best practice. But the important part is that as the researcher, when you are preparing your research grant application, the finance and grants office will usually factor in all the resources you need to effectively implement your research project including the personnel. As the PI, you are able to look at the time you have at your disposal and say, I'm going to dedicate 30 percent of my time to this project. [Participant 2]

…what happens is that as the faculty staff secure additional funding, they also bring in additional staff to support the growing research portfolio. Hardly will you find a research grant that does not contribute to overheads and personnel. This is research costing 101 and all the DELTAS research fellows, their research management and finance and grants people are trained on this. [Participant 1]

Our experience is that large-scale RCS initiatives usually provide the budget base necessary for recruiting additional staff to support the expanding research portfolio. Small-scale RCS initiatives, however, are potentially more disruptive to teaching because the funding is not adequate enough to both hire additional human resource and implement RCS activities.

c. Improved research culture

Participants highlighted that the adoption, implementation and compliance to the research policies on gender equity, research conduct and integrity, whistleblowing, research pathways, anti-harassment and anti-bullying and protected time was critical for promoting a positive research culture. This is captured in the excerpts below.

> Implementation of those research policies and ensuring that the university complies with them is very important if a positive research culture is to be realised. Institutions must ensure that faculty staff are not only protected from negative vices, but they are also encouraged and motivated to carry out high quality research. [Participant 2]

> …research culture is a huge determinant in any university context and for researchers to remain enthusiastic about their work, then the university must be committed to addressing those negative practices that might affect the researcher's morale and spirit. I strongly believe that if an institution implements those policies to the letter, then we can start seeing positive culture emerging. [Participant 1]

Modification of IPTs

Based on the participants' feedback, the IPTs were modified and the final IPTs presented as summarised in the *Table 5* below.

Table 5: Modification of IPTs versus final IPTs

IPT	Modifications	Final IPTs
IPT1: If faculty staff have no protected time for research and are based in a university where there is lack or limited access to well established physical research infrastructure and equipment and no established research career pathways (C), Research grants and partnerships that support for upgrade of infrastructure and equipment, and adoption of institutional research policies such as research career pathways and protected time (resource) will likely motivate, incentivise, and empower faculty staff (reasoning) and enact ownership and buy-in among university leaders (reasoning), Resulting in the adoption of policies on protected time and research career pathways; improved physical research infrastructure and equipment (O)	Context was expanded to include gender equality, research ethics and integrity and anti-harassment and anti-bullying as critical research policies<br><br>Since ‘Ownership and buy-in by university leaders’ is needed for HRCS to yield positive results – it is changed from a mechanism to context<br><br>‘Advocacy for the adoption of institutional research policies’ was added as a resource<br><br>‘Partnership between high and low capacitated institutions’ was modified to ‘research grants, partnerships and collaborations aimed at promoting capacity sharing’ (resource)<br><br>‘Faculty staff challenged and inspired to adapt/adopt research policies’ was added as a mechanism. This is triggered in context where there is ‘ownership and buy-in by leaders’<br><br>‘Adoption of gender equality, research ethics and integrity and anti-harassment and anti-bullying’ research policies added as outcome	IPT1: If a university does not have established research-related policies including protected time, research career pathways, gender equality, whistleblower, research ethics and integrity, anti-harassment and anti-bullying (C1), has no well-established physical research infrastructure and equipment (C2) but there is ownership and buy-in among university leaders (C3), research grants, partnerships and collaborations that support for upgrade of infrastructure and equipment, and advocacy efforts that promote adoption of institutional research policies (resource) will likely motivate, incentivise, and empower faculty staff (reasoning) and challenge/inspire faculty staff (reasoning), resulting in the adoption of relevant research policies (O1) and improved physical research infrastructure and equipment (O2).
IPT2 If faculty staff have protected time for research and are based in a university where there is access to well established physical research infrastructure and equipment and established research career pathways (C), Research partnerships between highly capacitated and low capacitated universities/ research institutions that allow sharing of resources such as financial, technical expertise and infrastructure (resource) will	Context was expanded to include ‘compliant to relevant research policies (e.g., protected time, gender equality, research ethics and integrity and anti-harassment and anti-bullying)’	IPT2: If a university is compliant to research-related policies (e.g., protected time, research career pathways, gender equality, whistleblower, research ethics and integrity, anti-harassment and anti-bullying) (C1), has well-established physical research infrastructure and equipment (C2), research grants, partnerships and collaborations that

likely motivate, incentivise, and empower them (reasoning), Faculty staff record growth in their research career and portfolio, but this may strain their departmental teaching and administrative resources (O)<br><br>IPT3: If faculty staff have protected time for research, access to well established physical research infrastructure and equipment and based in an institution with established institutional research policies, systems, and culture (C), research partnerships between highly capacitated and low capacitated universities/ research institutions that allow sharing of resources such as financial, technical expertise and infrastructure and provide mentorship for emerging researchers (resource) will likely motivate, incentivise, and empower, and enact commitment and enthusiasm among junior and senior faculty staff (reasoning), resulting in increased retention of faculty staff; increased research portfolio including research funding, and generation of impactful research that promotes positive health outcomes and policymaking (O)	'Ownership and buy-in by university leaders' is needed for HRCS to yield positive outcomes – therefore it is added as context<br><br>'Partnership between high and low capacitated institutions' was modified to 'research grants, partnerships and collaborations aimed at promoting capacity sharing' (resource)<br><br>Outcome was expanded to include 'improved research culture'<br><br>Outcome was modified to exclude 'strain in departmental teaching and administrative resources' as an unintended negative outcome.<br><br>Following the exclusion of the outcome, IPT2 and 3 were consequently merged because the contexts and mechanism were similar.	support resource sharing, and specialised training and mentorship for emerging researchers (resource) will likely motivate, incentivise, and empower faculty staff (reasoning), resulting in increased retention of faculty staff (O1), increased research portfolio including research funding (O2), improved research culture (O3) and generation of impactful research that promotes positive health outcomes and policymaking (O4)
IPT4: If a university has established equitable strategic research partnerships, has access to sustainable long-term funding and is based in a stable political and economic environment (C), Research partnerships between highly capacitated and low capacitated universities/ research institutions that allow sharing of resources such as financial, technical expertise and infrastructure (resource) will likely trigger ownership and buy-in among university leaders, and trust and respect among staff (reasoning), Research partnerships and research management and governance systems strengthened and generation of impactful research that promotes positive health outcomes and policymaking (O)	'Ownership and buy-in by university leaders' is needed for HRCS to yield positive outcomes – therefore it is changed from a mechanism to context<br><br>Mechanism modified to include 'peace of mind for researchers' which is triggered in stable political and economic environments<br><br>'Partnership between high and low capacitated institutions' was modified to 'research grants, partnerships and collaborations aimed at promoting capacity sharing' (resource)	IPT3: If a university has established equitable strategic research partnerships (C1), has access to sustainable long-term funding (C2) and is based in a stable political and economic environment (C3), research grants, partnerships and collaborations that allow sharing of resources such as financial, technical expertise and infrastructure (resource) will likely trigger trust and respect, and peace of mind among partners/ staff and (reasoning), resulting in strengthened research partnerships, management and governance systems (O1) and generation of impactful research that promotes positive health outcomes and policymaking (O2).

Discussion

This synthesis paper has elicited three IPTs representing a diverse range of concepts crucial to the implementation and effectiveness of HRCS programmes implemented in African universities. The IPTs describe HRCS of African universities at different stages of research capacity. For instance, IPT1 describes a university context where research capacity is almost non-existent and no relevant research policies and procedures are in place. In such a context, the goal of HRCS interventions would be to build research capacity from scratch, for instance, adoption of research policies, development of research infrastructure and acquisition of research equipment. IPT2 and IPT3 describe university contexts where basic research capacity exists. In such contexts, the goal of HRCS interventions would be to strengthen the existing institutional research systems in order to achieve more long-term research capacity outcomes such as retention of faculty/researchers, growth in research career and portfolio, increased research funding, improved research culture, and generation of impactful health research that has positive effects on people’s health and policymaking. This distinction between IPT1 (capacity building) and IPT2/3 (capacity strengthening) is consistent with previous attempts by Dean et al. (66) to distinguish between the terms capacity building and capacity strengthening.

Since many HRCS initiatives implemented in LMICs (including the DELTAS Africa programme) are delivered based on research grants, partnerships and collaborations (67), the IPTs in this synthesis were therefore framed using ‘research grants, partnerships and collaborations’ as the programme strategy. The research grants, partnerships and collaborations foster the sharing of research funding, expertise, equipment and facilities and best practices among faculty/researchers and their institutions (67). Some of the direct investments go towards upgrading research infrastructure and purchase of research equipment and reagents (10, 36), while majority of the HRCS strategies focus on individuals (university leaders, researchers/ faculty, etc.) working in the universities. For instance, the HRCS initiative may target university leaders (through advocacy efforts) to ensure they are

supportive of the RCS interventions and buy-in to any research policy changes that come with such interventions (40), the faculty/ researchers by empowering them with technical and soft skills to become agents of change within their institutions (37, 42), or research management, finance, and grants management support staff whose improved capacity could consequently improve the research , finance, and grants management practices (64) (68).

The evidence synthesised in this article suggests that, for HRCS interventions to be more effective in strengthening research capacity in African universities, contextual challenges (e.g., lack of funding for health research, ineffective research policies or lack thereof, lack of institutional support for research and poor research culture, etc.) need to be addressed (16, 48) and the university staff empowered, incentivised and motivated (47, 50). A systems approach to HRCS should be used to deliver sustainable impact (69), and by doing so, provide an enabling and conducive research environment where researchers, research and research users can thrive. For instance, the implementation and adherence to research policies (e.g., protected time, research career pathways, among others), and fostering a positive research culture (16, 56) guarantees a university environment that is conducive to research. Adherence to research policies will ensure that faculty/ researchers are protected against negative vices (e.g., bullying, harassment, gender bias) by ensuring that appropriate channels for reporting and addressing such vices are in place (70). Importantly, adherence to research policies such as research ethics and integrity would promote good research practices which are critical for promoting people-centred research that has real impact to policymaking and population health outcomes (71).

The IPTs depict that multiple contexts are necessary for one or more mechanism to be triggered thus generating one or more research capacity outcomes. For instance, effective implementation and compliance to a career pathway/promotion policy can motivate faculty to grow in their research career, but a friendly research culture that is free from bullying and harassment can motivate them to stay (retention of faculty). Staff retention is a key issue that

affects long-term HRCS efforts and which require improvements in research capacity and opportunities at systems level and follow-on funding (72). It is challenging to achieve this [staff retention] in LMICs given the challenging environment characterised by a limited funding base which affects almost every other aspect (39, 72). Notably, an equitable research partnership will enact trust and respect among researchers/ faculty thus strengthening the partnership further (46) and allowing impactful research to be generated since the partnership will be [anchored on respect] addressing local priorities and respecting local knowledge (41, 43, 46). The primary question is whether equitable partnerships can be achieved by African universities when there is so much reliance on external funding from Global North entities (16, 41, 53, 64). To achieve the long-term research capacity outcomes, the university staff/ researchers will also need a stable political and economic environment with adequate research funding which will both incentivise them and provide peace of mind. For instance, adequate funding may guarantee them of decent remuneration (thus meeting their personal needs) and facilitate the procurement of critical research equipment and reagents, and the stable political environment will mean that the research activities will go undisrupted. Undoubtedly, African governments need to invest and sustain long-term funding towards health research (thus reducing reliance on external funding) and providing a stable political and economic environment where HRCS efforts can yield sustainable impact (73).

The evidence identifies several HRCS outcomes which include improved research infrastructure, increased research funding, research career and portfolio growth, staff retention, improved research policies and culture, improved research management and governance, strengthened research partnerships and generation of impactful research (10, 16, 37-39, 53, 59, 61, 62). Although HRCS initiatives are usually designed to deliver desired positive capacity outcomes, given the complexity of those initiatives and the environment within which they are implemented, unintended negative outcomes might be generated in ways that may not be anticipated. For instance, one of the papers reviewed in this synthesis

highlighted that the expansion of the research portfolio in a university could inadvertently affect the teaching function by taking away (stretching) the available teaching resources (52) which is an unintended negative outcome. However, the DELTAS Africa programme designers argued that as the research portfolio expands, ideally, the teaching function and activities get better because the research evidence is brought to bear with the classroom teaching activities. This is practicable, from their perspective, because the DELTAS Africa programme is a large-scale initiative that provides the budget base necessary for recruiting additional staff to support the expanding research portfolio. However, our experience is that small-scale HRCS initiatives are potentially more disruptive to teaching because the funding is inadequate to both hire additional human resource and implement RCS activities.

This synthesis was not free of challenges. The major challenge emanated from the fact that none of the papers included in the literature review had presented findings/arguments using the CMO framing. Our first and last authors' [MNM and JP] professional hunches and experiences in HRCS, particularly in the African context, were helpful in making the linkages between the C, M and Os. The second author's [AH] realist expertise helped to ensure that the articulation of the IPTs was in line with the realist philosophy. Since the synthesis process was iterative, discussions among the co-authors helped to tweak the CMOs multiple times until consensus was reached. Importantly, it is clear, given the long chain of CMOs that multiple contexts are required for one or more mechanism to be triggered to generate one or more outcomes. As demonstrated in this article, the inability to link a single outcome to a single mechanism and context explains the multifaceted and complex nature of the HRCS initiatives. The high level of abstraction of the theories applied helped to generate few overarching but transferable programme theories, and consequently this helped to avoid generation of overabundance of IPTs as guided by Shearn et al (74). For instance, instead of focusing on the mechanism triggered by the 'implementation and adherence to anti-bullying and anti-harassing policies' in particular, the focus remained on the 'research policies that foster a conducive research environment/ culture.' The former would have

looked at each policy provision(s) as a context and demand eliciting a mechanism for each policy provision(s); this would have probably generated an unmanageable number of IPTs.

This is, to our knowledge, the first fully fledged realist synthesis that has explored how HRCS interventions work to strengthen research capacities of African universities. The only other published study is a 'light-touch' realist evaluation by Marjanovic et al (75). These transferrable and overarching IPTs can be applied across different university settings in Africa and potentially other LMIC regions, to inform the design, implementation, and evaluation of HRCS interventions thus ensuring that they can drive sustainable impact. Besides advancing the institutional HRCS discourse and related CMOs, this article also makes a methodological contribution. There are few articles that provide practical guidance on how to develop robust IPTs and this paper addresses that gap specifically in the HRCS context.

Conclusion

Institutional HRCS is undoubtedly a complex and multifaceted activity that involves intervening at different levels (individual, institutional and systems) to generate research capacity outcomes which are moderated by multiple contextual aspects. The value of complexity-aware approaches like realist evaluation in understanding how HRCS interventions work in diverse African university settings cannot be overstated. This article sheds light on the critical mechanisms, contexts, and outcomes that could drive health research capacity at African universities. The insights presented in this article can guide future HRCS evaluations and help to establish a clearer understanding of the generative mechanisms underpinning effective institutional-level research capacity strengthening interventions. To drive sustainable improvements in health research capacity at African universities, there is need to intervene at individual, institutional and policy levels with the aim of generating systemic changes.

The IPTs presented in this article will be tested through a primary realist evaluation covering the DELTAS Africa Programme (Phase I) participants. Based on the emerging evidence, the IPTs will be confirmed, refuted or refined, and new programme theories potentially identified. The realist evaluation will, therefore, expand on the CMO concepts described in this article and provide insights into the application of realist evaluations in HRCS settings.

Ethics Statement

Ethics approval for this study was granted by both the Liverpool School of Tropical Medicine, UK, and Strathmore University, Kenya, Research Ethics Committees as part of a wider realist study.

Competing Interests

The authors have declared that no competing interests exist.

Funding source(s)

This work has been funded by the LSTM-Lancaster Medical Research Council (MRC) Translational and Quantitative Skills Doctoral Training Partnership (DTP) Award.

References

1. Lansang MA, Dennis R. Building capacity in health research in the developing world. Bulletin of the World Health Organization. 2004;82(10):764-70.
2. Vasquez EE, Hirsch JS, Giang LM, Parker RG. Rethinking health research capacity strengthening. Global public health. 2013;8(sup1):S104-S24.
3. The Academy of Medical Sciences. Strengthening clinical research capacity in low- and middle-income countries. 2017.
4. Morel T, Maher D, Nyirenda T, Olesen OF. Strengthening health research capacity in sub-Saharan Africa: mapping the 2012–2017 landscape of externally funded international postgraduate training at institutions in the region. Globalization and health. 2018;14(1):1-10.
5. Enoch J, Thornton I. Health research capacity strengthening: a UKCDS mapping. UKCDS; 2014.
6. Pulford J, Crossman S, Abomo P, Quach JA, Begg S, Ding Y, et al. Guidance and conceptual tools to inform the design, selection and evaluation of research capacity strengthening interventions. BMJ Global Health. 2021;6(3):e005153.
7. Khisa AM, Gitau E, Pulford J, Bates I. A framework and indicators to improve research capacity strengthening evaluation practice. 2019.
8. Hanney SR, Kanya L, Pokhrel S, Jones TH, Boaz A. How to strengthen a health research system: WHO's review, whose literature and who is providing leadership? Health Research Policy and Systems. 2020;18(1):72.
9. Hanney S, Kuruvilla S, Soper B, Mays N. Who needs what from a national health research system: lessons from reforms to the English Department of Health's R&D system. Health Research Policy and Systems. 2010;8(1):1-23.
10. Begg S, Wright A, Small G, Abdoulaye D, Kisinza W, Koudou B, et al. Ripple effects of research capacity strengthening: a study of the effects of a project to support test facilities in three African countries towards Good Laboratory Practice certification. Gates Open Research. 2020;4.

11. Cole DC, Boyd A, Aslanyan G, Bates I. Indicators for tracking programmes to strengthen health research capacity in lower-and middle-income countries: a qualitative synthesis. Health research policy and systems. 2014;12(1):1-13.

12. Bates I, Boyd A, Aslanyan G, Cole DC. Tackling the tensions in evaluating capacity strengthening for health research in low-and middle-income countries. Health policy and planning. 2015;30(3):334-44.

13. Bates I, Boyd A, Smith H, Cole DC. A practical and systematic approach to organisational capacity strengthening for research in the health sector in Africa. Health research policy and systems. 2014;12(1):1-10.

14. Vicente-Crespo M, Agunbiade O, Eyers J, Thorogood M, Fonn S. Institutionalizing research capacity strengthening in LMICs: A systematic review and meta-synthesis. AAS Open Research. 2020;3.

15. Buser JM, Capellari E, Wondafrash M, Gray R, Morris KL, Jacobson-Davies FE, et al. Unravelling the complexity of research capacity strengthening for health professionals in low-and middle-income countries: A concept analysis. Journal of Advanced Nursing. 2024;00:1–15.

16. Kasprowicz VO, Chopera D, Waddilove KD, Brockman MA, Gilmour J, Hunter E, et al. African-led health research and capacity building-is it working? BMC Public Health. 2020;20:1-10.

17. Jones CM, Sobngwi-Tambekou J, Mijumbi RM, Hedquist A, Wenham C, Parkhurst J. The roles of regional organisations in strengthening health research systems in Africa: activities, gaps, and future perspectives. International Journal of Health Policy and Management. 2022;11(11):2672-85.

18. Boyd A, Cole DC, Cho D-B, Aslanyan G, Bates I. Frameworks for evaluating health research capacity strengthening: a qualitative study. Health research policy and systems. 2013;11(1):1-11.

19. Bates I, editor Designing and measuring the progress and impact of health research capacity strengthening initiatives. BMC proceedings; 2015: Springer.

20. Pawson R, Tilley N. Realistic evaluation: Sage; 1997.

21. Pawson R, Tilley N. Realistic Evaluation Bloodlines. American Journal of Evaluation. 2001;22(3):317-24.

22. Dalkin SM, Greenhalgh J, Jones D, Cunningham B, Lhussier M. What's in a mechanism? Development of a key concept in realist evaluation. Implementation science. 2015;10(1):1-7.

23. Mutua MN, Harding A, Pulford J. Developing the Initial Programme Theories on Health Research Capacity Strengthening of African Universities: A Realist Synthesis Protocol. International Journal of Qualitative Methods. 2024;23:16094069241260138.

24. Pawson R, Greenhalgh T, Harvey G, Walshe K. Realist review-a new method of systematic review designed for complex policy interventions. Journal of health services research & policy. 2005;10(1_suppl):21-34.

25. Pawson R. Evidence-based policy: a realist perspective: Sage; 2006.

26. Astbury B. Some reflections on Pawson's science of evaluation: A realist manifesto. Evaluation. 2013;19(4):383-401.

27. Molitor V, Busse TS, Giehl C, Lauer R, Otte IC, Vollmar HC, et al. How and why educational interventions work to increase knowledge of delirium among healthcare professionals in nursing homes: a protocol for a realist review. BMJ Open. 2023;13(7):e072591.

28. Husk K, Blockley K, Lovell R, Bethel A, Lang I, Byng R, et al. What approaches to social prescribing work, for whom, and in what circumstances? A realist review. Health & Social Care in the Community. 2020;28(2):309-24.

29. Kastner M, Bhattacharyya O, Hayden L, Makarski J, Estey E, Durocher L, et al. Guideline uptake is influenced by six implementability domains for creating and communicating guidelines: a realist review. Journal of Clinical Epidemiology. 2015;68(5):498-509.

30. McCormack B, Rycroft-Malone J, DeCorby K, Hutchinson AM, Bucknall T, Kent B, et al. A realist review of interventions and strategies to promote evidence-informed healthcare: a focus on change agency. Implementation Science. 2013;8(1):107.

31. Molitor V, Busse TS, Giehl C, Lauer R, Otte IC, Vollmar HC, et al. Supplemental material - Realist Synthesis Appraisal Form. BMJ Open. 2023;13(7):e072591.

32. Mukumbang FC, Van Belle S, Marchal B, van Wyk B. An exploration of group-based HIV/AIDS treatment and care models in Sub-Saharan Africa using a realist evaluation (Intervention-Context-Actor-Mechanism-Outcome) heuristic tool: a systematic review. Implementation Science. 2017;12(1):107.

33. Fryer T. A critical realist approach to thematic analysis: producing causal explanations. Journal of Critical Realism. 2022;21(4):365-84.

34. Wiltshire G, Ronkainen N. A realist approach to thematic analysis: making sense of qualitative data through experiential, inferential and dispositional themes. Journal of Critical Realism. 2021;20(2):159-80.

35. Page MJ, McKenzie JE, Bossuyt PM, Boutron I, Hoffmann TC, Mulrow CD, et al. The PRISMA 2020 statement: an updated guideline for reporting systematic reviews. BMJ. 2021;372:n71.

36. Manabe YC, Katabira E, Brough Richard L, Coutinho Alex G, Sewankambo N, Merry C. Developing independent investigators for clinical research relevant for Africa. Health Research Policy and Systems. 2011;9:44-.

37. Akuffo H, Freeman P, Johansson E, Obua C, Ogwal-Okeng J, Waako P. Doctoral education and institutional research capacity strengthening: An example at Makerere University in Uganda (2000–2013). Higher Education Policy. 2014;27:195-217.

38. Elmusharaf K, Tahir H, O'Donovan D, Brugha R, Homeida M, Abbas AM, et al. From local to global: a qualitative review of the multi-leveled impact of a multi-country health research capacity development partnership on maternal health in Sudan. Globalization and health. 2016;12:1-14.

39. Mathai M, Stoep AV, Kumar M, Kuria M, Obondo A, Kimani V, et al. Building mental health research capacity in Kenya: a South-North collaboration. Global Social Welfare. 2019;6:177-88.

40. Marjanovic S, Hanlin R, Diepeveen S, Chataway J. Research capacity building in Africa: Networks, institutions and local ownership. Journal of International Development. 2013;25(7):936-46.

41. Ikomey GM, Chendi BH, Ngah YE, Mbanyamsig MY, Mesembe M, Emilia L, et al. Development of a south-south clinical research capacity strengthening: institutional partnership platform between Cameroon and South Africa. Texila International Journal of Public Health. 2023;11(1):48-60.

42. Perier C, Nasinghe E, Charles I, Ssetaba LJ, Ahyong V, Bangs D, et al. Workshop-based learning and networking: a scalable model for research capacity strengthening in low- and middle-income countries. Global health action. 2022;15:2062175.

43. Noormahomed EV, Mocumbi AO, Preziosi M, Damasceno A, Bickler S, Smith DM, et al. Strengthening research capacity through the medical education partnership initiative: the Mozambique experience. Human Resources for Health. 2013;11.

44. Torondel B, Balls E, Chisenga CC, Kumwenda S, Okello E, Simiyu S, et al. Experiences of capacity strengthening in sanitation and hygiene research in Africa and Asia: the SHARE Research Consortium. Health Research Policy and Systems. 2019;17(77):1-10.

45. Mremi A, Pancras G, Mrema D, Morris B, Mwakyandile T, Msanga D, R., et al. Mentorship of young researchers in resource-limited settings: experiences of the mentees from selected health sciences Universities in Tanzania. BMC Medical Education. 2023;23(375):1-7.

46. Dean L, Njelesani J, Smith H, Bates I. Promoting sustainable research partnerships: a mixed-method evaluation of a United Kingdom–Africa capacity strengthening award scheme. Health Research Policy and Systems. 2015;13(81):1-10.

47. Daniels J, Nduati R, Kiarie J, Farquhar C. Supporting early career health investigators in Kenya: a qualitative study of HIV/AIDS research capacity building. Pan African Medical Journal. 2015;20:192.

48. Izugbara CO, Kabiru CW, Amendah D, Dimbuene ZT, Donfouet HPP, Atake EH, et al. "It takes more than a fellowship program": reflections on capacity strengthening for health systems research in sub-Saharan Africa. BMC Health Serv Res. 2017;17(Suppl 2):696.

49. Bates I, Taegtmeyer M, Squire SB, Ansong D, Nhlema-Simwaka B, Baba A, et al. Indicators of sustainable capacity building for health research: analysis of four African case studies. Health Research Policy and Systems. 2011;9(1):1-9.

50. Johnson BJ, Louw AH. Building a research culture from scratch at a university of technology. Mediterranean Journal of Social Sciences. 2014;5(1).

51. Singh RJ. Challenges and successes of research capacity building at a rural South African university. South African Journal of Higher Education. 2015;29(3):183-200.

52. Sewankambo N, Tumwine JK, Tomson G, Obua C, Bwanga F, Waiswa P, et al. Enabling dynamic partnerships through joint degrees between low-and high-income countries for capacity development in global health research: experience from the Karolinska Institutet/Makerere University partnership. PLoS medicine. 2015;12(2):e1001784.

53. Aiyenigba A, Abomo P, Wiltgen Georgi N, Bates I, Pulford J. Enabling research capacity strengthening within a consortium context: A qualitative study. BMJ Global Health: BMJ Group; 2022.

54. Noormahomed EV, Mocumbi AO, Ismail M, Carrilho C, Patel S, Nguenha A, et al. The medical education partnership initiative effect on increasing health professions education and research capacity in Mozambique. Annals of global health. 2018;84(1):47.

55. Wakida EK, Atuhaire CD, Karungi CK, Maling S, Obua C. Mbarara University Research Training Initiative: Experiences and Accomplishments of the MEPI Junior D43 TW010128 Award in Uganda. Advances in Medical Education and Practice. 2021:1397-410.

56. Mashaah T, Hakim J, Chidzonga M, Kangwende RA, Naik Y, Federspiel N, et al. Strengthening research governance for sustainable research: experiences from three Zimbabwean universities. Acad Med. 2014;89(8 Suppl):S69-72.

57. Dalmar AA, Hussein AS, Walhad SA, Ibrahim AO, Abdi AA, Ali MK, et al. Rebuilding research capacity in fragile states: the case of a Somali–Swedish global health initiative. Global Health Action. 2017;10(1):1348693.

58. Maponga CC, Mhazo AT, Morse GD. A framework for sustainable capacity-building for collaborative North–South translational health research and training in a resource-constrained setting. Health Research Policy and Systems. 2023;21(1):24.

59. Hakim JG, Barry M, Matenga J, Cowan F, Campbell TB. Medical Education Partnership Initiative in Zimbabwe: partnerships for transformation. The Lancet Global Health. 2017;5(2):e143-e4.

60. Mutale W, Nzala S, Cassell HM, Martin MH, Chi BH, Mukanu M, et al. HIV research training partnership of the University of Zambia and Vanderbilt University: Features and early outcomes. Annals of Global Health. 2019;85(1).

61. Ponka D, Coffman M, Fraser-Barclay KE, Fortier RD, Howe A, Kidd M, et al. Fostering global primary care research: a capacity-building approach. BMJ global health. 2020;5(7):e002470.

62. Langhaug L, F., Jack H, Hanlon C, Holzer S, Sorsdahl K, Mutedzi B, et al. 'We need more big trees as well as the grass roots': going beyond research capacity building to develop sustainable careers in mental health research in African countries. International Journal of Mental Health Systems. 2020;14:1-14.

63. Wallis S, Cole DC, Gaye O, Mmbaga BT, Mwapasa V, Tagbor H, et al. Qualitative study to develop processes and tools for the assessment and tracking of African institutions' capacity for operational health research. BMJ open. 2017;7(9):e016660.

64. Pulford J, Crossman S, Begg S, Amegee Quach J, Abomo P, El Hajj T, et al. Strengthening research management and support services in sub-Saharan African universities and research institutions. AAS open research. 2020;3:31.

65. Bates I, Boyd A, Smith H, Cole DC. A practical and systematic approach to organisational capacity strengthening for research in the health sector in Africa. Health Research Policy and Systems: BioMed Central; 2014.

66. Dean L, Gregorius S, Bates I, Pulford J. Advancing the science of health research capacity strengthening in low-income and middle-income countries: a scoping review of the published literature, 2000-2016. BMJ OPEN. 2017;7(12):e018718.

67. Franzen SR, Chandler C, Lang T. Health research capacity development in low and middle income countries: reality or rhetoric? A systematic meta-narrative review of the qualitative literature. BMJ open. 2017;7(1):e012332.

68. Harste HJ, Kiff G, Okeke IN, Adebiyi AO, Ravikumar KL, Nagaraj G, et al. Good Financial Grant Practice: A Tool for Developing and Demonstrating Institutional Financial and Grant Management Capacity in Global Health. Clinical Infectious Diseases. 2021;73(Suppl_4):S275-s82.

69. Malekzadeh A, Michels K, Wolfman C, Anand N, Sturke R. Strengthening research capacity in LMICs to address the global NCD burden. Glob Health Action. 2020;13(1):1846904.

70. Täuber S, Oliveri NF, Kostakopoulou D, Mahmoudi M. Breaking the silence around academic harassment. Wiley Online Library; 2022. p. 2337-44.

71. Aubert Bonn N, Godecharle S, Dierickx K. European universities’ guidance on research integrity and misconduct: Accessibility, approaches, and content. Journal of Empirical Research on Human Research Ethics. 2017;12(1):33-44.

72. Hanka S. Research capacity strengthening–Learning brief. NIHR Open Res. 2024;4(61):61.

73. Kokwaro G, Kariuki S. Medical research in Africa: problems and some solutions. Malawi medical journal. 2001;13(3):40.

74. Shearn K, Allmark P, Piercy H, Hirst J. Building realist program theory for large complex and messy interventions. International Journal of Qualitative Methods. 2017;16(1):1-16.

75. Marjanovic S, Cochrane G, Robin E, Sewankambo N, Ezeh A, Nyirenda M, et al. Evaluating a complex research capacity-building intervention: Reflections on an evaluation of the African Institutions Initiative. Evaluation. 2017;23(1):80-101.

---

[i] Funded by the Wellcome and the UK Department for International Development (currently the Foreign Commonwealth and Development Office), the DELTAS Africa programme Phase 1 (2015-2021) supported eleven (11) research consortia led by distinguished African principal investigators. The programme was delivered by the Alliance for Accelerating Excellence in Science in Africa (AESA) based at the African Academy of Science (AAS). One of the primary objectives of the programme was to strengthen the research capacities of individuals and their research institutions to deliver – in the long run – cutting edge and impactful health research.

PRISMA Checklist

Click here to access/download
Supporting Information
PRISMA Checklist.docx

Click here to access/download
Supporting Information
Realist Appraisal Form_extracted data.docx

Click here to access/download
Supporting Information
Realist Review Data Extraction - summary of included papers.xlsx
