# Supplementary material for: Eliciting initial programme theories for a health research capacity strengthening initiative targeting African universities: A realist synthesis
Source: PLoS One. 2025 Aug 20;20(8):e0330360. doi: 10.1371/journal.pone.0330360 (PMC12367166; doi:10.1371/journal.pone.0330360)
Supplement: Attachment — Submitted filename: Response to Reviewers.docx [file pone.0330360.s007.docx]

**RESPONSE TO REVIEWERS**

**Round 2 Editor’s Comments**

| **No.** | **Comments** | **Response** |
| --- | --- | --- |
| 1 | "We note that there is identifying data in the Supporting Information file <S2_Included Papers.xlsx>. Due to the inclusion of these potentially identifying data, we have removed this file from your file inventory. Prior to sharing human research participant data, authors should consult with an ethics committee to ensure data are shared in accordance with participant consent and all applicable local laws.  Data sharing should never compromise participant privacy. It is therefore not appropriate to publicly share personally identifiable data on human research participants. The following are examples of data that should not be shared:  - Name, initials, physical address - Ages more specific than whole numbers - Internet protocol (IP) address - Specific dates (birth dates, death dates, examination dates, etc.) - Contact information such as phone number or email address - Location data - ID numbers that seem specific (long numbers, include initials, titled “Hospital ID”) rather than random (small numbers in numerical order)  Data that are not directly identifying may also be inappropriate to share, as in combination they can become identifying. For example, data collected from a small group of participants, vulnerable populations, or private groups should not be shared if they involve indirect identifiers (such as sex, ethnicity, location, etc.) that may risk the identification of study participants.  Please remove or anonymize all personal information (<NAMES>), ensure that the data shared are in accordance with participant consent, and re-upload a fully anonymized data set. Please note that spreadsheet columns with personal information must be removed and not hidden as all hidden columns will appear in the published file. | All the data has been appropriately anonymised/ pseudonymised in the Supplementary Material (now labelled as *S3_List of Included Papers)* |
| 2 | Please amend the title either on the online submission form or in your manuscript so that they are identical.  In your online submission form your title stated as follows:  "Theorising health research capacity strengthening of African universities: A realist synthesis"  However, in your manuscript it stated as follows:  "Eliciting initial programme theories for a health research capacity strengthening initiative targeting African universities: A realist synthesis” | The online submission form has been edited to capture "Eliciting initial programme theories for a health research capacity strengthening initiative targeting African universities: A realist synthesis” as the title. |
| 3 | Please amend the manuscript submission data (via Edit Submission) to include author Ferdinand C. Mukumbang. | The submission data has been amended to include Ferdinand C. Mukumbang as a co-author |
| 4 | In the PRISMA flowchart, you specify that originally 1241 records were screened for this study. Please provide a numbered table of all those 1241 studies identified in the literature search, including those that were excluded from the analyses. For every excluded study, the table should list the reason(s) for exclusion. If any of the included studies are unpublished, include a link (URL) to the primary source. An example for how we would expect a table like this to look like can be found here: <https://journals.plos.org/plosone/article?id=10.1371/journal.pone.0313866#sec017> (S2 file)  2) Please provide a table showing the completed risk of bias and quality/certainty assessments for each study or outcome. | There was a mix up in the numbers captured in the initial PRISMA flowchart (as it included records for a separate review study). This has now been corrected, and an amended flowchart has been included in manuscript. The actual records screened in this review were 414 instead of 1241. Please check the *S1_Full List of screened records*. We have run the search again just to verify the records.  The PRISMA 2020 Checklist has also been updated to capture the revisions. This has been added as Supplementary Material 4 (*S4_The PRISMA Checklist*) |
|  | **Round 1 Comments from Editor** |  |
| 1 | Please ensure that your manuscript meets PLOS ONE's style requirements, including those for file naming. | The manuscript has been revised to include Introduction, Methods, Findings, Discussion and Conclusion sections.  Tables and Figures have been clearly captioned and in-text citation correctly done. |
| 2 | Please note that funding information should not appear in any section or other areas of your manuscript. We will only publish funding information present in the Funding Statement section of the online submission form. Please remove any funding-related text from the manuscript. | The funding statement has been removed from the manuscript and added in the cover letter |
| 3 | Thank you for stating the following financial disclosure:  “Medical Research Council (MRC) Doctoral Training Award”  Please state what role the funders took in the study. If the funders had no role, please state: "The funders had no role in study design, data collection and analysis, decision to publish, or preparation of the manuscript."  If this statement is not correct you must amend it as needed.  Please include this amended Role of Funder statement in your cover letter; we will change the online submission form on your behalf. | The statement "The funders had no role in study design, data collection and analysis, decision to publish, or preparation of the manuscript” has been added in the Cover Letter  The updated Cover Letter has been uploaded |
| 4 | We note that you have indicated that there are restrictions to data sharing for this study. PLOS only allows data to be available upon request if there are legal or ethical restrictions on sharing data publicly. For more information on unacceptable data access restrictions, please see <http://journals.plos.org/plosone/s/data-availability#loc-unacceptable-data-access-restrictions>.  Before we proceed with your manuscript, please address the following prompts:  a) If there are ethical or legal restrictions on sharing a de-identified data set, please explain them in detail (e.g., data contain potentially identifying or sensitive patient information, data are owned by a third-party organization, etc.) and who has imposed them (e.g., a Research Ethics Committee or Institutional Review Board, etc.). Please also provide contact information for a data access committee, ethics committee, or other institutional body to which data requests may be sent.  b) If there are no restrictions, please upload the minimal anonymized data set necessary to replicate your study findings to a stable, public repository and provide us with the relevant URLs, DOIs, or accession numbers. For a list of recommended repositories, please see  https://journals.plos.org/plosone/s/recommended-repositories. You also have the option of uploading the data as Supporting Information files, but we would recommend depositing data directly to a data repository if possible.  We will update your Data Availability statement on your behalf to reflect the information you provide. | The partners’ interview transcripts (data) have been appropriately anonymised/ pseudonymised and uploaded onto the Figshare data repository.  See the link below: <https://figshare.com/s/6e3cde070a35b14a9086> |
| 5 | Please include your full ethics statement in the ‘Methods’ section of your manuscript file. In your statement, please include the full name of the IRB or ethics committee who approved or waived your study, as well as whether or not you obtained informed written or verbal consent. If consent was waived for your study, please include this information in your statement as well. | Ethics statement has been included in the Methods section  The ethics statement initially included at the end of the manuscript has been removed |
| 6 | We note you have included a table to which you do not refer in the text of your manuscript. Please ensure that you refer to Table 1 and 2 in your text; if accepted, production will need this reference to link the reader to the Table. | All Tables are now cited in the text |
| 7 | Please include captions for your Supporting Information files at the end of your manuscript, and update any in-text citations to match accordingly. Please see our Supporting Information guidelines for more information: <http://journals.plos.org/plosone/s/supporting-information> | Captions for all the Supporting Information files have been included at the end of the manuscript, and in-text citations also included |
|  | **Reviewer #1 Comments** |  |
| 9 | Thank you for the opportunity to review this paper. This is an important topic & this paper is a good one in its field. I have made some comments on the manuscript that I hope can strengthen the quality of the paper:  Overall, I found the paper difficult to follow. I suggest the authors scale back the questions and analysis completed in this paper to provide more space to introduce the concepts. Hence, findings should be modified and structured based on the questions of the research. | The paper has been restructured. The Methods section clearly describes the 2 stages i.e., literature review and interviews with partners.  The findings section, particularly Phase 2 section on partners’ interviews has been modified to focus on each programme theory instead of separately describing all the C, M and O elements identified from the data.  The article explicitly shows how the IPTs were gleaned from the literature review and refined through partners’ interviews. |
| 10 | Keywords should be modified base on MeSH. | Keywords included |
| 11 | As it mentioned in page 8 line 197 thematic analysis was applied in this research but it is not any table in the body of article to show main Themes and Subthemes of finding of this review. | The identified themes have been presented/ described under the contexts, mechanism and outcome subheadings. |
| 12 | Limitations of the study is not mentioned in the paper. | The limitations of the study have been described in the Discussion section (see 2 paragraphs before the last) |
|  | **Reviewer #2 Comments** |  |
| 13 | The paper is an important contribution to the research capacity strengthening efforts. It has effectively used realist evaluation framework.  However, the paper needs to be revised and made concise significantly. The paper is difficult to read in its current format. Many redundant information needs to be removed. The findings are presented in present tense and therefore, reads like a discussion point. The sentence structure needs to be changed in several places throughout the paper; I have not highlighted every place which needs this change. Some of the domains parsed out in the findings can also be lumped together. This can perhaps help to eliminate redundancy. | The paper has been restructured. The Methods section clearly describes the 2 stages i.e., review and realist interviews with partners.  The findings section, particularly Phase 2 section on partners’ interviews has been modified to focus on each programme theory instead of separately describing all the elements identified from the data. The article demonstrates how the IPTs were gleaned from the literature review and refined through partners’ interviews.  We sought the help of a realist expert who helped with the reanalysis of the interview data and restructuring of the findings section and also visualisation of the methodology |
| 14 | There are other specific comments in various parts of the paper that have been uploaded in the pdf. | All the comments made on the PDF document (mostly grammar and sentence structuring) have been addressed accordingly.  Grammarly was used to check and correct any grammar and punctuations errors. |
